# IMiD/CELMoD resistance in myeloma is associated with changes in lipid metabolism that may be targetable

**DOI:** 10.1016/j.bneo.2026.100281

**Published:** 2026-08-11

**Authors:** Sarah A. Bird, Marco P. Licciardello, Ananya Sukhtankar, Salomon Morales, Fernando J. Sialana, Yitao Xu, Amy Wilson, Harvey Che, Pradeep Ramagiri, Yakinthi Chrisochoidou, Yigen Li, Shannon Martin, Enze Liu, Brian Walker, Jyoti Choudhary, Graham Jackson, Martin Kaiser, Habib Bouguenina, Paul A. Clarke, Hector Keun, Charlotte Pawlyn

**Affiliations:** 1 The Institute of Cancer Research, London, United Kingdom; 2 The Royal Marsden NHS Foundation Trust, London, United Kingdom; 3 Imperial College London, London, United Kingdom; 4 Sylvester Comprehensive Cancer Center, University of Miami Health System, Miami, FL; 5 Northern Institute for Cancer Research, Newcastle University, Newcastle, United Kingdom

## Abstract

Immunomodulatory drugs (IMiDs) are a cornerstone of multiple myeloma treatment, and newer cereblon E3 ligase modulatory drugs (CELMoDs) are in clinical trials. However, a major barrier to improving patient outcomes is the inevitable development of resistance to these agents. To explore the mechanisms underlying IMiD/CELMoD resistance and identify novel targets for treatment, human cell line models of acquired IMiD/CELMoD resistance were generated. A quantitative proteome analysis of these models identified common changes in lipid synthesis proteins, and glucose-labeling experiments confirmed altered lipid flux. Proteomic analysis of paired patient samples at diagnosis and relapse on IMiD suggested similar changes in lipid pathways. A genome-wide CRISPR screen performed in a human multiple myeloma cell line with acquired CELMoD resistance identified dependencies in the lipid pathway genes stearoyl-CoA desaturase and membrane-bound transcription factor peptidase, site 1. This study has led to novel insights into lipid pathway changes in the IMiD/CELMoD-resistant state, which may represent targetable cancer cell vulnerabilities.

## Key Points

• Human myeloma cell line models of acquired IMiD/CELMoD resistance show alterations in lipid metabolism.
• A genome-wide CRISPR screen performed in a model of acquired resistance identified dependencies in two lipid pathway genes.

## Visual Abstract

**Figure:**
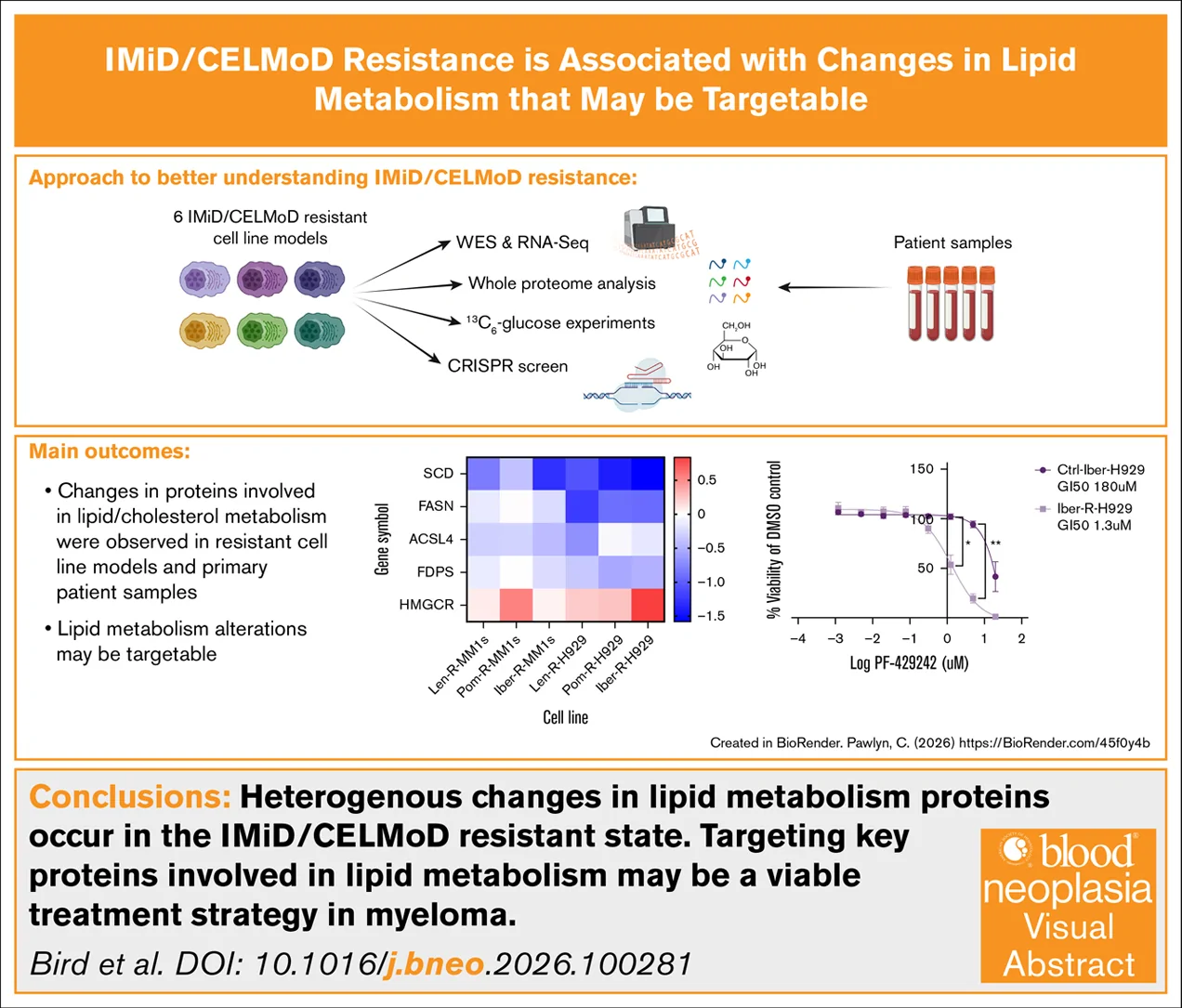

## Introduction

Immunomodulatory drugs (IMiDs; thalidomide, lenalidomide [Len], and pomalidomide [Pom]) and cereblon E3 ligase modulatory drugs (CELMoDs; iberdomide [Iber] and mezigdomide [Mezi]) form a critical component of many of the standard and experimental therapeutic combinations used to treat multiple myeloma (MM). In addition, the IMiD Len is widely used as a single-agent maintenance therapy after autologous stem cell transplantation (ASCT). However, patients inevitably become resistant to these agents, and understanding this process is vital to advancing targeted treatment strategies.

IMiDs and CELMoDs bind to a specific tritryptophan pocket of cereblon (CRBN), a substrate receptor of the cullin4-RING E3 ubiquitin ligase. Acting as molecular glues, these compounds bind to CRBN, reshaping its substrate repertoire and promoting ubiquitination of alternative proteins, also known as neosubstrates, which are subsequently targeted for proteasomal degradation. In myeloma cells, the critical neosubstrates are the B-cell zinc finger transcription factors Ikaros and Aiolos.^1^^,^^2^ Their rapid degradation leads to decreased expression of Interferon Regulatory Factor 4 (IRF4) and cellular MYC Proto-Oncogene (c-MYC), which, in turn, leads to growth inhibition and cell death.^3^ CELMoDs have enhanced affinity for binding CRBN and are better able to induce the closed (active) conformation of CRBN, leading to enhanced ubiquitination.^4^^,^^5^

Resistance to IMiDs and CELMoDs may be intrinsic to the myeloma cell and/or related to changes in the microenvironment. The mechanisms driving IMiD/CELMoD resistance in myeloma cells have been intensely studied and include *CRBN* mutations and copy number loss,^6^ methylation and silencing of the *CRBN* promoter,^7^ and differential exon 10 splicing of the CRBN transcript.^8^ In addition, there are resistance mechanisms related to changes in other components of the degradation machinery (eg, the COP9 signalosome^9^) and members of the Ikaros/Aiolos-IRF4-c-MYC pathway.^10^ IMiD/CELMoD resistance may also be related to transcriptional plasticity, with the expression of extralineage transcription factors. For example, increased occupancy of the developmentally associated ETS Variant Transcription Factor 4 (ETV4) transcription factor at gene enhancers in myeloma cells maintains transcription of c-MYC and other oncogenes despite ongoing degradation of Ikaros and Aiolos.^11^

Despite this active research, many of the molecular mechanisms behind IMiD/CELMoD resistance remain poorly understood. Here, we further explore mechanisms of resistance by performing multiomic analyses of cell line models of acquired IMiD/CELMoD resistance and demonstrate changes in lipid metabolism that may represent novel and targetable vulnerabilities.

## Materials and methods

### Cell culture and compound acquisition

Human MM cell lines MM1s and NCI-H929 were cultured in RPMI-1640 (catalog no. 61870, Gibco, Grand Island, NY), and HEK293 cells were cultured in Dulbecco's Modified Eagle Medium (catalog no. 31966, Gibco). Media were supplemented with 10% fetal bovine serum (catalog no. A5256801, Gibco) and 50 U/mL penicillin/streptomycin (catalog no. 15140122, Gibco). Cells were maintained in a humidified 37°C incubator with 5% carbon dioxide. Cells were authenticated using Short Tandem Repeats (STR) typing (Eurofins, Luxembourg City, Luxembourg) and confirmed to be mycoplasma negative (MycoStrip, InvivoGen, San Diego, CA).

IMiD/CELMoD-resistant cell lines (Len-R-MM1s, Pom-R-MM1s, Iber-R-MM1s, Len-R-H929, Pom-R-H929, and Iber-R-H929) were generated by long-term, low-dose exposure to their respective IMiD/CELMoD. Resistance was acquired gradually, and cells were then maintained in culture with the same dose: Len-R-MM1s/Len-R-H929 with 1μM Len, Pom-R-MM1s with 0.1μM Pom, Pom-R-H929 with 1μM Pom, and Iber-R-MM1s/Iber-R-H929 with 0.01μM Iber. Matched control lines were generated at the same dimethyl sulfoxide (DMSO) concentration for each resistant cell line (named Ctrl-Len-MM1s etc). An isogenic CRBN knockout (KO) line was used for comparison, generated as previously reported.^12^

Len, Pom, Iber, and Mezi were synthesized in-house using literature procedures. PF-429242 (catalog no. HY-13447) and SSI-4 (catalog no. HY-155033) were purchased from MedChemExpress (Monmouth Junction, NJ), and lovastatin (catalog no. S2061) was purchased from Selleck Chemicals (Houston, TX).

### Cell viability assays

Cell viability was assessed using CellTiter-Blue (catalog no. G8081, Promega, Madison, WI). Cells were seeded and treated in 96-well plates with serial dilutions of the respective drug and incubated for 120 hours. Differences between the cell lines were analyzed using unpaired *t* tests in GraphPad Prism version 11.0.0.

Combination assays with Iber plus lovastatin or PF-429242 were performed. Lovastatin/PF-429242 was used at 5μM in MM1s, and 1.25μM was used in H929. Iber was tested across 8 1:4 serial dilutions, with a top concentration of 2μM.

### Western blot analysis

Cells were lysed in 2% sodium dodecyl sulfate buffer, and protein concentration was measured by bicinchoninic acid (BCA) protein assay (catalog no. 23225, Thermo Fisher Scientific, Waltham, MA). Equal amounts of protein were separated by sodium dodecyl sulfate–polyacrylamide gel electrophoresis and transferred onto polyvinylidene fluoride (PVDF) membrane (catalog no. 88518, Thermo Fisher Scientific). Immunoblot analysis was performed with antibodies targeting CRBN (71810, Cell Signaling Technologies [CST], Danvers, MA, Research Resource Indentifier [RRID]: AB_2799810), Ikaros (14859, CST, RRID: AB_2744523), Aiolos (15103, CST, RRID: AB_2744524), and glyceraldehyde-3-phosphate dehydrogenase (GAPHD, 5174, CST, RRID: AB_10622025). Western blots were visualized using an Odyssey Fc Imager (LI-COR, Lincoln, NE) with IRDye infrared dyes as secondary antibodies. Densitometry analysis was performed using ImageStudio (LI-COR Biosciences).

### Whole-exome sequencing

DNA was extracted from cell lines using the AllPrep DNA/RNA Mini Kit (catalog no. 80204, Qiagen, Hilden, Germany) and underwent whole-exome sequencing (BGI, Shenzhen, China). Cell lines were sequenced at >130× depth. Samples were preprocessed and analyzed using the nfcore/sarek 2.7.1 pipeline according to Genome Analysis Toolkit best practices. Short variants were called using MuTect2 and annotated with Variant Effect Predictor.^13^

### Patient samples

Paired diagnosis and relapse (on Len) samples from 4 patients enrolled in the Myeloma XI trial were analyzed. Trial design and outcomes have been reported previously.^14^ Briefly, patients who were eligible for transplant were randomized to induction therapy with cyclophosphamide, thalidomide and dexamethasone; cyclophosphamide, Len, and dexamethasone; or carfilzomib combined with cyclophosphamide, Len, and dexamethasone and underwent ASCT. One hundred days after ASCT, eligible patients underwent further randomization to Len, Len/vorinostat, or observation maintenance therapy. Patients provided written informed consent, and the study was approved by the Oxfordshire Research Ethics Committee (MREC 17/09/09; ISRCTN49407852).

Myeloma tumor cells were immunomagnetically purified (>95%) from bone marrow using anti-CD138 antibodies (Miltenyi Biotec, Bergisch Gladbach, Germany). Copy number aberrations and immunoglobulin translocations were profiled using multiplex ligation-dependent probe amplification (P425-B1 MM probe mix; MRC Holland, Amsterdam, The Netherlands) and quantitative reverse transcription polymerase chain reaction (PCR).

### Proteomics

Quantitative proteomics of cell lines and patient samples was performed by tandem mass tag–based mass spectrometry at the Institute of Cancer Research (ICR) Proteomics Facility. Cell samples were harvested, washed twice with cold phosphate-buffered saline, snap-frozen on dry ice, and stored at −80°C. Samples were processed based on the SimPLIT workflow (supplemental Methods).^15^ In summary, cell pellets were lysed, and protein concentration was quantified. Aliquots containing 15 μg total protein were digested, and the resultant peptides were labeled with tandem mass tags. Peptides were fractionated, and liquid chromatography-mass spectrometry was performed. The SEQUEST-HT search engine was used to analyze the acquired mass spectra for protein identification and quantification. Spectra were searched against the UniProt-SwissProt proteomes of *Homo sapiens* protein entries, appended with contaminants. Normalized protein abundance values for resistant vs control comparisons were analyzed using a moderated *t* test in limma (implemented in Phantasus).^16^

Overrepresentation analysis of protein sets was performed in WebGestalt (https://www.webgestalt.org/) using the Fisher exact test and Kyoto Encyclopedia of Genes and Genomes (KEGG) pathway gene sets.^17^

### Labeled glucose experiments

Cells were incubated with media containing labeled ^13^C_6_-glucose (2 mg/mL) for 24 or 48 hours, and 1 × 10^6^ cells were seeded in 6-well plates for each condition and harvested at the specified time point. A full description of the method is provided in the supplemental Methods, but in brief, methanol quenching and extraction were performed, followed by a dual-phase extraction to separate and enrich nonpolar metabolites (such as fatty acids) for further analysis. Gas chromatography–mass spectrometry (GC-MS) analysis was performed, and metabolites were identified using an in-house library. Two parameters that describe fatty acid synthesis were estimated: (1) the proportion of lipogenic acetyl coenzyme A (CoA) derived from the 13C tracer (parameter D) and (2) the fraction of fatty acids that are synthesized de novo during the incubation (g(t)).^18^ Differences between the cell lines were analyzed using paired *t* tests in GraphPad Prism version 11.0.0.

### Genome-wide CRISPR-Cas9 screen

Iber-R-MM1s were transduced with the pLEX_311-Cas9v2 construct (catalog no. 96924, Addgene, Watertown, MA) to provide stable expression of Cas9. Cas9 activity was assessed using the pXPR-011-sgEGFP construct (catalog no. 59702, Addgene), which allows for the expression of enhanced green fluorescent protein (EGFP) and a single-guide RNA targeting the *EGFP* gene. The percentage of fluorescent cells was assessed by flow cytometry on a BD LSR II (BD Biosciences, Franklin Lakes, NJ) 8 to 10 days after infection. Cas9 endonuclease activity was considered sufficient when EGFP^+^ cells amounted to <25%.

The Brunello human CRISPR knockout (KO) pooled library (catalog no. 73178, Addgene) was expanded, and homogeneous library representation was confirmed using next-generation sequencing. Enough cells were seeded and kept in culture to ensure 500× coverage of the Brunello library throughout the entire pipeline. After 24 hours, cells were transduced with the Brunello library to achieve a multiplicity of infection of 0.3 to 0.5, and selection with puromycin was initiated 24 hours later. Five days later, the selected cells were split into 2 branches, 1 of which was treated with Iber (final concentration, 0.01μM) and the other with a matched concentration of DMSO. Drug treatment was continued for ∼8 cell doublings, with cells split when confluent (every 5 days). Biological replicates were performed as independent infections of cells with the library on distinct days.

At the end of the screen, genomic DNA was extracted from stored cell pellets using the Blood and Cell Culture DNA Maxi Kit (catalog no. 13362, Qiagen). PCR was performed using Herculase II Fusion DNA Polymerase (catalog no. 600675, Agilent Technologies) with an initial PCR to amplify the guide RNAs from the genomic DNA, and a second PCR to attach Illumina adaptors and to barcode the samples. The PCR products from each sample were quantified by agarose gel, pooled in equimolar amounts, and purified using the QIAquick Gel Extraction Kit (catalog no. 28104, Qiagen). Sequencing was then performed on a Illumina NovaSeq 6000 using a 100-bp single-end protocol (Source BioScience, Nottingham, United Kingdom).

Sequencing data were processed using PoolQ to demultiplex samples and generate an sgRNA count table for downstream analysis. The STARS algorithm was used to identify genes that, when knocked out, led to resensitization to Iber.^19^ The Chronos algorithm was employed to calculate gene effect scores. This algorithm infers gene KO fitness effects based on a model of cell proliferation dynamics after CRISPR gene KO.^20^ A lower score means a gene is more likely to be dependent, with a score of 0 indicating that a gene is not essential and a score of −1 representing the median of all common essential genes. The gene effect scores for the Iber-R-MM1s cell line were then compared with publicly available gene effect scores for parental MM1s (available via the Broad Institute DepMap portal and calculated in the same way from the CRISPR screen data). A novel dependency was defined as a score of <−1 in Iber-R-MM1s and >−0.5 in parental MM1s.

### Clonal competition assay

Clonal competition assays were performed based on the protocol from Girish et al.^21^ Guides were cloned into the LRG2.1 vector (catalog no. 108098, Addgene), which encodes a guide RNA and green fluorescent protein (GFP). This included guides targeting *MBTPS1* (AGAACTCTCTTGCTTCGCGG, GCTCAAACATGTTGACCCCG), *SCD* (ATGATCAGAAAGAGCCGTAG, CAGGTGTAGAACTTGCAGGT), *RPA3* (GATGAATTGAGCTAGCATGC), and *PKHD1L1* (ACGCCCACTTATTGTCAGCG, ATAACGACCACAGTAAGCAT). An empty LRG2.1 vector was used as a nontargeting control. Cas9-expressing cell lines (Cas9-Ctrl-Iber-MM1s and Cas9-Iber-R-MM1s) were then transduced.

An infection efficiency of ∼50% was desirable at baseline so that half of the cells had KO of the gene of interest and half of the cells were wild type (however, transduction efficiencies ranging from 10%-80% are suitable). The relative abundance of the transduced population was then measured over time via flow cytometry for GFP expression.

### Apoptosis assay

Cells were treated with lovastatin, PF-429242, and SSI-4 at 1.25μM, 5μM, and 20μM for 5 days. Cells were then harvested and stained with allophycocyanin-conjugated annexin V (catalog no. 550475, BD Biosciences) and/or propidium iodide (PI) (catalog no. BMS500PI, Thermo Fisher Scientific) as per the manufacturer's instructions. Annexin/PI-positive cells were analyzed using flow cytometry (Accuri C6 Plus, BD Biosciences) within 1 hour. Differences between the cell lines were analyzed using unpaired *t* tests in GraphPad Prism version 11.0.0.

## Results

### Generation of IMiD/CELMoD-resistant cell lines

IMiD/CELMoD resistance was achieved in the cell line models after 12 to 14 weeks of a fixed low-dose drug exposure (∼10× the 50% Growth Inhibition (GI50) concentration for each compound) in all except Pom-R-H929, in which Pom concentration escalation to 1μM was required to achieve resistance at 20 weeks (Figure 1A). Matched control lines were generated at the same dimethyl sulfoxide (DMSO) concentration for each resistant cell line (named Ctrl-Len-MM1s etc). All resistant models demonstrated cross-resistance to Len, Pom, Iber, and the more potent CELMoD Mezi, except Len-R-H929, in which Iber and Mezi could partially overcome resistance (supplemental Figure 1). No cross-resistance was seen with other anti-myeloma agents when tested in Iber-R-MM1s (supplemental Figure 2). Resistance was acquired gradually, consistent with pathway alteration as opposed to a single clone selection event (supplemental Figure 3).

**Figure 1.**
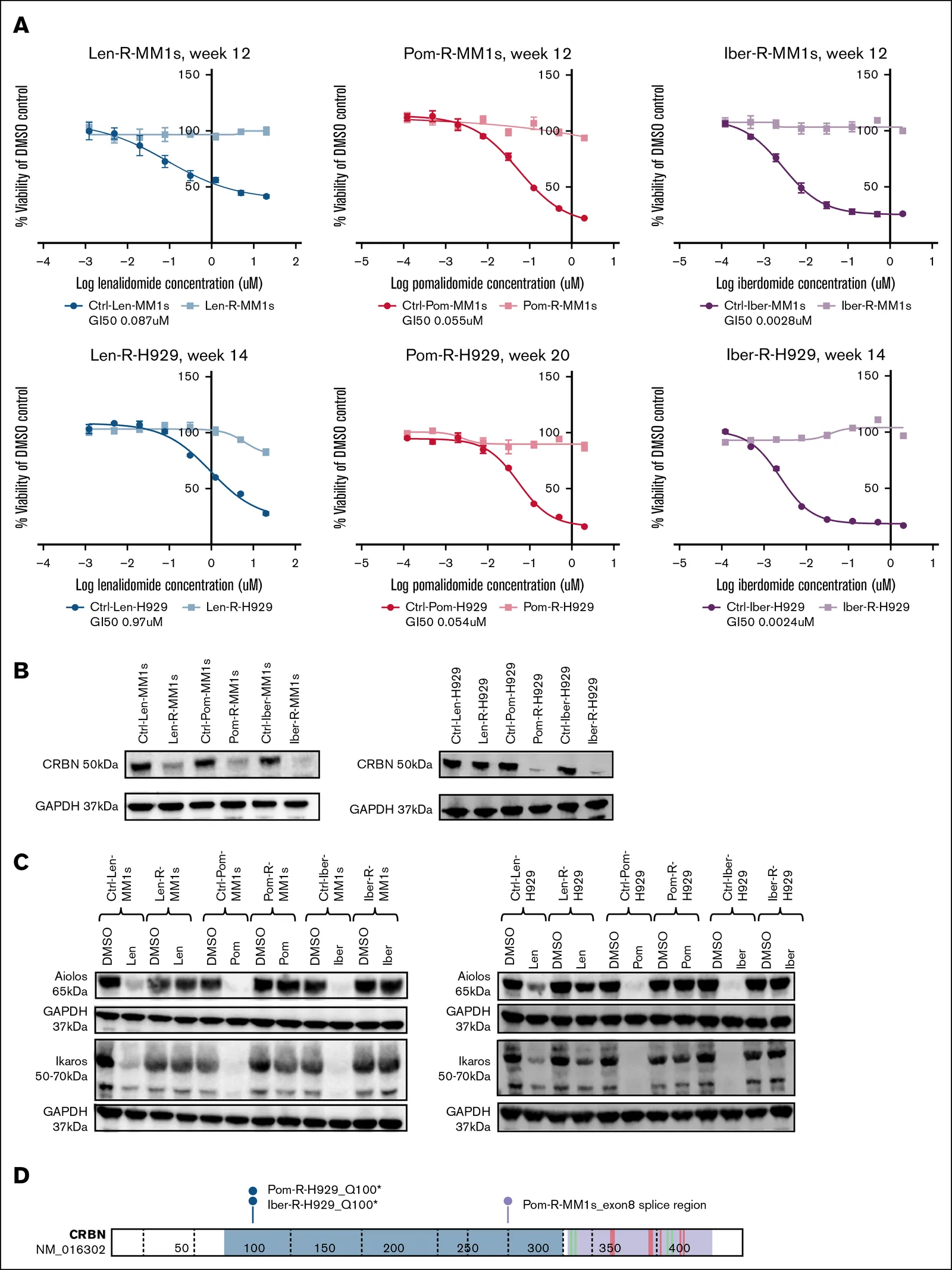
**Characterization of IMiD/CELMoD-resistant cell lines.** (A) Results of cell viability assays (using CellTiter-Blue) after 5 days of incubation with IMiD/CELMoD at the indicated week after commencing exposure to Len/Pom/Iber. Results for each cell line are normalized to DMSO treatment control. The mean and range of 3 technical replicates are shown. (B) Immunoblots demonstrating a reduction in CRBN protein expression in resistant cell lines. The images shown are representative of 2 biological replicates. (C) Immunoblots demonstrating Aiolos and Ikaros degradation in the control lines with IMiD/CELMoD treatment but abrogation of this effect in the resistant lines. The images shown are representative of 3 biological replicates. (D) Diagram representing whole-exome sequencing results for the *CRBN* gene. Pom-R-H929 and Iber-R-H929 had an early stop codon mutation, and Pom-R-MM1s had a splice site mutation, as indicated. LON substrate binding domain (blue), thalidomide binding domain (purple), tritryptophan IMiD binding pocket (red), zinc ion binding site (green). Figure created with ProteinPaint (https://proteinpaint.stjude.org). Ctrl, control; DMSO, dimethyl sulfoxide; GAPDH, glyceraldehyde-3-phosphate dehydrogenase; GI50, 50% Growth Inhibition.

Characterization of the cell lines demonstrated downregulation of CRBN protein expression, greater with Pom and Iber than with Len (Figure 1B; supplemental Figure 4), and loss of CRBN function, demonstrated by a reduction in Ikaros and Aiolos degradation (Figure 1C; quantification, supplemental Figure 5). DNA sequencing analysis of the resistant cell line pairs demonstrated *CRBN* mutations in Pom-R-MM1s, Pom-R-H929, and Iber-R-H929 (Figure 1D; supplemental Table 1). Pom-R-MM1s had a mutation that created a splice site variant, and Pom-R-H929 and Iber-R-H929 had the same mutation, which created an early stop codon. These mutations were all predicted to have a high impact on the resultant protein (by Ensembl Variant Effect Predictor) and lead to degraded mRNA or nonfunctional CRBN. No high-impact mutations in other IMiD pathway genes were observed in any of the cell lines (supplemental Table 2). Copy number loss at the *CRBN* locus was explored; Pom-R-H929 had the largest copy number loss, with Len-R-H929 showing copy number loss to a lesser degree (supplemental Figure 6). Other previously reported areas of loss (*COPS7B* and *COPS8* at Ch2q37^9^) did not show any substantial copy number change.

### Analysis of IMiD/CELMoD-resistant cells by proteomics

As the mechanism of action of IMiDs and CELMoDs is via protein degradation, whole proteome analysis was undertaken to interrogate possible resistance mechanisms. Downregulation of CRBN protein across all cell lines (Figure 2A) was confirmed. The only other proteins commonly altered in all cell lines were stearoyl-CoA desaturase (SCD; downregulated in resistant cell lines) and family with sequence similarity 169 member A (FAM169A; upregulated). Across ≥4 of the 6 pairs, 14 proteins were commonly upregulated, and 20 proteins were downregulated (log_2_ fold change [FC], ≥0.2 or ≤−0.2; adjusted *P* < .05; supplemental Table 3). There was a common alteration of several proteins involved in lipid synthesis, including SCD, fatty acid synthase, acyl-CoA synthetase long-chain family member 4, farnesyl diphosphate synthase, and HMG-CoA reductase (Figure 2B). Overrepresentation analysis of the top 100 proteins significantly downregulated in each resistant cell line also highlighted pathways involved in fatty acid synthesis in all lines, except Pom-R-MM1s (Figure 2C and supplemental Figure 7). These changes were not replicated on CRBN KO (supplemental Table 5 and supplemental Figure 8), suggesting that they are not directly driven by acute loss of CRBN but are likely to be influenced by CRBN-dependent or CRBN-independent pathway rewiring over the time course of resistance acquisition.

**Figure 2.**
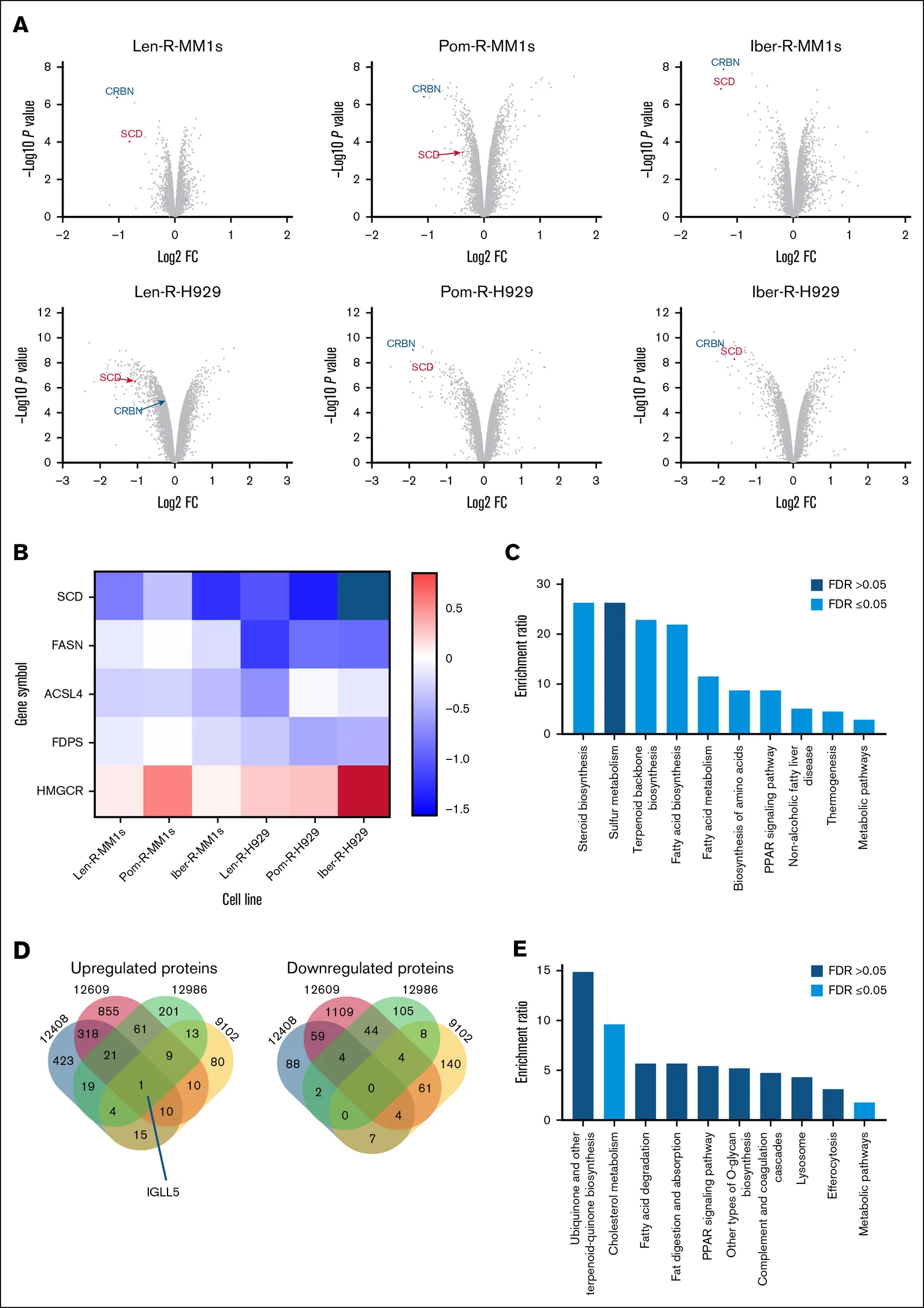
**Whole proteome analysis of IMiD/CELMoD-resistant cell lines and patient samples.** (A) Volcano plots of proteome data showing the differential expression of proteins in the IMiD/CELMoD-resistant cell lines (compared with a representative control line, Ctrl-Iber-MM1s for the MM1s lines and Ctrl-Iber-H929 for the H929 lines). CRBN and SCD were significantly downregulated (adjusted *P* < .05) in each of the resistant lines. (B) Heat map showing the log_2_ FC of lipid pathway proteins in the resistant cell lines compared with control. The proteins shown had adjusted *P* < .05 and a log_2_ FC of ≥0.2 or ≤−0.2 in ≥4 cell lines. (C) The overrepresentation analysis of the top 100 proteins significantly downregulated in Iber-R-MM1s (adjusted *P* < .05) highlighted steroid biosynthesis and fatty acid pathways. (D) Venn diagrams showing the overlap between proteins from patients with a log_2_ FC of >1 (increased at relapse compared with diagnosis) and <−1 (decreased at relapse compared with diagnosis). IGLL5 was commonly upregulated in all 4 patients. There were no commonly downregulated proteins in all patients. (E) Overrepresentation analysis of proteins altered with a log_2_ FC <−1 in at least 2 patients (n = 193) revealed that KEGG pathway cholesterol metabolism was significantly altered (FDR, ≤0.05). ACSL4, acyl-CoA synthetase long-chain family member 4; FASN, fatty acid synthase; FDPS, farnesyl diphosphate synthase; FDR, false discovery rate; HMGCR, HMG-CoA reductase; PPAR, peroxisome proliferator–activated receptors; IGLL5, immunoglobulin λ-like polypeptide 5.

Separate analyses comparing the Len-resistant cell lines (Len-R-MM1s and Len-R-H929), Pom-resistant lines (Pom-R-MM1s and Pom-R-H929), and Iber-resistant lines (Iber-R-MM1s and Iber-R-H929) showed additional common upregulated and downregulated proteins specific to each agent (supplemental Table 4). No changes in IMiD pathway proteins, except for CRBN, were observed in any of the cell line pairs.

To complement the whole proteome data, RNA sequencing analysis of the IMiD/CELMoD-resistant cell lines was also performed. The transcriptomes of each resistant cell line and its control line were compared, and genes for which mRNA expression was altered (log_2_ FC, ≥0.2 or ≤−0.2; adjusted *P* < .05) were selected. The sets of genes this generated per line were then compared. No genes were commonly upregulated in all 6 cell lines, but 17 genes were commonly upregulated in 5 of the lines (supplemental Table 6). *CRBN* was the only gene commonly downregulated in all 6 cell lines, but 9 genes were commonly downregulated in 5 lines (supplemental Table 6). Overrepresentation analysis was performed for each cell line individually, exploring the most upregulated and downregulated transcripts (supplemental Table 7). No common pathways between resistant cell lines were observed, including no changes in lipid pathways at the RNA level. This highlights the importance of translation control, posttranslational modifications, and protein stability in the expression levels of lipid pathway proteins.

To understand whether these newly identified protein changes found in cell line models were likely to be relevant to IMiD/CELMoD-resistant patients, proteomic analysis of paired diagnosis and relapse (on Len) samples from 4 patients who had been enrolled in the Myeloma XI trial was undertaken (supplemental Table 8). Expression of immunoglobulin λ-like polypeptide 5 (IGLL5) was upregulated in all patients at relapse (Figure 2D), consistent with previous findings.^22^ CRBN protein expression was decreased at relapse in 2 patients (log_2_ FC, −0.72 and −0.15). SCD was downregulated at relapse in the patient with the largest reduction in CRBN expression (log_2_ FC, −0.28), but not in the other patients. In addition, overrepresentation analysis of proteins altered with a log_2_ FC <−1 in at least 2 patients (n = 193) revealed that KEGG pathway cholesterol metabolism was altered (Figure 2E), supporting the findings in cell line models.

### Lipidomic analysis of IMiD/CELMoD-resistant cell lines

Altered expression of key proteins involved in lipid synthesis was hypothesized to lead to changes in lipid levels and lipid flux through the cells (Figure 3A). Glucose-labeling experiments in Iber-R-MM1s, Iber-R-H929, and their matched control lines were undertaken. The proportion of de novo synthesized palmitate, stearate, and myristate (the salts and esters of palmitic acid, stearic acid, and myristic acid, respectively) was reduced in Iber-R-H929 compared with its control (Figure 3B). Likewise, the fraction of newly synthesized palmitate was decreased in Iber-R-MM1s compared with its control (however, the proportions of stearate and myristate were not altered significantly). The MM1s cell lines had a reduced fatty acid synthesis rate compared with the H929 lines and, consequently, a relatively low abundance of newly synthesized fatty acids, potentially making them less sensitive to changes in fatty acid synthesis flux. Taken together, these results suggest that fatty acid synthesis activity might be downregulated in the IMiD/CELMoD-resistant state.

**Figure 3.**
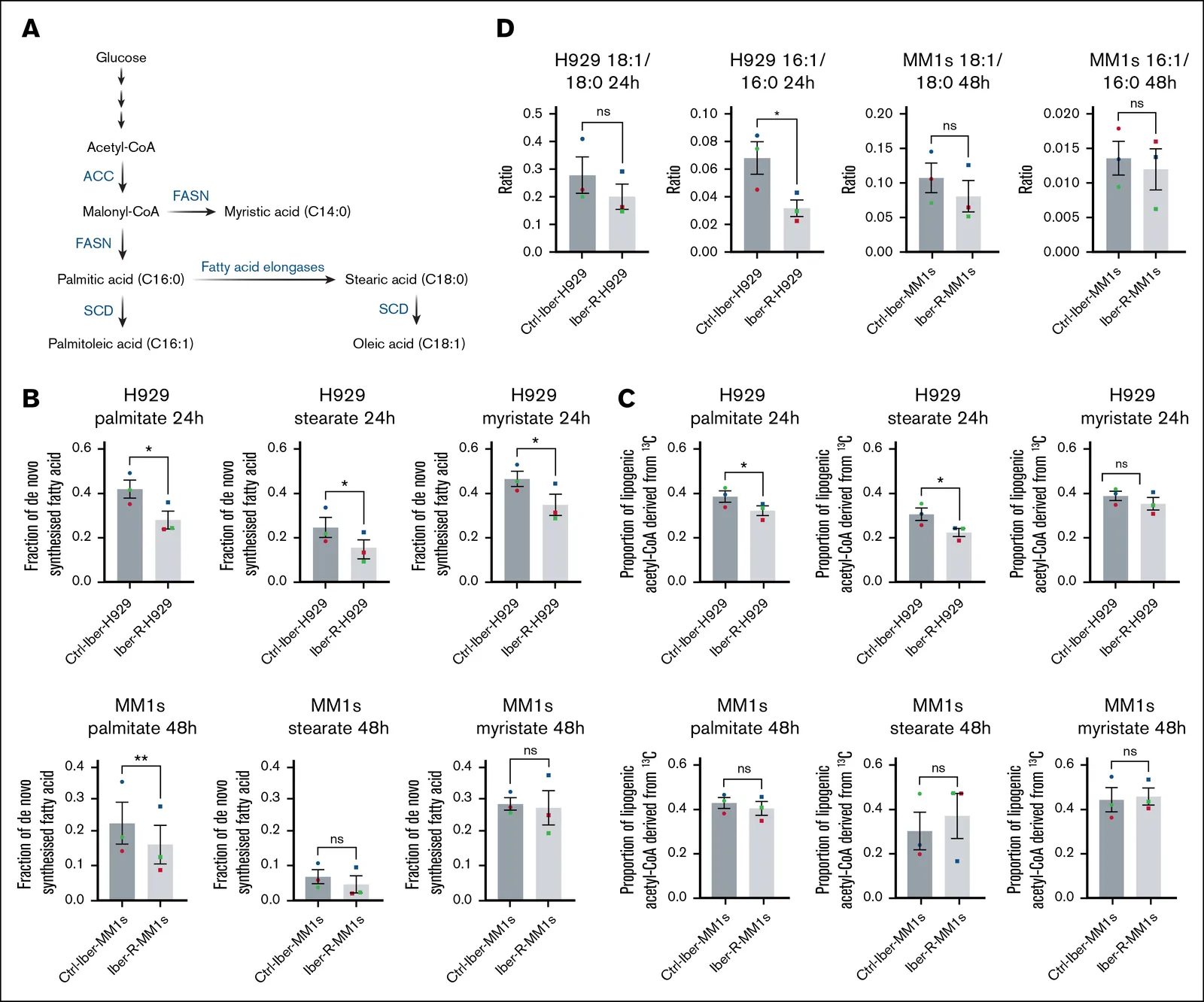
**Glucose-labeling experiments in IMiD/CELMoD-resistant cell lines.** (A) Pathway of lipid synthesis. Acetyl-CoA, the substrate of fatty acid synthesis, is generated predominantly from glucose via glycolysis and the tricarboxylic acid cycle (but also fatty acid and amino acid catabolism to a lesser extent). Acetyl-CoA is converted into malonyl-CoA by ACC, which is then used as a building block by the enzyme fatty acid synthase to produce myristic acid (C14:0), which is, in turn, elongated to give palmitic acid (C16:0). Palmitic acid can be elongated (by fatty acid elongases) to generate stearic acid (C18:0). The desaturase SCD catalyzes the desaturation of palmitic acid and stearic acid to generate palmitoleic acid (C16:1) and oleic acid (C18:1), respectively. C16:0 indicates that the fatty acid has a 16-carbon backbone and no double bonds (ie, saturated). Enzymes are shown in blue. (B) The fraction of each fatty acid that was synthesized de novo during incubation with labeled glucose is shown. N = 3 independent experiments shown; replicate 1, red; replicate 2, green; replicate 3, blue. The differences between the cell lines were analyzed using a paired *t* test; ∗*P* < .05, ∗∗*P* < .01. (C) The relative enrichment in precursor acetyl-CoA from the ^13^C-labeled glucose for each fatty acid is shown. N = 3 independent experiments shown; replicate 1, red; replicate 2, green; replicate 3, blue. The differences between the cell lines were analyzed using a paired *t* test; ∗*P* < .05. (D) The ratio of desaturated to saturated fatty acids for each cell line pair is shown; 18:1, oleate; 18:0, stearate; 16:1, palmitoleate; 16:0, palmitate. N = 3 independent experiments are shown; replicate 1, red; replicate 2, green; replicate 3, blue. The differences between cell lines were analyzed using a paired *t* test; ∗*P* < .05. ACC, acetyl-CoA carboxylase; Ctrl, control; ns, not significant.

The proportion of lipogenic acetyl-CoA (the substrate for fatty acid synthesis) derived from glucose was reduced in Iber-R-H929 compared with control (Figure 3C), suggesting that the proportional contribution of glucose to fatty acid synthesis was decreased, and alternative carbon sources might compensate. Furthermore, the ratios of oleate/stearate (18:1/18:0) and palmitoleate/palmitate (16:1/16:0) were decreased in Iber-R-H929, with a similar trend in Iber-R-MM1s, suggesting that the activity of fatty acid desaturation was reduced (Figure 3D). SCD catalyzes the conversion of saturated fatty acids into monounsaturated fatty acids; therefore, these data are consistent with the reduction in SCD abundance seen at the protein level.

### CRISPR screen to identify novel vulnerabilities in the IMiD/CELMoD-resistant state

Independently from the experiments described earlier, novel targets for therapy in the IMiD/CELMoD-resistant context were sought. A CRISPR screen workflow was designed to identify genes that, when knocked out, would reinduce sensitivity to Iber in Iber-R-MM1s (Figure 4A). However, no candidate genes were identified (false discovery rate <0.05). The Iber-R-MM1s cell line exhibited loss of CRBN (the IMiD/CELMoD target), and our data suggest that there is no single gene that, when knocked out in this low-CRBN state, leads to resensitization to IMiDs/CELMoDs. It might be that a gene needs to be upregulated to restore CRBN expression, or that a group of genes needs altered expression to restore sensitivity. As an alternative strategy, a comparison with data from previous CRISPR screens (DepMap data) for parental MM1s was undertaken, aiming to identify novel dependencies in the resistant state (Figure 4B). Forty-seven genes were identified (supplemental Table 9), including 2 genes involved in lipid pathway metabolism, *SCD*, and membrane-bound transcription factor peptidase, site 1 (*MBTPS1*). This suggests that the alteration of lipid pathways identified in the resistant state may also represent targetable vulnerabilities.

**Figure 4.**
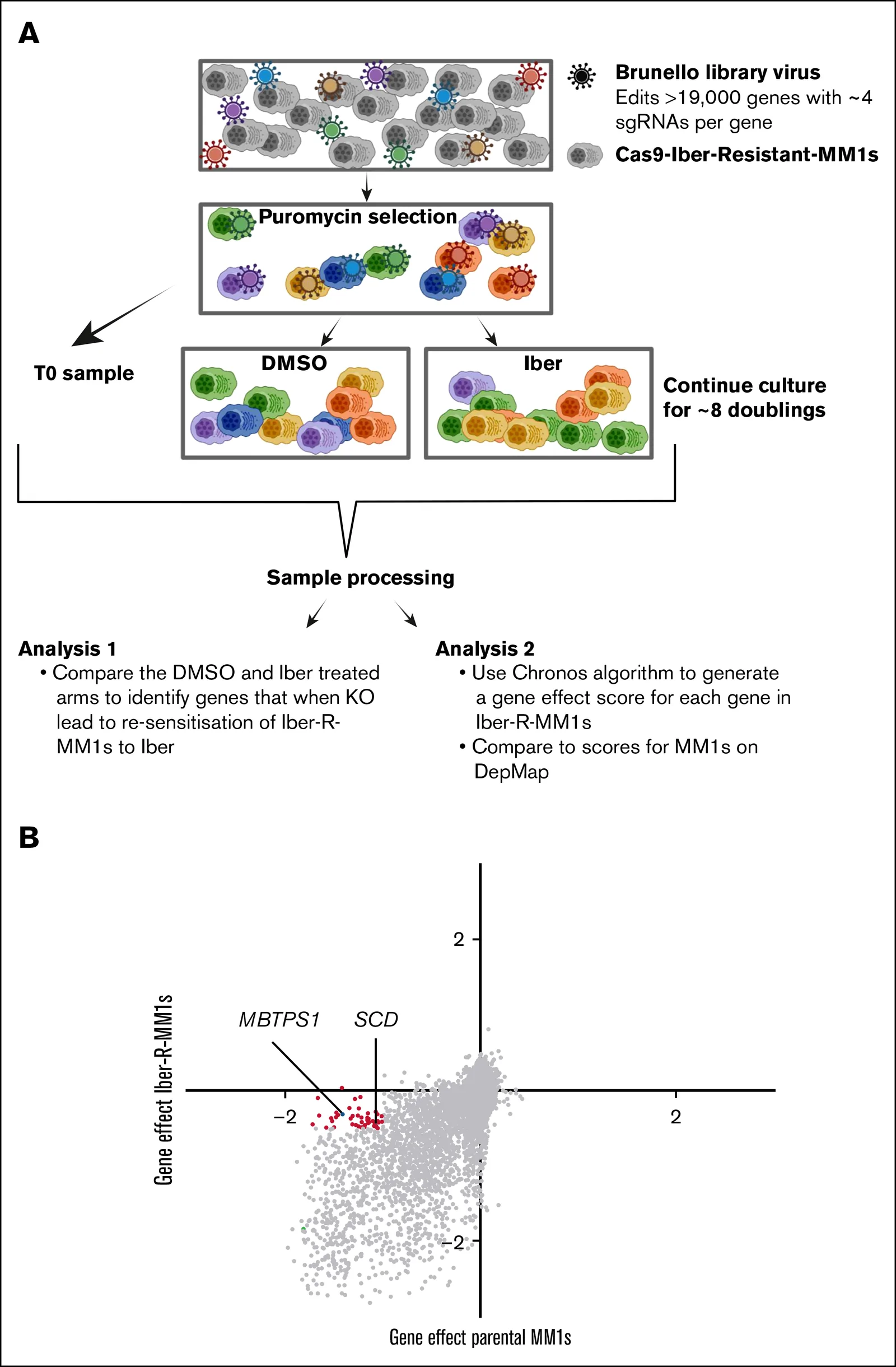
**CRISPR screen work flow and results.** (A) CRISPR screen workflow. After the transduction of Cas9-Iber-R-MM1s with the Brunello plasmid pool and subsequent puromycin selection, cells were treated with DMSO or Iber. Two separate analyses were then performed. Analysis 1 was designed to identify any genes that, when knocked out, reinduced sensitivity to Iber in Iber-R-MM1s. Analysis 2 used the Chronos algorithm to identify novel dependencies in Iber-R-MM1s compared with parental MM1s. Image created with biorender.com. (B) Graph showing the gene effect scores for Iber-R-MM1s on the x-axis and parental MM1s on the y-axis. The Chronos algorithm was used to calculate the gene effect scores of each gene in the Iber-R-MM1s cell line. These scores were then compared with those for parental MM1s using data from the DepMap portal that had been generated using the same method. A lower score means that a gene is more likely to be dependent in a given cell line. For example, *IRF4* has a score of −1.8 in both cell lines and, therefore, is essential in both cell lines. Potential novel dependencies were defined as a score of <−1 in the resistant line and >−0.5 in parental MM1s. This identified 47 genes, which are highlighted in red. Cas9, CRISPR-associated protein 9; DMSO, dimethyl sulfoxide; sgRNAs, single-guide RNAs.

### Exploration of potential targetable vulnerabilities in lipid synthesis pathways

Clonal competition assays in the Ctrl-Iber-MM1s and Iber-R-MM1s cell lines that stably expressed Cas9 confirmed that both lines were dependent on SCD and MBTPS1 (Figure 5A), but without a clear differential response. This may be related to the sensitivity of the assay to identify subtle differences.

**Figure 5.**
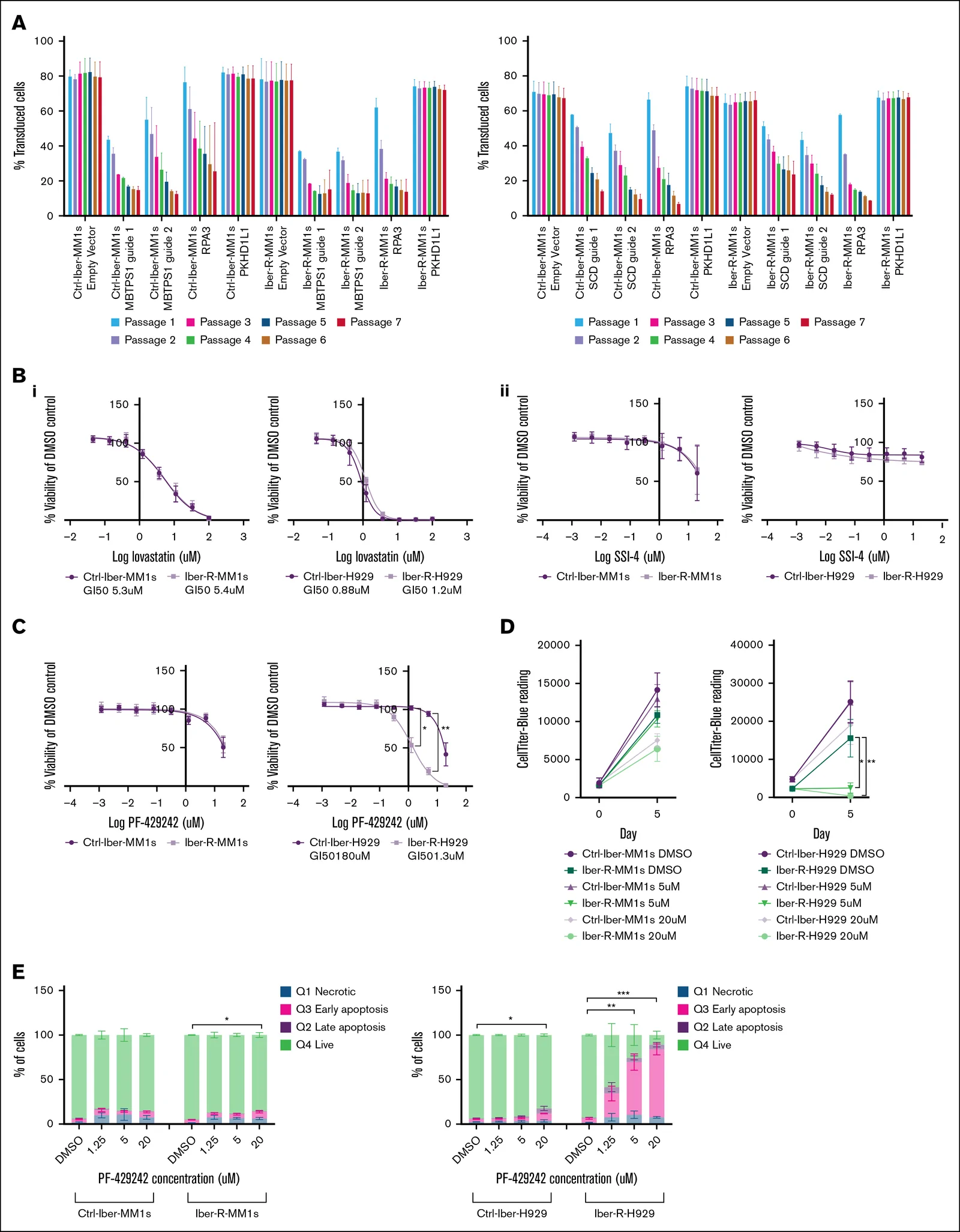
**Validation of potential targetable vulnerabilities in lipid synthesis pathways.** (A) MBTPS1 and SCD were confirmed as dependencies in both Ctrl-Iber-MM1s and Iber-R-MM1s. The percentage of transduced cells over time was assessed by flow cytometry (transduced cells were GFP^+^). N = 2; the bar represents the mean, and the error bars represent the standard error of the mean (SEM). (B) Cell viability assays (CellTiter-Blue) with indicated cell lines incubated with (i) lovastatin and (ii) SSI-4 for 5 days. Mean and SEM are shown (n = 3). (C) Cell viability assays (CellTiter-Blue) with indicated cell lines incubated with PF-429242 for 5 days. Mean and SEM are shown (n = 4). Pairwise comparisons between groups were performed using unpaired *t* tests; ∗*P* < .05 and ∗∗*P* < .01. (D) Cell viability assays (CellTiter-Blue) measured on days 0 and 5 to compare cell proliferation. Mean and SEM are shown (n = 3). Pairwise comparisons between groups were performed using unpaired *t* tests; ∗*P* < .05 and ∗∗*P* < .01. (E) Annexin/PI flow cytometry with indicated cell lines incubated with PF-429242 (1.25μM, 5μM, and 20μM) for 5 days. Mean and SEM are shown (n = 3). Pairwise comparisons between the proportion of cells in Q2 and Q3 in different groups were performed using unpaired *t* tests; ∗*P* < .05, ∗∗*P* < .01, and ∗∗∗*P* < .001. Ctrl, control; DMSO, dimethyl sulfoxide; GI50, 50% Growth Inhibition.

Available inhibitors were then explored in the cell line models. As HMG-CoA reductase had significantly increased expression in 4 of the 6 resistant cell lines, the effect on viability of the HMG-CoA reductase inhibitor lovastatin was explored. Lovastatin was active in the MM1s and H929 cell lines, but there was no difference in the response of the resistant cell lines and their respective controls (Figure 5Bi and supplemental Figure 9A). Lovastatin induced cell death, with evidence of induction of apoptosis, and the H929 cell lines were more sensitive than the MM1s cell lines (supplemental Figure 9B-C). Combination assays with Iber and lovastatin indicated that HMG-CoA reductase inhibition does not modulate CELMoD sensitivity (supplemental Figure 9D).

The SCD inhibitor SSI-4 only affected viability in the MM1s cell lines at the highest concentration tested (20μM), with no difference between sensitive and resistant cells. SSI-4 did not show a concentration-dependent effect on viability in the H929 cell lines (Figure 5Bii; supplemental Figure 10). Given the lack of single-agent activity, combination assays were not performed.

The MBTPS1 inhibitor PF-429242 reduced viability in the MM1s cell lines (both control and resistant) only at the highest concentration tested (20μM), with no differential response observed between control and resistant cell lines. H929 cell lines were more sensitive, and the Pom-R-H929 and Iber-R-H929 cell lines showed a greater reduction in viability than their sensitive counterparts (in keeping with the CRISPR screen data analysis performed in MM1s; Figure 5C and supplemental Figure 11A). The control and resistant MM1s cell lines showed a small reduction in growth with PF-429232, as did the control H929 cell line (Figure 5D). However, Iber-R-H929 showed a reduction in cell growth consistent with cytotoxicity (Figure 5D) and evidence of induction of apoptosis with PF-429242 in a concentration-dependent manner (Figure 5E). Combination assays with Iber and PF-429242 indicated that MBTPS1 inhibition does not modulate CELMoD sensitivity (supplemental Figure 11B).

## Discussion

This study provides insights into changes that occur in IMiD/CELMoD-resistant MM, showing that cell line models of acquired resistance, together with samples from Len-resistant patients, exhibited alterations in lipid metabolism. We propose that as cells become resistant to IMiD/CELMoDs through various mechanisms, including CRBN downregulation, there are concomitant changes in lipid biosynthesis. Furthermore, the inhibition of MBTPS1, a key regulator of cholesterol and fatty acid synthesis, reduced viability in resistant H929 models, suggesting that lipid metabolic pathways may represent therapeutically actionable vulnerabilities in certain cases of IMiD/CELMoD-resistant myeloma and merit further investigation.

Multiple models of IMiD/CELMoD resistance were generated using 2 human myeloma cell lines with different genetic backgrounds (MM1s and H929) and 3 different compounds (Len, Pom, Iber). Three of the cell lines had acquired mutations in the *CRBN* gene, and 2 showed copy number loss, reflecting the variation seen in patients. The reduction in CRBN abundance (seen in all cell lines but greatest in those with Iber resistance) was likely the major cause of resistance in the cell line models, given the central role of CRBN in the function of IMiDs/CELMoDs. Indeed, Len-R-H929 had only a small reduction in CRBN compared with its control line, and resistance could be overcome by the more potent CRBN binders.

The most prominent shared feature seen in whole proteome analysis of the resistant cell lines was the enrichment of lipid pathways within downregulated proteins. Furthermore, each of the lines showed a reduction in the level of the lipid pathway modulator SCD. Of note, these changes were not observed in an MM1s CRBN KO model, suggesting that changes in lipid metabolism may occur because of gradual pathway rewiring as the cells acquire resistance to IMiD/CELMoDs. As the alterations in lipid metabolism occurred in both IMiD-resistant and CELMoD-resistant lines, it suggests that certain features of resistance biology will be shared between the 2 groups of compounds, likely because of their shared mechanism of action. Changes in the expression of proteins involved in lipid/cholesterol metabolism were shown to be associated with a reduction in fatty acid synthesis in the resistant state, and there was also reduced fatty acid desaturation (in keeping with a reduction in SCD activity). It would be interesting to explore if the observed metabolic changes are stable or reversible upon withdrawal of IMiD/CELMoDs.

Importantly, proteomic analysis of paired patient samples at diagnosis and relapse (on Len) also highlighted changes in cholesterol metabolism. Although only 4 samples were analyzed, these early data support the hypothesis that patient myeloma cells acquire altered lipid metabolism during the development of IMiD resistance. Only 1 of the patient samples had a large reduction in CRBN at relapse, and it would be important to evaluate a larger group of patients to enable us to assess if patients with reduced CRBN levels at relapse exhibit more pronounced changes in SCD and lipid metabolism pathways than those without decreased CRBN. It would also be interesting to evaluate paired samples from patients exposed to more potent CRBN binders because the work described here would suggest that the altered lipid metabolism signature might be stronger: in our models, CELMoD resistance drives lower CRBN expression and more lipid pathway changes compared with IMiD resistance. As CELMoDs are being evaluated in clinical trials, both as components of combination therapies and as maintenance agents, opportunities for these analyses will be possible going forward.

Having identified changes in lipid metabolism, we sought to explore whether these alterations could be exploited for therapeutic benefit. In keeping with previous studies, we show that lovastatin reduces cell viability in myeloma cell lines.^23^ In retrospective registry studies, statin use has been shown to reduce MM-specific mortality,^24^^,^^25^ and therefore, exploring lipid metabolism targeting in MM is an important avenue of research. However, there was no difference in response between resistant and control cell lines, suggesting that this is not a novel vulnerability in the IMiD/CELMoD-resistant state.

An independently performed genome-wide CRISPR screen was used to identify genes for which KO reduced viability more in the IMiD/CELMoD-resistant state compared with the sensitive state (potentially representing vulnerabilities that may develop during resistance acquisition). The screen was performed in a cell line that was resistant to Iber and had very low levels of CRBN expression. Two of the 47 genes identified were involved in lipid metabolism, namely, SCD and MBTPS1. In a clonal competition assay, both genes were demonstrated to be necessary for myeloma cell survival. However, there was no clear difference in dependency for either gene between the Iber-R-MM1s and Ctrl-Iber-MM1s lines, possibly because of the sensitivity of the assay. Interestingly, treatment with a small-molecule inhibitor of MBTPS1 (PF-429242) led to a significantly greater reduction in viability in Pom-R-H929 and Iber-R-H929 lines compared with their respective controls. This tool compound is not ready for the clinical setting but indicates that the modulation of lipid metabolism pathways is a potential treatment avenue. MBTPS1 is a master regulator of cholesterol and fatty acid metabolism, initiating the activation of sterol regulatory element-binding proteins, which then drive the transcription of genes required for de novo lipogenesis and cholesterol synthesis. Targeting this part of the pathway, therefore, may have a greater impact than targeting individual downstream proteins. Attempts to genetically or pharmacologically validate the downstream protein SCD as a target were not successful, potentially because of the pharmacological properties of the SSI-4 inhibitor.

We propose that heterogeneous changes in lipid metabolism pathways occur in the IMiD/CELMoD-resistant state but that targeting key proteins involved in lipid metabolism may be a viable treatment strategy in MM. This is particularly important given the challenge of treating IMiD/CELMoD-resistant patients and the urgent need for new therapeutic approaches.

Conflict-of-interest disclosure: The Institute of Cancer Research has a commercial interest in the development of compounds targeting CRL4-CRBN E3 ubiquitin ligases. C.P. has received honoraria from Celgene/Bristol Myers Squibb (manufacturer of IMiDs/CELMoDs) for advisory boards, educational talks, and participation in data monitoring committees. The remaining authors declare no competing financial interests.

## Supplementary Material

Supplemental Methods, Tables, Figures, and References

## References

[1] Kronke J, Hurst SN, Ebert BL (2014). Lenalidomide induces degradation of IKZF1 and IKZF3. Oncoimmunology.

[2] Lu G, Middleton RE, Sun H (2014). The myeloma drug lenalidomide promotes the cereblon-dependent destruction of Ikaros proteins. Science.

[3] Shaffer AL, Emre NC, Lamy L (2008). IRF4 addiction in multiple myeloma. Nature.

[4] Hansen JD, Correa M, Nagy MA (2020). Discovery of CRBN E3 ligase modulator CC-92480 for the treatment of relapsed and refractory multiple myeloma. J Med Chem.

[5] Watson ER, Novick S, Matyskiela ME (2022). Molecular glue CELMoD compounds are regulators of cereblon conformation. Science.

[6] Gooding S, Ansari-Pour N, Towfic F (2021). Multiple cereblon genetic changes are associated with acquired resistance to lenalidomide or pomalidomide in multiple myeloma. Blood.

[7] Haertle L, Barrio S, Munawar U (2021). Cereblon enhancer methylation and IMiD resistance in multiple myeloma. Blood.

[8] Neri P, Maity R, Keats JJ (2016). Cereblon splicing of Exon 10 mediates IMiDs resistance in multiple myeloma: clinical validation in the CoMMpass trial. Blood.

[9] Gooding S, Ansari-Pour N, Kazeroun MH (2022;140(16):1816-1821). Loss of COP9-Signalosome genes at 2q37 is associated with IMiD agent resistance in multiple myeloma. Blood.

[10] Barrio S, Munawar U, Zhu YX (2020). IKZF1/3 and CRL4(CRBN) E3 ubiquitin ligase mutations and resistance to immunomodulatory drugs in multiple myeloma. Haematologica.

[11] Neri P, Barwick BG, Jung D (2024). ETV4-dependent transcriptional plasticity maintains MYC expression and results in IMiD resistance in multiple myeloma. Blood Cancer Discov.

[12] Chrisochoidou Y, Scarpino A, Morales S (2025). Evaluating the impact of CRBN mutations on response to immunomodulatory drugs and novel cereblon E3 ligase modulators in myeloma. Blood.

[13] McLaren W, Gil L, Hunt SE (2016). The Ensembl Variant Effect Predictor. Genome Biol.

[14] Jackson GH, Davies FE, Pawlyn C (2019). Lenalidomide maintenance versus observation for patients with newly diagnosed multiple myeloma (Myeloma XI): a multicentre, open-label, randomised, phase 3 trial. Lancet Oncol.

[15] Sialana FJ, Roumeliotis TI, Bouguenina H (2022). SimPLIT: simplified sample preparation for large-scale isobaric tagging proteomics. J Proteome Res.

[16] Kleverov M, Zenkova D, Kamenev V, Sablina M, Artyomov MN, Sergushichev AA (2024). Phantasus, a web based application for visual and interactive gene expression analysis. Elife.

[17] Elizarraras JM, Liao Y, Shi Z, Zhu Q, Pico AR, Zhang B (2024). WebGestalt 2024: faster gene set analysis and new support for metabolomics and multi-omics. Nucleic Acids Res.

[18] Tredwell GD, Keun HC (2015). convISA: a simple, convoluted method for isotopomer spectral analysis of fatty acids and cholesterol. Metab Eng.

[19] Doench JG, Fusi N, Sullender M (2016). Optimized sgRNA design to maximize activity and minimize off-target effects of CRISPR-Cas9. Nat Biotechnol.

[20] Dempster JM, Boyle I, Vazquez F (2021). Chronos: a cell population dynamics model of CRISPR experiments that improves inference of gene fitness effects. Genome Biol.

[21] Girish V, Sheltzer JM (2020). A CRISPR competition assay to identify cancer genetic dependencies. Bio Protoc.

[22] D'Agostino M, Zaccaria GM, Ziccheddu B (2020). Early relapse risk in patients with newly diagnosed multiple myeloma characterized by next-generation sequencing. Clin Cancer Res.

[23] van de Donk NW, Kamphuis MM, Lokhorst HM, Bloem AC (2002). The cholesterol lowering drug lovastatin induces cell death in myeloma plasma cells. Leukemia.

[24] Brånvall E, Ekberg S, Eloranta S, Wästerlid T, Birmann BM, Smedby KE (2020). Statin use is associated with improved survival in multiple myeloma: a Swedish population-based study of 4315 patients. Am J Hematol.

[25] Sanfilippo KM, Keller J, Gage BF (2016). Statins are associated with reduced mortality in multiple myeloma. J Clin Oncol.

