# Supplementary material for: IMiD/CELMoD resistance in myeloma is associated with changes in lipid metabolism that may be targetable
Source: Blood Neoplasia. 2026 Aug 11;3(4):100281. doi: 10.1016/j.bneo.2026.100281 (PMC13635510; doi:10.1016/j.bneo.2026.100281)
Supplement: Supplementary file 1 [file BNEO_NEO-2025-000970-mmc1.pdf]

Changes in lipid metabolism that occur with the acquisition of IMiD/CELMoD resistance in myeloma expose a vulnerability that may be targetable

Contents

Supplementary Methods 3
- Proteomics........ 3
- Labelled glucose experiments 4

Supplementary Tables 6
- Supplementary Table 1: Variant allele frequency of each CRBN mutation in the resistant line and its matched control line. 6
- Supplementary Table 2: Mutations identified in resistant cell lines. 6
- Supplementary Table 3: Common proteins with altered expression in the resistant cell lines. 7
- Supplementary Table 4: Common proteins with altered expression in the lenalidomide resistant cell lines, pomalidomide resistant cell lines and iberdomide resistant cell lines. 9
- Supplementary Table 5: Proteome changes in CRBN-KO-MM1s compared to parental MM1s. 17
- Supplementary Table 6: Common transcriptome changes in the resistant cell lines 18
- Supplementary Table 7: Over-representation analysis of transcripts with altered expression in the resistant cell lines. 20
- Supplementary Table 8: Patient characteristics of samples from the Myeloma XI trial 20
- Supplementary Table 9: Gene effect scores in Iber-R-MM1s (experimental data) and parental MM1s (DepMap) 21

Supplementary Figures 25
- Supplementary Figure 1: Viability assays demonstrating response of resistant lines A) MM1s and B) H929 to other CRBN binding agents 25
- Supplementary Figure 2: Viability assays demonstrating response of resistant line Iber-R-MM1s to other anti-myeloma agents 27
- Supplementary Figure 3: Time course of resistance acquisition in A) MM1s and B) H929 28
- Supplementary Figure 4: CRBN proteins levels 30
- Supplementary Figure 5: Western blot quantitation demonstrating loss of CRBN function in resistant cell lines 31

Supplementary Figure 6: Copy number loss at the *CRBN* locus in the resistant cell lines 32
Supplementary Figure 7: Over-representation analysis of proteins with reduced expression in the IMiD/CELMoD resistant cell lines.. 33
Supplementary Figure 8: Over-representation analysis of proteins with reduced expression in CRBN-KO-MM1s 35
Supplementary Figure 9: Viability assays demonstrating response of the resistant cell lines to lovastatin treatment.. 36
Supplementary Figure 10: Viability assays demonstrating response of the resistant cell lines to SSI-4 treatment 38
Supplementary Figure 11: Viability assays demonstrating response of the resistant cell lines to PF-429242 treatment 40

Supplementary Methods

Proteomics

Samples for quantitative proteomics were prepared using the SimPLIT workflow [1]. Briefly, cell pellets were lysed in a buffer containing 150μL 0.1M triethylammonium bicarbonate (TEAB), 1% sodium deoxycholate (SDC), 10% isopropanol, 50mM NaCl, 5mM tris-2-carboxyethyl phosphine (TCEP), 10mM iodoacetamide (IAA) and universal nuclease, supplemented with protease and phosphatase inhibitors. Samples were bath sonicated for 5 minutes, incubated at room temperature for 45 minutes and protein concentration was measured with the Bradford Protein Assay (Bio-Rad) according to the manufacturer's instructions. Proteins were digested with the addition of trypsin stock solution (500ng/μL in 0.1% formic acid, Pierce, 90059). The resultant peptides were labelled with a tandem mass tag (TMTpro) multiplex reagent vial (Thermo Fisher Scientific) according to the manufacturer's instructions. All of the TMT-labelled samples were combined in equal amounts to a single tube. The TMT-peptide mixture was acidified with 1% formic acid and the precipitated sodium deoxycholate (SDC) was removed by centrifugation. The sample was then dried with a centrifugal vacuum concentrator.

Offline peptide fractionation was based on high pH Reverse Phase (RP) chromatography using the Waters XBridge C18 column (2.1 × 150 mm, 3.5 μm) on a Dionex Ultimate 3000 HPLC system at a 0.85% gradient with flow rate 0.2 mL/min. Mobile phase A was 0.1% ammonium hydroxide, and mobile phase B was 100% acetonitrile, 0.1% ammonium hydroxide. Retention time-based fractions were collected and pooled into thirty samples for LC-MS analysis. LC-MS analysis was performed on the Dionex UltiMate 3000 UHPLC system coupled with the Orbitrap Ascend Tribrid mass spectrometer (Thermo Fisher Scientific). Samples were analysed with the PepMap C18 capillary column (75μm × 50cm, 2μm) at 50^{0}C.

Mobile phase A was 0.1% formic acid and mobile phase B was 80% acetonitrile, 0.1% formic acid. The gradient separation method was as follows: 150 minutes gradient up to 38% B, for 10 minutes up to 95% B, for 5 minutes isocratic at 95% B, re-equilibration to 5% B in 10 minutes, for 10 minutes isocratic at 5% B. Precursors between 375 and 1500 m/z were selected with a mass resolution of 120,000, automatic gain control (AGC) of 4 × 105, and IT (injection time) of 50 milliseconds, with the top speed mode in 3 seconds for high collision dissociation (HCD) fragmentation with a quadrupole isolation width of 0.7 Th (Thomson unit). The collision energy was set at 35%, with AGC at 1 × 105 and IT at 86 milliseconds. The HCD MS2 spectra were acquired with a fixed first mass at 100 m/z and a resolution of 45,000. Targeted precursors were dynamically excluded for further isolation and activation for 45 seconds with 7 ppm mass tolerance.

The SEQUEST-HT search engine was used to analyse the acquired mass spectra in Proteome Discoverer 2.4 (Thermo Fisher Scientific) for protein

identification and quantification. The precursor mass tolerance was set at 20 ppm, and the fragment ion mass tolerance was set at 0.5 Da. Spectra were searched for fully tryptic peptides with maximum 2 mis-cleavages. TMTpro on lysine residues and peptide N termini (304.2071 Da) and carbamidomethylation of cysteine residues (+57.0215 Da) were set as static modifications while oxidation of methionine residues (+15.9949 Da) and deamidation of asparagine and glutamine (+0.9848 Da) was set as a variable modification. Peptide confidence was estimated with the Percolator. The peptide FDR was set at 0.01, and validation was based on the q value and a decoy database search. All spectra were searched against UniProt-SwissProt proteomes of *Homo sapiens* protein entries appended with contaminants. The reporter ion quantifier node included a TMTpro quantification method with an integration window tolerance of 15 ppm and integration method based on the most confident centroid peak at the MS3 level. Only unique peptides were used for quantification, with protein groups considered for peptide uniqueness. Peptides with an average reporter signal-to-noise ratio of >3 were used for protein quantification. Correction for isotopic impurity of reporter quantification values was applied. Only spectra with at least 50% of the SPS masses matching to the identified peptides were used for quantification.

Normalised protein abundance values for IMiD/CELMoD resistant versus control comparisons were performed using a limma moderated t-test (implemented in Phantasus) [2].

Labelled glucose experiments

Cells were incubated with media containing labelled $^{13}C_6$-glucose (final concentration 2mg/ml) for 24 hours or 48 hours. Quenching solution (60% methanol with 0.85% (w/v) ammonium bicarbonate (pH=7.4)) and extraction solution (100% methanol) were pre-cooled to -40^{0}C. The pre-cooled quenching solution was added to the harvested cell suspension. After centrifugation the cell pellet was re-suspended with extraction solution, and then the extraction process was repeated. The supernatant from each sample was pooled and dried under N_2 flow.

Dual-phase extraction was performed to separate and enrich non-polar metabolites for further analysis. Chloroform (Honeywell)/methanol (Thermo Fisher Scientific) mix (2:1 v/v) and water (Thermo Fisher Scientific) were added to each sample, followed by centrifugation to achieve separation of polar and non-polar phases. The polar phase was collected, and the extraction was repeated to improve extraction efficiency. The polar phase was combined and myristic acid-d27 (Sigma-Aldrich) was added as an internal standard. The samples were then dried under N_2 flow and kept at -80^{0}C.

Next, the samples were derivatized for GC-MS analysis. Specifically, methanol/toluene (Sigma-Aldrich) mix (1:1 v/v) and 0.5M sodium methoxide (Sigma-Aldrich) solution (in methanol) were added to the samples for lipid transmethylation. The samples were vortexed and kept on a Thermomixer (Eppendorf) at 800 RPM for 1 hour at room temperature. The reaction was stopped with 1M sodium chloride

(Sigma-Aldrich) solution (in water) and 37% hydrochloric acid (Sigma-Aldrich) (w/w). Because water reacts with the derivatization reagent, a further extraction step was performed to eliminate water. Hexane (Honeywell) was added and after brief centrifugation to separate polar and non-polar phases, the non-polar phase was collected into a clean vial with magnesium sulfate to achieve better water elimination. The extraction was repeated, and the non-polar phase was pooled and transferred to a clean vial for drying under N_2 flow. The samples were then derivatized by adding acetonitrile (Thermo Fisher Scientific) and MSTFA (N-Methyl-N-trimethylsilyl-trifluoroacetamide) derivatizing reagent (Thermo Fisher Scientific). After thorough vortexing, the samples were placed on the Thermomixer at 800 RPM for 30 minutes at 37^0C. The quality control sample was made by pooling 5µl from each sample.

The order of the samples was randomised before GC-MS analysis. GC-MS analysis was performed on an Agilent 7890 GC coupled to an Agilent 5975 MSD (Agilent Technologies). An Agilent 7693 autosampler was used to inject the samples in splitless mode. Metabolites were then separated on a 30m DB-5MS capillary column using helium as a carrier gas and ionized by election impact ionization. An in-house created compound library and the deconvolution software AMDIS were used for metabolite identification [3]. The MATLAB based algorithm GAVIN was employed to integrate isotopomer peaks and correct for natural abundance of elemental isotopes [4].

Next, Isotopomer Spectral Analysis (ISA) modelling was performed based on the mass isotopomer distribution of fatty acids using a MATLAB based algorithm as described by Tredwell & Keun [5]. Briefly, two parameters to describe fatty acid synthesis were estimated: 1) the proportion of lipogenic acetyl-CoA that is derived from a ^{13}C tracer (parameter D) and 2) the fraction of fatty acids that are *de novo* synthesised during the incubation (g(t)). To verify parameter errors, a Monte Carlo approach based parameter sensitivity analysis was performed.

Supplementary Tables

Supplementary Table 1: Variant allele frequency of each CRBN mutation in the resistant line and its matched control line.

The table shows the variant allele frequency (VAF, mutated allele/total allele) of each mutation in the resistant line and its matched control line.

**Cell line**	**Mutation in CRBN**	**Protein change**	**VAF in the resistant line (%)**	**VAF in the control line (%)**
Pom-R-MM1s	3154076 - C to G	Exon 8 splice site	26.3	3.0
Pom-R-H929	3174138 - G to A	Q100*	80.3	1.1
Iber-R-H929	3174138 - G to A	Q100*	63.6	1.8

Supplementary Table 2: Mutations identified in resistant cell lines.

High impact gene mutations for each of the resistant cell lines (present in resistant line but not its DMSO control line) are shown [6]. Of note the control cell lines also gained some high impact mutations compared to the parental MM1s and H929 cell lines (data not shown), as has previously been observed during long-term cell culture.

**Cell line**	**Mutated genes (with a predicted high impact on the resultant protein)**
Len-R-MM1s	AC004997.1, CASTOR1, DSCAM, FNDC7, KATNBL1, MKI67, PADI3, PLXNA3, SLC38A8, SYNC, WASL, ZNF746
Pom-R-MM1s	ABCA2, AL109761.1, APBA1, ATP6V1F, CD22, CRBN, DGKK, FNDC7, KATNBL1, KIF13B, LAMA3, MKI67, MMP28, PKD1L1, RIPK4, RYR2, SLC4A10, SPPL2B, SPTA1, THBS3, TIGD4, TXNL1, UBR2
Iber-R-MM1s	ABCA2, APBA1, CD22, DSCAM, ELL2, FNDC7, MKI67, MYO1H, PKD1L1, SPTA1, SYNC, THBS3, TXNL1
Len-R-H929	ACADVL, TTN, TXNRD3
Pom-R-H929	ABCB11, ANKRD36, C5, CCNT1, CRBN, DIP2B, IFI16, LAMC3, MAG, MPP1, MTRES1, MYH1, NBEA, SLC4A1AP, TACC2, YAP1, ZBTB41
Iber-R-H929	CCNT1, CRBN, MTRES1

Supplementary Table 3: Common proteins with altered expression in the resistant cell lines.

Table contains the details of 34 proteins with significantly altered expressed in ≥4 of the 6 resistant cell lines compared to their respective controls (Log2 FC ≥0.2 or ≤-0.2, adj p<0.05). Details regarding protein function were adapted from GeneCards [7].

Protein	Full name	Details of protein function	No. of cell lines altered
**Upregulated**			
AMPD1	Adenosine Monophosphate Deaminase 1	Plays a critical role in energy metabolism.	4
CA8	Carbonic Anhydrase 8	Function unclear, does not have carbonic anhydrase catalytic function.	5
CORO1B	Coronin 1B	Regulates cell motility.	
FAM169A	Family With Sequence Similarity 169 Member A	Predicted to be located in the nuclear inner membrane.	6
GPC4	Glypican 4	Cell surface proteoglycan that may play a role in the control of cell division and growth regulation.	4
HMGCR	HMG-CoA Reductase	Catalyses the rate limiting step in cholesterol biosynthesis.	4
MBD3	Methyl-CpG Binding Domain Protein 3	Component of the histone deacetylase NuRD (Nucleosome Remodeling Deacetylase) complex which participates in the remodelling of chromatin.	4
METTL7A	Methyltransferase Like 7A	Predicted to enable methyltransferase activity.	4
MX1	MX Dynamin Like GTPase 1	Encodes a guanosine triphosphate (GTP)-metabolising protein that participates in the cellular antiviral response.	5
ODC1	Ornithine Decarboxylase 1	Catalyses the first (and rate-limiting) step of polyamine biosynthesis.	4
SP100	SP100 Nuclear Antigen	Tumour suppressor that is a major component of PML (promyelocytic leukaemia) bodies which are involved in cell growth, differentiation and apoptosis.	4
SP110	SP110 Nuclear Body Protein	Component of the nuclear body, a multiprotein complex that may have a role in the regulation of gene transcription.	4
SULT1A1	Sulfotransferase Family 1A Member 1	Catalyses the sulfate conjugation of many hormones, neurotransmitters, drugs, and xenobiotic compounds.	4
SULT1A3/ SULT1A4	Sulfotransferase Family 1A Member 3/4	Catalyses the sulfate conjugation of many hormones, neurotransmitters, drugs, and xenobiotic compounds.	4
**Downregulated**			
ACSL4	Acyl-CoA Synthetase Long Chain Family Member 4	Catalyses the conversion of long-chain fatty acids to their active form acyl-CoA, thereby playing a key role in lipid biosynthesis and fatty acid degradation.	4
ADM	Adrenomedullin	Encodes a preprohormone which is cleaved to form two biologically active peptides, adrenomedullin and proadrenomedullin N-terminal 20 peptide.	4
CAV1	Caveolin 1	Main component of caveolae plasma membranes.	

CKMT1B/CKMT1A	Creatine Kinase, Mitochondrial 1B/A	Responsible for the transfer of high energy phosphate from mitochondria to the cytosolic carrier, creatine.	4
CRBN	Cereblon	Substrate receptor of the $CRL4^{CRBN}$ E3 ubiquitin ligase.	6
CRIP1	Cysteine Rich Protein 1	Appears to have a role in zinc absorption and may function as an intracellular zinc transport protein.	4
FASN	Fatty Acid Synthase	Catalyses the synthesis of palmitate from acetyl-CoA and malonyl-CoA.	4
FDPS	Farnesyl Diphosphate Synthase	Key enzyme in isoprenoid biosynthesis which catalyses the formation of farnesyl diphosphate (FPP), a precursor for several classes of essential metabolites including sterols, dolichols, carotenoids, and ubiquinones.	4
HSPB1	Heat Shock Protein Family B (Small) Member 1	Member of the small heat shock protein family of proteins.	4
MAGED1	MAGE Family Member D1	Involved in the apoptotic response after nerve growth factor (NGF) binding in neuronal cells.	4
OAT	Ornithine Aminotransferase	Key enzyme in the pathway that converts arginine and ornithine into the major excitatory and inhibitory neurotransmitters glutamate and GABA.	4
P4HA1	Prolyl 4-Hydroxylase Subunit Alpha 1	Component of prolyl 4-hydroxylase, a key enzyme in collagen synthesis.	5
PAPSS2	3'-Phosphoadenosine 5'-Phosphosulfate Synthase 2	Bifunctional enzyme which mediates two steps in the sulfate activation pathway.	4
PCK2	Phosphoenolpyruvate Carboxykinase 2, Mitochondrial	Mitochondrial enzyme that catalyses the conversion of oxaloacetate to phosphoenolpyruvate.	4
PGRMC1	Progesterone Receptor Membrane Component 1	Putative membrane-associated progesterone steroid receptor.	4
PLOD2	Procollagen-Lysine, 2-Oxoglutarate 5-Dioxygenase 2	Catalyses the hydroxylation of lysyl residues in collagen-like peptides. The resultant hydroxylysyl groups are attachment sites for carbohydrates in collagen and thus are critical for the stability of intermolecular crosslinks.	4
PSPH	Phosphoserine Phosphatase	Catalyses the last irreversible step in the biosynthesis of L-serine from carbohydrates.	4
SCD	Stearoyl-CoA Desaturase	Enzyme involved in fatty acid biosynthesis, primarily the synthesis of oleic acid.	6
SLC2A1	Solute Carrier Family 2 Member 1	Major glucose transporter in the mammalian blood-brain barrier.	4
SUOX	Sulfite Oxidase	Catalyses the oxidation of sulfite to sulfate, the final reaction in the oxidative degradation of the sulfur amino acids cysteine and methionine.	5

Supplementary Table 4: Common proteins with altered expression in the lenalidomide resistant cell lines, pomalidomide resistant cell lines and iberdomide resistant cell lines.

Table contains the details of proteins with significantly altered expressed in both lenalidomide resistant lines, pomalidomide resistant lines and iberdomide resistant lines (each compared to their respective control) (Log2 FC ≥0.2 or ≤-0.2, adj p<0.05). Details regarding protein function were adapted from GeneCards [7].

Protein	Full name	Details of protein function
**Len-R-MM1s and Len-R-H929**		
**Upregulated**		
EPB41L3	Erythrocyte Membrane Protein Band 4.1 Like 3	Predicted to enable cytoskeletal protein-membrane anchor activity.
MGST1	Microsomal Glutathione S-Transferase 1	Catalyses the conjugation of glutathione to electrophiles and the reduction of lipid hydroperoxides.
**Downregulated**		
ACSL4	Acyl-CoA Synthetase Long Chain Family Member 4	Catalyses the conversion of long-chain fatty acids to their active form acyl-CoA, thereby playing a key role in lipid biosynthesis and fatty acid degradation.
CRBN	Cereblon	Substrate recognition component of an E3 protein ligase complex.
ICAM1	Intercellular Adhesion Molecule 1	Cell surface glycoprotein, typically expressed on endothelial and immune system cells.
P4HA1	Prolyl 4-Hydroxylase Subunit Alpha 1	Component of prolyl 4-hydroxylase, a key enzyme in collagen synthesis.
SCD	Stearoyl-CoA Desaturase	Enzyme involved in fatty acid biosynthesis, primarily the synthesis of oleic acid.
SUOX	Sulfite Oxidase	Catalyses the oxidation of sulfite to sulfate, the final reaction in the oxidative degradation of the sulfur amino acids cysteine and methionine.
**Pom-R-MM1s and Pom-R-H929**		
**Upregulated**		
HMGCR	HMG-CoA Reductase	Catalyses the rate limiting step in cholesterol biosynthesis.
ACSS3	Acyl-CoA Synthetase Short Chain Family Member 3	Enables propionate-CoA ligase activity.
ADAM28	ADAM Metallopeptidase Domain 28	Membrane-anchored protein expressed in lymphocytes. Involved in cell-cell and cell-matrix interactions.
AMPD1	Adenosine Monophosphate Deaminase 1	Plays a critical role in energy metabolism.
AMPD3	Adenosine Monophosphate Deaminase 3	Plays a critical role in energy metabolism.
AAMDC	Adipogenesis Associated Mth938 Domain Containing	Predicted to be involved in positive regulation of fat cell differentiation.

CA8	Carbonic Anhydrase 8	Function unclear, does not have carbonic anhydrase catalytic function.
CASP4	Caspase 4	Caspases play a central role in cell apoptosis.
CHEK1	Checkpoint Kinase 1	Required for checkpoint mediated cell cycle arrest in response to DNA damage or the presence of unreplicated DNA.
DCPS	Decapping Enzyme, Scavenger	Catalyses the cleavage of a residual cap structure from degraded mRNA.
DENND3	DENN Domain Containing 3	Enables guanyl-nucleotide exchange factor activity.
DHX58	DExD/H-Box Helicase 58	Has a role in ATP hydrolysis, RNA binding and zinc ion binding.
EVL	Enah/Vasp-Like	Predicted to enable SH3 domain binding activity and profilin binding activity.
ERAP1	Endoplasmic Reticulum Aminopeptidase 1	Plays a central role in peptide trimming, which is required for the generation of most HLA class I-binding peptides.
ETS1	ETS Proto-Oncogene 1, Transcription Factor	Controls the expression of cytokine and chemokine genes. May control the differentiation, survival and proliferation of lymphoid cells
FAM118B	Family With Sequence Similarity 118 Member B	May play a role in Cajal bodies formation.
FAM169A	Family With Sequence Similarity 169 Member A	Predicted to be located in nuclear inner membrane.
FABP6	Fatty Acid Binding Protein 6	Binds to bile acids and is involved in enterohepatic bile acid metabolism.
FGD3	FYVE, RhoGEF And PH Domain Containing 3	Predicted to enable guanyl-nucleotide exchange factor activity and small GTPase binding activity. Predicted to be involved in actin cytoskeleton organisation.
GPC4	Glypican 4	Cell surface proteoglycan that may play a role in the control of cell division and growth regulation.
GRAMD4	GRAM Domain Containing 4	Mediator of E2F1-induced apoptosis in the absence of TP53.
H2AX	H2A Histone Family Member X	Variant histone H2A which replaces conventional H2A in a subset of nucleosomes.
HYOU1	Hypoxia Up-Regulated 1	Role in cytoprotective cellular mechanisms triggered by oxygen deprivation.
ITM2C	Integral Membrane Protein 2C	Enables amyloid-beta binding activity.
ITGAL	Integrin Subunit Alpha L	Receptor for ICAM1, ICAM2, ICAM3 and ICAM4.
IL6ST	Interleukin 6 Signal Transducer	Signal transducer shared by many cytokines, including IL6, CNTF, LIF and OSM.
IQGAP2	IQ Motif Containing GTPase Activating Protein 2	Interacts with components of the cytoskeleton, cell adhesion molecule and several signalling molecules. Also acts as a tumour suppressor and has been found to play a role in regulating innate antiviral responses.
IVD	Isovaleryl-CoA Dehydrogenase	Mitochondrial matrix enzyme that catalyses an intermediate step in leucine catabolism.
KDELC1	KDEL motif containing 1	Catalyses the transfer of glucose from UDP-glucose to a serine residue within the consensus sequence peptide C-X-N-T-X-G-S-F-X-C
LILRB4	Leukocyte Immunoglobulin Like Receptor B4	Inhibitory receptor involved in the downregulation of the immune response and the development of immune tolerance.

LPCAT1	Lysophosphatidylcholine Acyltransferase 1	Plays a role in phospholipid metabolism.
MCC	MCC, WNT Signaling Pathway Regulator	Candidate colorectal tumour suppressor gene that is thought to negatively regulate cell cycle progression.
MBD3	Methyl-CpG Binding Domain Protein 3	Component of the histone deacetylase NuRD (Nucleosome Remodeling Deacetylase) complex which participates in the remodelling of chromatin.
METTL7A	Methyltransferase Like 7A	Predicted to enable methyltransferase activity.
MX1	MX Dynamin Like GTPase 1	Encodes a guanosine triphosphate (GTP)-metabolising protein that participates in the cellular antiviral response.
MYO1F	Myosin IF	Actin-based motor molecule with ATPase activity.
NDUFS4	NADH:Ubiquinone Oxidoreductase Subunit S4	Accessory subunit of the mitochondrial membrane respiratory chain NADH dehydrogenase (Complex I), that is believed not to be involved in catalysis.
NME3	NME/NM23 Nucleoside Diphosphate Kinase 3	Enables nucleoside diphosphate kinase activity.
NUBPL	Nucleotide Binding Protein Like	Iron-sulfur cluster transfer protein involved in the assembly of the mitochondrial membrane respiratory chain NADH dehydrogenase (Complex I).
PLD3	Phospholipase D Family Member 3	Encodes a member of the phospholipase D family of enzymes that catalyse the hydrolysis of membrane phospholipids.
RUFY3	RUN And FYVE Domain Containing 3	Promotes the coupling of endolysosomes to dynein-dynactin for retrograde transport along microtubules.
SP100	SP100 Nuclear Antigen	Tumour suppressor that is a major component of PML (promyelocytic leukaemia) bodies which are involved in cell growth, differentiation and apoptosis.
SP110	SP110 Nuclear Body Protein	Component of the nuclear body, a multiprotein complex that may have a role in the regulation of gene transcription.
SULT1A1	Sulfotransferase Family 1A Member 1	Catalyses the sulfate conjugation of many hormones, neurotransmitters, drugs, and xenobiotic compounds.
SULT1A3/ SULT1A4	Sulfotransferase Family 1A Member 3/4	Catalyses the sulfate conjugation of many hormones, neurotransmitters, drugs, and xenobiotic compounds.
UNC93B1	Unc-93 Homolog B1, TLR Signalling Regulator	Involved in innate and adaptive immune response by regulating toll-like receptor signalling.
VAV1	Vav Guanine Nucleotide Exchange Factor 1	Couples tyrosine kinase signals with the activation of the Rho/Rac GTPases, leading to cell differentiation and/or proliferation.
**Downregulated**		
PAPSS2	3'-Phosphoadenosine 5'-Phosphosulfate Synthase 2	Bifunctional enzyme which mediates two steps in the sulfate activation pathway.
ABHD12	Abhydrolase Domain Containing 12	Mediates the hydrolysis of lysophosphatidylserine, a class of signalling lipids that regulates immunological and neurological processes.
ADM	Adrenomedullin	Encodes a preprohormone which is cleaved to form two biologically active peptides, adrenomedullin and proadrenomedullin N-terminal 20 peptide.

AHNAK	AHNAK nucleoprotein	Encoded a large structural scaffold protein which may play a role in diverse processes such as blood-brain barrier formation, cell structure and migration, cardiac calcium channel regulation, and tumour metastasis.
AHNAK2	AHNAK nucleoprotein 2	Encodes a large nucleoprotein.
AIFM2	Apoptosis Inducing Factor, Mitochondria Associated 2	A NAD(P)H-dependent oxidoreductase that acts as a key inhibitor of ferroptosis
ALOX5	Arachidonate 5-Lipoxygenase	Encodes a member of the lipoxygenase gene family and plays a dual role in the synthesis of leukotrienes from arachidonic acid.
ASNS	Asparagine Synthetase (Glutamine-Hydrolyzing)	Involved in the synthesis of asparagine.
CAV1	Caveolin 1	Main component of caveolae plasma membranes.
CRBN	Cereblon	Substrate recognition component of an E3 protein ligase complex
C12orf57	chromosome 12 open reading frame 57	Required for development of the human corpus callosum.
CPN3	Copine 3	Calcium-dependent membrane-binding protein that may regulate molecular events at the interface of the cell membrane and cytoplasm.
CKMT1B/ CKMT1A	Creatine Kinase, Mitochondrial 1B/Creatine Kinase, Mitochondrial 1A	Responsible for the transfer of high energy phosphate from mitochondria to the cytosolic carrier creatine.
CSRP1	Cysteine And Glycine Rich Protein 1	This gene family includes a group of LIM domain proteins, which may be involved in regulatory processes important for development and cellular differentiation.
COA6	Cytochrome C Oxidase Assembly Factor 6	Involved in the maturation of a mitochondrial respiratory chain complex IV subunit.
DHPS	Deoxyhypusine Synthase	Required for the formation of hypusine, a unique amino acid.
DESI1	Desumoylating Isopeptidase 1	Enables importin-alpha family protein binding activity and long-chain fatty acyl-CoA hydrolase activity.
DESI2	Desumoylating Isopeptidase 2	Enables deubiquitinase activity and long-chain fatty acyl-CoA hydrolase activity.
EML2	Echinoderm Microtubule Associated Protein Like 2	Tubulin binding protein that inhibits microtubule nucleation and growth.
EHHADH	Enoyl-CoA Hydratase And 3-Hydroxyacyl CoA Dehydrogenase	Enzyme involved in the peroxisomal beta-oxidation pathway.
FLNA	Filamin A	Promotes branching of actin filaments and links actin filaments to membrane glycoproteins.
FLNB	Filamin B	Connects cell membrane constituents to the actin cytoskeleton.
GPD2	Glycerol-3-Phosphate Dehydrogenase 2	Calcium-responsive mitochondrial glycerol-3-phosphate dehydrogenase.

GSKIP	GSK3B Interacting Protein	Negative regulator of GSK3-beta in the Wnt signalling pathway.
GCHFR	GTP Cyclohydrolase I Feedback Regulator	Mediates tetrahydrobiopterin inhibition of GTP cyclohydrolase 1.
H1FX	H1 Histone Family Member X	Histones H1 are necessary for the condensation of nucleosome chains into higher-order structures.
HSPA1A/ HSPA1B	Heat Shock Protein Family A (Hsp70) Member 1A/Heat Shock Protein Family A (Hsp70) Member 1B	Molecular chaperone implicated in a wide variety of cellular processes, including protection of the proteome from stress, folding and transport of newly synthesized polypeptides, activation of proteolysis of misfolded proteins and the formation and dissociation of protein complexes.
HSPB1	Heat Shock Protein Family B (Small) Member 1	Member of the small heat shock protein family of proteins.
KHK	Ketohexokinase	Catalyses the phosphorylation of the ketose sugar fructose to fructose-1-phosphate.
NOMO2	NODAL modulator 2	Component of the multi-pass translocon complex that mediates insertion of multi-pass membrane proteins into the lipid bilayer of membranes.
MGMT	O-6-Methylguanine-DNA Methyltransferase	DNA repair protein that is involved in cellular defence against mutagenesis and toxicity from alkylating agents.
OAT	Ornithine Aminotransferase	Key enzyme in the pathway that converts arginine and ornithine into the major excitatory and inhibitory neurotransmitters glutamate and GABA.
PHGDH	Phosphoglycerate Dehydrogenase	Catalyses the first step of the phosphorylated L-serine biosynthesis pathway.
PSPH	Phosphoserine Phosphatase	Catalyses the last irreversible step in the biosynthesis of L-serine from carbohydrates.
PGRMC1	Progesterone Receptor Membrane Component 1	Putative membrane-associated progesterone steroid receptor.
PACSIN1	Protein Kinase C And Casein Kinase Substrate In Neurons 1	Enables phospholipid binding activity.
RAB11FIP 1	RAB11 Family Interacting Protein 1	Involved in the endosomal recycling process.
SLC2A1	Solute Carrier Family 2 Member 1	Major glucose transporter in the mammalian blood-brain barrier.
SLC6A9	Solute Carrier Family 6 Member 9	Transporter that stops glycine signalling by removing it from the synaptic cleft.
SCD	Stearoyl-CoA Desaturase	Enzyme involved in fatty acid biosynthesis, primarily the synthesis of oleic acid.
TXNL1	Thioredoxin Like 1	Enables disulfide oxidoreductase activity.
TJP1	Tight Junction Protein 1	Acts as a tight junction adaptor protein that also regulates adherens junctions.
TGOLN2	Trans-Golgi Network Protein 2	May be involved in regulating membrane traffic to and from trans-Golgi network.
ZYX	Zyxin	Zinc-binding phosphoprotein that concentrates at focal adhesions and along the actin cytoskeleton.

**Iber-R-MM1s and Iber-R-H929**		
**Upregulated**		
AGMAT	Agmatinase	Hydrolyzes linear guanidino acids to form urea and the corresponding amines.
CA8	Carbonic Anhydrase 8	Function unclear, does not have carbonic anhydrase catalytic function.
CPXM1	Carboxypeptidase X, M14 Family Member 1	May be involved in cell-cell interactions.
CORO1B	Coronin 1B	Regulates leading edge dynamics and cell motility in fibroblasts.
FAM169A	Family With Sequence Similarity 169 Member A	Predicted to be located in nuclear inner membrane.
FNBP1	Formin Binding Protein 1	May act as a link between RND2 signaling and regulation of the actin cytoskeleton.
GALM	Galactose Mutarotase	Catalyses the interconversion of beta-D-galactose and alpha-D-galactose during galactose metabolism.
GALT	Galactose-1-Phosphate Uridylyltransferase	Involved in galactose metabolism.
GPX4	Glutathione Peroxidase 4	Essential antioxidant peroxidase.
GBP2	guanylate binding protein 2	GTPase which hydrolyses GTP, predominantly to GDP.
LY75/LY75-CD302	Lymphocyte Antigen 75/LY75-CD302 Readthrough	Predicted to be involved in immune response and inflammatory response.
METTL7A	Methyltransferase Like 7A	Predicted to enable methyltransferase activity.
MAPT	microtubule associated protein tau	Promotes microtubule assembly and stability.
MX1	MX Dynamin Like GTPase 1	Encodes a guanosine triphosphate (GTP)-metabolising protein that participates in the cellular antiviral response.
MYEF2	Myelin Expression Factor 2	Transcriptional repressor of the myelin basic protein gene.
ODC1	Ornithine Decarboxylase 1	Catalyses the first (and rate-limiting) step of polyamine biosynthesis.
PNPLA4	Patatin Like Phospholipase Domain Containing 4	Has both triacylglycerol lipase and transacylase activities.
PRKCB	Protein Kinase C Beta	PKC family members phosphorylate a wide variety of protein targets and are known to be involved in diverse cellular signalling pathways.
ARHGAP6	Rho GTPase Activating Protein 6	Thought to have two independent functions; GTPase-activating protein with specificity for RhoA and cytoskeletal protein that promotes actin remodelling
SELENOH	Selenoprotein H	May be involved in a redox-related process.
SH3GL2	SH3 Domain Containing GRB2 Like 2, Endophilin A1	Implicated in synaptic vesicle endocytosis.
SNX18	Sorting Nexin 18	Involved in endocytosis and intracellular vesicle trafficking, both during interphase and at the end of mitosis.

VAV2	Vav Guanine Nucleotide Exchange Factor 2	Guanine nucleotide exchange factor for the Rho family of Ras-related GTPases.
**Downregulated**		
ADM	Adrenomedullin	Encodes a preprohormone which is cleaved to form two biologically active peptides, adrenomedullin and proadrenomedullin N-terminal 20 peptide.
ALDH1L2	Aldehyde Dehydrogenase 1 Family Member L2	Catalyses the NADP(+)-dependent conversion of 10-formyltetrahydrofolate to tetrahydrofolate and carbon dioxide.
CAV1	Caveolin 1	Main component of caveolae plasma membranes.
CRBN	Cereblon	Substrate recognition component of an E3 protein ligase complex
CRIP1	Cysteine Rich Protein 1	Appears to have a role in zinc absorption and may function as an intracellular zinc transport protein.
ENAH	ENAH, Actin Regulator	Actin-associated protein involved in a range of processes dependent on cytoskeleton remodelling and cell polarity
FAM136A	Family With Sequence Similarity 136 Member A	Encodes a mitochondrially localised protein that is highly conserved across species.
FDPS	Farnesyl Diphosphate Synthase	Key enzyme in isoprenoid biosynthesis which catalyses the formation of farnesyl diphosphate (FPP), a precursor for several classes of essential metabolites including sterols, dolichols, carotenoids, and ubiquinones.
FASN	Fatty Acid Synthase	Catalyses the synthesis of palmitate from acetyl-CoA and malonyl-CoA.
GNG7	G Protein Subunit Gamma 7	predicted to be involved in G protein-coupled receptor signalling pathway and regulation of adenylate cyclase activity.
GCFC2	GC-Rich Sequence DNA-Binding Factor 2	Involved in pre-mRNA splicing
GHITM	Growth Hormone Inducible Transmembrane Protein	Role in maintenance of mitochondrial morphology and in mediating either calcium or potassium/proton antiport.
HSPB1	Heat Shock Protein Family B (Small) Member 1	Member of the small heat shock protein family of proteins.
IDI1	Isopentenyl-Diphosphate Delta Isomerase 1	Converts isopentenyl diphosphate (IPP) into its more reactive form, dimethylallyl diphosphate (DMAPP), which are then used to produce farnesyl diphosphate and ultimately cholesterol.
MAGED1	MAGE Family Member D1	Involved in the apoptotic response after nerve growth factor (NGF) binding in neuronal cells.
OAT	Ornithine Aminotransferase	Key enzyme in the pathway that converts arginine and ornithine into the major excitatory and inhibitory neurotransmitters glutamate and GABA.
PCK2	Phosphoenolpyruvate Carboxykinase 2, Mitochondrial	Mitochondrial enzyme that catalyses the conversion of oxaloacetate to phosphoenolpyruvate.
PSPH	Phosphoserine Phosphatase	Catalyses the last irreversible step in the biosynthesis of L-serine from carbohydrates.
PLS1	Plastin 1	Actin-bundling protein.
PLOD2	Procollagen-Lysine,2-Oxoglutarate 5-Dioxygenase 2	Catalyses the hydroxylation of lysyl residues in collagen-like peptides.

P4HA1	Prolyl 4-Hydroxylase Subunit Alpha 1	Component of prolyl 4-hydroxylase, a key enzyme in collagen synthesis.
SCD	Stearoyl-CoA Desaturase	Enzyme involved in fatty acid biosynthesis, primarily the synthesis of oleic acid.
SARM1	Sterile Alpha And TIR Motif Containing 1	NAD(+) hydrolase, which plays a key role in axonal degeneration following injury by regulating NAD(+) metabolism.
SUOX	Sulfite Oxidase	Catalyses the oxidation of sulfite to sulfate, the final reaction in the oxidative degradation of the sulfur amino acids cysteine and methionine.
SYNM	Synemin	Intermediate filament family member.

Supplementary Table 5: Proteome changes in CRBN-KO-MM1s compared to parental MM1s.

Quantitative proteomic data was analysed by a limma modified t-test in Phantasus (v1.19.3). The top 10 most downregulated and upregulated proteins in the CRBN-KO-MM1s (adj $p<0.05$) are described. Descriptions adapted from GeneCards [7].

Protein	Full name	Description	Log2FC
**Upregulated proteins in CRBN-KO-MM1s**			
MGST1	Microsomal Glutathione S-Transferase 1	Catalyses the conjugation of glutathione to electrophiles and the reduction of lipid hydroperoxides	1.91
TNFSF9	TNF Superfamily Member 9	Cytokine that belongs to the tumour necrosis factor ligand family	1.90
MYO1F	Myosin IF	Actin-based motor molecule with ATPase activity	1.81
ADGRE5	Adhesion G Protein-Coupled Receptor E5	Receptor potentially involved in adhesion and signalling processes early after leukocyte activation	1.72
RASGRP3	RAS Guanyl Releasing Protein 3	Guanine nucleotide exchange factor for Ras and Rap1	1.64
GSN	Gelsolin	Calcium-regulated, actin-modulating protein	1.51
IL4I1	Interleukin 4 Induced 1	Secreted L-amino-acid oxidase that acts as a key immunoregulator	1.45
TLR7	Toll Like Receptor 7	Member of the Toll-like receptor family	1.44
UBA7	Ubiquitin Like Modifier Activating Enzyme 7	Activates ubiquitin	1.41
PTPRS	Protein Tyrosine Phosphatase Receptor Type S	Cell surface receptor that binds to glycosaminoglycans	1.38
**Downregulated proteins in CRBN-KO-MM1s**			
GCFC2	GC-Rich Sequence DNA-Binding Factor 2	Involved in pre-mRNA splicing	-3.47
GYPC	Glycophorin C (Gerbich Blood Group)	Integral membrane glycoprotein	-3.29
SULT1A3/ SULT1A4	Sulfotransferase Family 1A Member 3/4	Sulfotransferase enzymes	-2.85
CMBL	Carboxymethylenebutenolidase Homolog	Cysteine hydrolase	-2.47
TP53I11	Tumor Protein P53 Inducible Protein 11	Predicted to be involved in negative regulation of proliferation	-2.44
CRBN	Cereblon	Substrate recognition component of an E3 protein ligase complex	-2.24
PFKP	Phosphofructokinase, Platelet	Member of the phosphofructokinase A protein family	-1.98
C1orf198	Chromosome 1 Open Reading Frame 198	Unknown function	-1.70
BRSK1	BR Serine/Threonine Kinase 1	Serine/threonine-protein kinase that plays a key role in polarisation of neurons and centrosome duplication	-1.64
ABLIM3	Actin Binding LIM Protein Family Member 3	Member of the actin-binding LIM family of proteins	-1.59

Supplementary Table 6: Common transcriptome changes in the resistant cell lines

Twenty-seven transcripts were commonly changed in ≥5 of the 6 resistant cell lines compared to their respective controls (Log2 FC ≥0.2 or ≤-0.2, adj p<0.05). Descriptions adapted from GeneCards [7].

Gene symbol	Full name	Description of protein function
**Upregulated**		
BIRC3	Baculoviral IAP Repeat Containing 3	Member of the IAP (Inhibitor of Apoptosis) family of proteins that inhibit apoptosis.
CA8	Carbonic Anhydrase 8	Function unclear, does not have carbonic anhydrase catalytic function.
CBLN2	Cerebellin 2 Precursor	Predicted to be involved in the maintenance of synapse structure and spontaneous synaptic transmission.
DOCK4	Dedicator Of Cytokinesis 4	Functions as a guanine nucleotide exchange factor and is involved in regulation of adherens junctions between cells.
FAM169A	Family With Sequence Similarity 169 Member A	Function unknown.
FNBP1	Formin Binding Protein 1	Member of the formin-binding protein family.
KAT6B	Lysine Acetyltransferase 6B	Histone acetyltransferase which may be involved in both positive and negative regulation of transcription.
KCNC4	Potassium Voltage-Gated Channel Subfamily C Member 4	Mediates the voltage-dependent potassium ion permeability of excitable membranes.
KLF10	KLF Transcription Factor 10	Member of a family of proteins that have C2H2-type zinc finger domains. Involved in the regulation of transforming growth factor beta (TGFβ) signalling.
MEST	Mesoderm Specific Transcript	Encodes a member of the alpha/beta hydrolase superfamily. May play a role in development.
METTL7A	Methyltransferase Like 7A	Predicted to enable methyltransferase activity.
NEGR1	Neuronal Growth Regulator 1	Function unclear, may be involved in cell adhesion.
NFIX	Nuclear Factor I X	Transcription factor that binds to the palindromic sequence 5'-TTGGCNNNNNGCCAA-3 in viral and cellular promoters.
PLK2	Polo Like Kinase 2	Member of the polo family of serine/threonine protein kinases that have a role in cell division.
ST8SIA2	ST8 Alpha-N-Acetyl-Neuraminide Alpha-2,8-Sialyltransferase 2	Type II membrane protein that catalyses the transfer of sialic acid from CMP-sialic acid to N-linked oligosaccharides and glycoproteins.
SULT1A3	Sulfotransferase Family 1A Member 3	Catalyses the sulfate conjugation of many hormones, neurotransmitters, drugs, and xenobiotic compounds.
TNFAIP3	TNF Alpha Induced Protein 3	Inhibits NFκB activation and TNF mediated apoptosis and has ubiquitin ligase and deubiquitinase activities.
**Downregulated**		
ANXA9	Annexin A9	Member of a family of calcium-dependent phospholipid-binding proteins.

BBS7	Bardet-Biedl Syndrome 7	Thought to be required for the sorting of specific membrane proteins to primary cilia.
C2	Complement C2	Serum glycoprotein that functions as part of the classical pathway of the complement system.
CMTM7	CKLF Like MARVEL Transmembrane Domain Containing 7	Tumour suppressor that regulates G1/S transition in the cell cycle and influences epidermal growth factor receptor/protein kinase B signalling during tumour pathogenesis.
CRBN	Cereblon	Substrate receptor of the $CRL4^{CRBN}$ E3 ubiquitin ligase.
LINC00665	Long Intergenic Non-Protein Coding RNA 665	RNA Gene, affiliated with the long non-coding RNA class.
MED8	Mediator Complex Subunit 8	Component of the Mediator complex, which is involved in the regulated transcription of nearly all RNA polymerase II-dependent genes.
POGLUT3	Protein O-Glucosyltransferase 3	Protein glucosyltransferase that may be involved in regulation of the NOTCH signalling pathway.
RPS6KA2	Ribosomal Protein S6 Kinase A2	Serine/threonine-protein kinase that acts downstream of ERK (Extracellular Signal Regulated Kinases) signalling.
TBC1D9	TBC1 Domain Family Member 9	Predicted to be involved in activation of GTPase activity and intracellular protein transport.

Supplementary Table 7: Over-representation analysis of transcripts with altered expression in the resistant cell lines.

Over-representation analysis was performed on upregulated and downregulated transcripts in the resistant cell lines. Analysis was performed on statistically significantly altered transcripts (adj p<0.05), with the top 100 transcripts by fold change analysed if the set size was >100. Analysis performed using WebGestalt [8].

**Cell line**	**Number of upregulated transcripts in the resistant line** (adj p<0.05)	**Enriched pathways in upregulated transcripts** (FDR ≤0.05)
Len-R-MM1s	55	N/A
Pom-R-MM1s	1147	N/A
Iber-R-MM1s	259	N/A
Len-R-H929	2236	N/A
Pom-R-H929	4230	N/A
Iber-R-H929	4264	N/A
**Cell line**	**Number of downregulated transcripts in the resistant line** (adj p<0.05)	**Enriched pathways in downregulated transcripts**
Len-R-MM1s	33	N/A
Pom-R-MM1s	1692	N/A
Iber-R-MM1s	370	Biosynthesis of amino acids
Len-R-H929	2466	N/A
Pom-R-H929	4507	N/A
Iber-R-H929	3991	N/A

Supplementary Table 8: Patient characteristics of samples from the Myeloma XI trial

The characteristics of the four patients who had available bone marrow samples for analysis are described here. Adverse cytogenetic disease was defined as the presence of ≥1 of t(4;14), t(14;16), t(14;20), 1q+ and 17p-. CTD, cyclophosphamide, thalidomide, dexamethasone; CRD, cyclophosphamide, lenalidomide, dexamethasone; CRDa, attenuated CRD; ISS, international staging system; VGPR, very good partial response; PR, partial response.

	**Patient ID**			
	A	B	C	D
**Sex**	Male	Female	Male	Male
**Induction therapy**	CRDa	CTD	CRD	CRD
**Underwent autologous stem cell transplant**	No	Yes	Yes	Yes
**ISS stage**	2	Unknown	3	2
**Paraprotein class**	IgG	IgA	IgG	IgG
**Light chain type**	Lambda	Kappa	Lambda	Kappa
**Presence of adverse cytogenetic lesion**	No	Yes	No	No
**Response before lenalidomide maintenance commenced**	VGPR	VGPR	VGPR	PR

Supplementary Table 9: Gene effect scores in Iber-R-MM1s (experimental data) and parental MM1s (DepMap)

The table describes the 47 genes that may represent novel vulnerabilities in the IMiD/CELMoD resistant state. Descriptions adapted from GeneCards [7].

Gene symbol	Gene name	Gene effect in Iber-R-MM1s	Gene effect in parental MM1s	Description
MRPL52	Mitochondrial Ribosomal Protein L52	-1.66	-0.10	Component of the mitochondrial large ribosomal subunit.
LIPT2	Lipoyl(Octanoyl) Transferase 2	-1.42	0.04	Involved in essential redox reactions.
MRPL20	Mitochondrial Ribosomal Protein L20	-1.65	-0.33	Component of the mitochondrial large ribosomal subunit.
PTPMT1	Protein Tyrosine Phosphatase Mitochondrial 1	-1.72	-0.45	Lipid phosphatase.
PRMT1	Protein Arginine Methyltransferase 1	-1.47	-0.29	Arginine methyltransferase.
NSMCE4A	NSE4 Homolog A, SMC5-SMC6 Complex Component	-1.28	-0.11	Component of the SMC5-SMC6 complex.
HSD17B10	Hydroxysteroid 17-Beta Dehydrogenase 10	-1.53	-0.37	Mitochondrial dehydrogenase involved in pathways of fatty acid, branched-chain amino acid and steroid metabolism.
RNASEK	Ribonuclease K	-1.23	-0.08	Endoribonuclease.
VARS2	Valyl-TRNA Synthetase 2, Mitochondrial	-1.46	-0.32	Mitochondrial aminoacyl-tRNA synthetase.
GART	Phosphoribosylglycinamide Formyltransferase	-1.48	-0.35	Involved in de novo purine synthesis.
LIN52	Lin-52 DREAM MuvB Core Complex Component	-1.52	-0.40	Exists in quiescent cells where it represses cell cycle-dependent genes.
DHPS	Deoxyhypusine Synthase	-1.34	-0.23	Required for the formation of hypusine, a unique amino acid.
MBTPS1	Membrane Bound Transcription Factor Peptidase, Site 1	-1.41	-0.32	Serine protease, known substrates are SREBP1, SREBP2, BDNF, GNPTAB, ATF6 and ATF6B.
INO80	INO80 Complex ATPase Subunit	-1.58	-0.49	ATPase component of the INO80 chromatin remodeling complex.
HDAC3	Histone Deacetylase 3	-1.50	-0.49	Responsible for the deacetylation of lysine residues on core histones.
METTL3	Methyltransferase 3, N6-Adenosine-Methyltransferase Complex Catalytic Subunit	-1.24	-0.24	Methylates adenosine residues of some RNAs and regulates various processes including the circadian clock, differentiation of embryonic and hematopoietic stem cells, cortical neurogenesis, response to

				DNA damage, differentiation of T-cells and primary miRNA processing.
MRPL48	Mitochondrial Ribosomal Protein L48	-1.31	-0.33	Component of the mitochondrial large ribosomal subunit.
COG4	Component Of Oligomeric Golgi Complex 4	-1.33	-0.37	Plays a role in SNARE-pin assembly and Golgi-to-ER retrograde transport.
ATIC	5-Aminoimidazole-4-Carboxamide Ribonucleotide Formyltransferase/IMP Cyclohydrolase	-1.25	-0.33	Catalyses the last two steps of purine biosynthesis.
MEAF6	MYST/Esa1 Associated Factor 6	-1.31	-0.45	Component of the NuA4 histone acetyltransferase complex which is involved in transcriptional activation of select genes.
TMED10	Transmembrane P24 Trafficking Protein 10	-1.22	-0.36	Cargo receptor involved in protein vesicular trafficking and quality control in the endoplasmic reticulum and Golgi.
ISCA1	Iron-Sulfur Cluster Assembly 1	-1.15	-0.30	Involved in the maturation of mitochondrial 4Fe-4S proteins.
MTHFD1	Methylenetetrahydrofolate Dehydrogenase, Cyclohydrolase And Formyltetrahydrofolate Synthetase 1	-1.28	-0.46	Involved in the pathway of tetrahydrofolate interconversion, which is part of one-carbon metabolism.
TUBGCP2	Tubulin Gamma Complex Component 2	-1.21	-0.42	Enables gamma-tubulin binding activity.
SEL1L	SEL1L Adaptor Subunit Of SYVN1 Ubiquitin Ligase	-1.16	-0.36	Required for the retro-translocation of misfolded proteins from the endoplasmic reticulum lumen to the cytosol, where they are degraded by the proteasome in a ubiquitin-dependent manner.
C19orf25	Chromosome 19 Open Reading Frame 25	-1.07	-0.28	Unknown function.
MED12	Mediator Complex Subunit 12	-1.25	-0.48	Component of the Mediator Complex, a coactivator involved in the regulated transcription of nearly all RNA polymerase II-dependent genes.
UQCRB	Ubiquinol-Cytochrome C Reductase Binding Protein	-1.15	-0.38	Part of the mitochondrial electron transport chain which drives oxidative phosphorylation.
ITPK1	Inositol-Tetrakisphosphate 1-Kinase	-1.16	-0.39	Kinase that can phosphorylate various inositol polyphosphates.

ZBTB8OS	Zinc Finger And BTB Domain Containing 8 Opposite Strand	-1.05	-0.32	Component of the tRNA-splicing ligase complex.
VPS52	VPS52 Subunit Of GARP Complex	-1.22	-0.49	Component of the GARP complex that is involved in retrograde transport from early and late endosomes to the trans-Golgi network.
HNRNPA1	Heterogeneous Nuclear Ribonucleoprotein A1	-1.14	-0.42	Involved in the packaging of pre-mRNA into hnRNP particles, transport of poly(A) mRNA from the nucleus to the cytoplasm and may modulate splice site selection.
NDUFA1	NADH:Ubiquinone Oxidoreductase Subunit A1	-1.18	-0.46	Accessory subunit of the mitochondrial membrane respiratory chain NADH dehydrogenase (Complex I).
ARMC5	Armadillo Repeat Containing 5	-1.19	-0.47	Involved in foetal development, T-cell function, and adrenal gland growth homeostasis.
SETD2	SET Domain Containing 2, Histone Lysine Methyltransferase	-1.15	-0.43	Histone methyltransferase that specifically trimethylates Lys-36 of histone H3.
FPGS	Folylpolyglutamate Synthase	-1.19	-0.49	Catalyses conversion of folates to polyglutamate derivatives, allowing concentration of folate compounds in the cell and the intracellular retention of these cofactors. These are important substrates for folate-dependent enzymes that are involved in one-carbon transfer reactions (required for purine, pyrimidine, and amino acid synthesis.)
DHX9	DExH-Box Helicase 9	-1.10	-0.41	Multifunctional ATP-dependent nucleic acid helicase that unwinds DNA and RNA in a 3' to 5' direction.
GADD45GIP1	GADD45G Interacting Protein 1	-1.01	-0.34	Acts as a negative regulator of G1 to S cell cycle phase progression by inhibiting cyclin-dependent kinases.
IRF2	Interferon Regulatory Factor 2	-1.05	-0.40	Specifically binds to the upstream regulatory region of type I IFN and IFN-inducible MHC class I genes and represses those genes.
RMI1	RecQ Mediated Genome Instability 1	-1.08	-0.44	Component of the RMI complex, which is involved in the processing of homologous recombination intermediates to limit DNA crossover formation in cells.
NDUFB11	NADH:Ubiquinone Oxidoreductase Subunit B11	-1.09	-0.47	Accessory subunit of the mitochondrial membrane respiratory chain NADH dehydrogenase (Complex I).
PFN1	Profilin 1	-1.04	-0.42	Binds to actin and affects the structure of the cytoskeleton.

SCD	Stearoyl-CoA Desaturase	-1.07	-0.47	Stearoyl-CoA desaturase that introduces the first double bond into saturated fatty acyl-CoA substrates.
KRT18	Keratin 18	-1.07	-0.47	Involved in the uptake of thrombin-antithrombin complexes by hepatic cells.
RPIA	Ribose 5-Phosphate Isomerase A	-1.01	-0.41	Involved in a sub pathway of the pentose phosphate pathway, which is itself part of carbohydrate degradation.
TRMT5	TRNA Methyltransferase 5	-1.07	-0.48	Involved in mitochondrial tRNA methylation.
PFDN5	Prefoldin Subunit 5	-1.05	-0.50	Binds specifically to cytosolic chaperonin and transfers target proteins to it. Represses the transcriptional activity of MYC.

Supplementary Figures

Supplementary Figure 1: Viability assays demonstrating response of resistant lines A) MM1s and B) H929 to other CRBN binding agents

Effect of Len, Pom, Iber and Mezi on the viability of control and resistant (A) MM1s and (B) H929 cell lines as assessed by CellTiter-Blue assay after 5 days of incubation. Mean and SEM shown, n=3.

A)

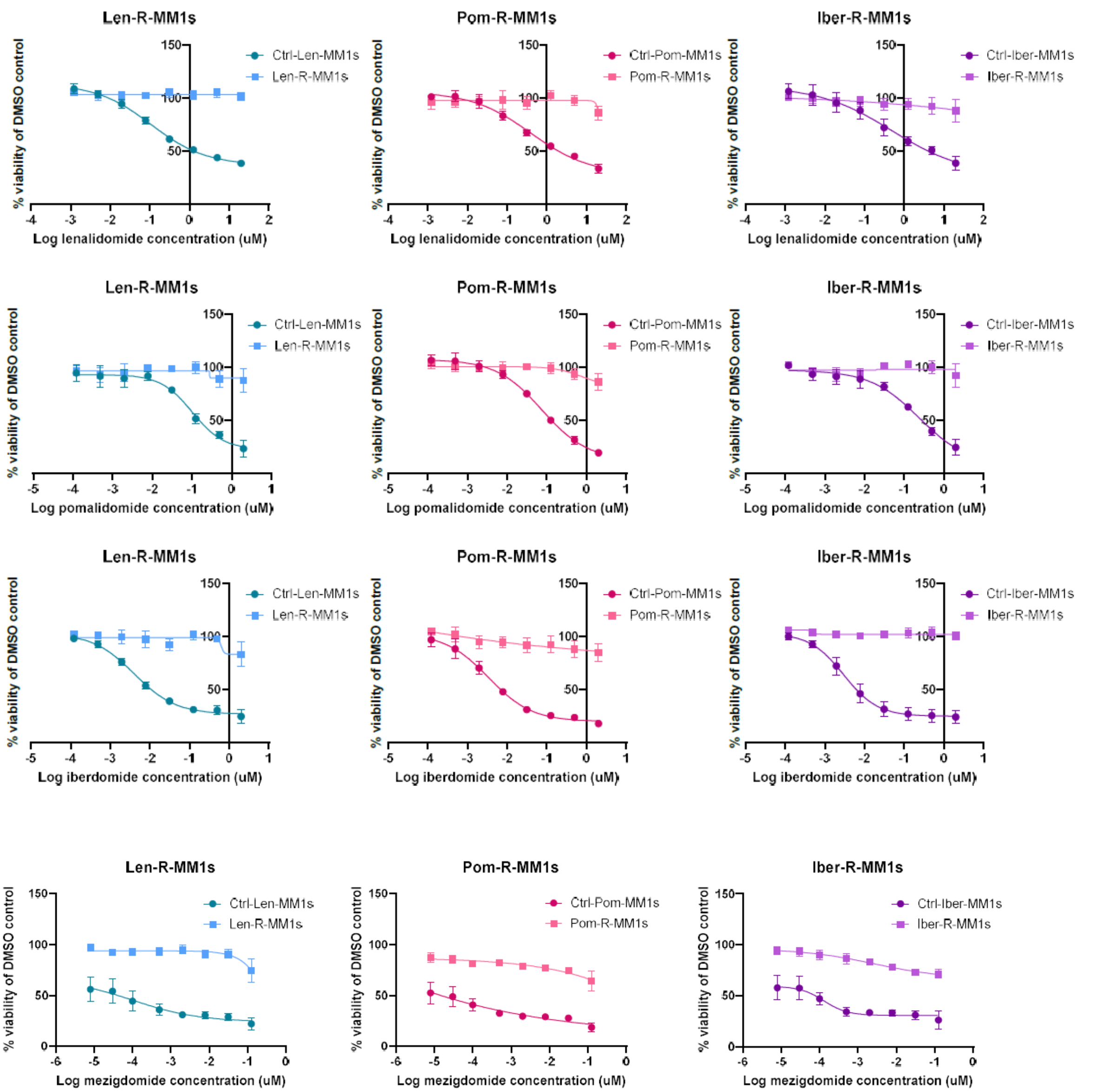

B)

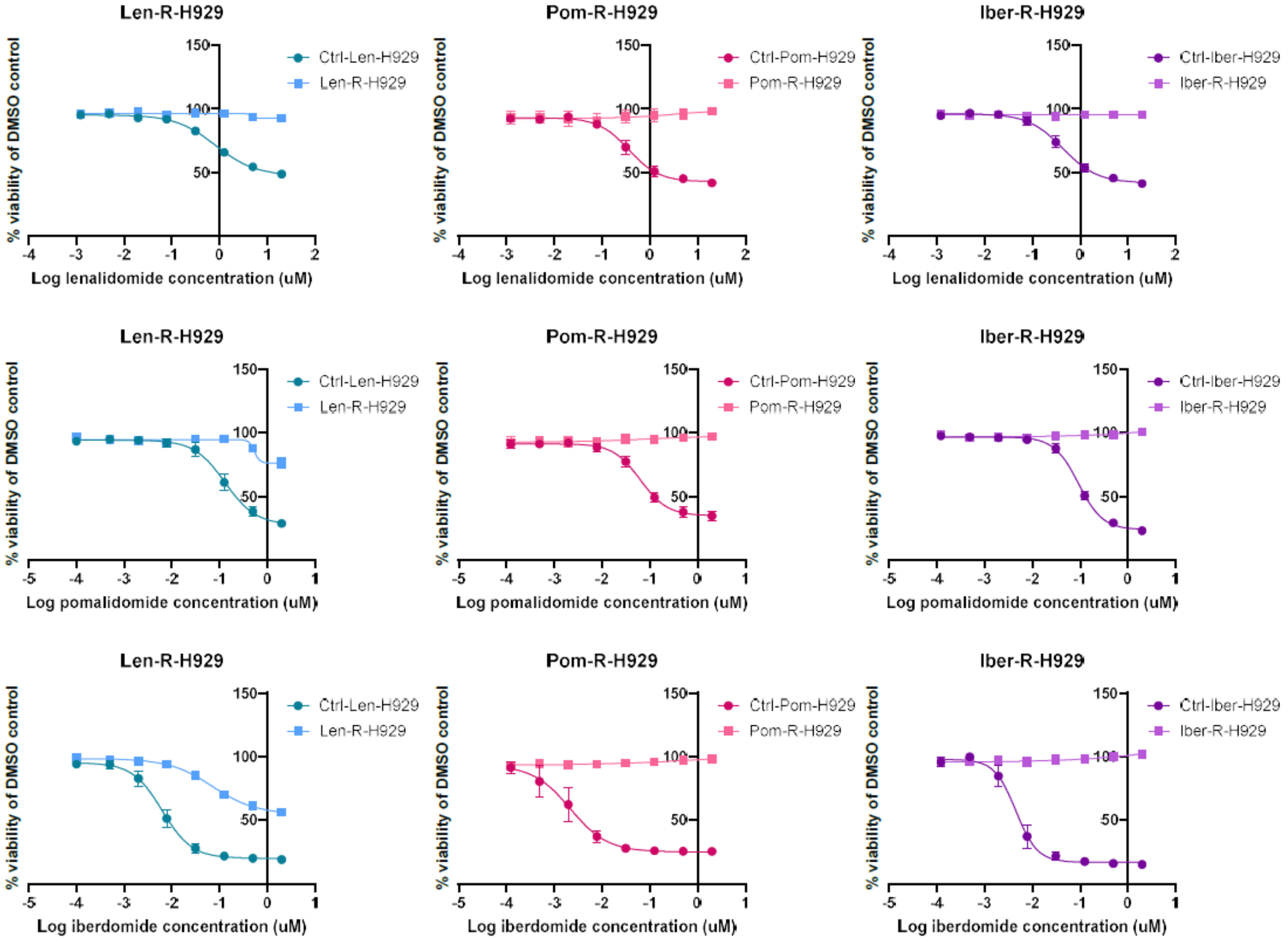
Len-R-H929
Pom-R-H929
Iber-R-H929
Ctrl-Len-H929
Len-R-H929
Ctrl-Pom-H929
Pom-R-H929
Ctrl-Iber-H929
Iber-R-H929
% viability of DMSO control
Log lenalidomide concentration (uM)
Log pomalidomide concentration (uM)
Log iberdomide concentration (uM)

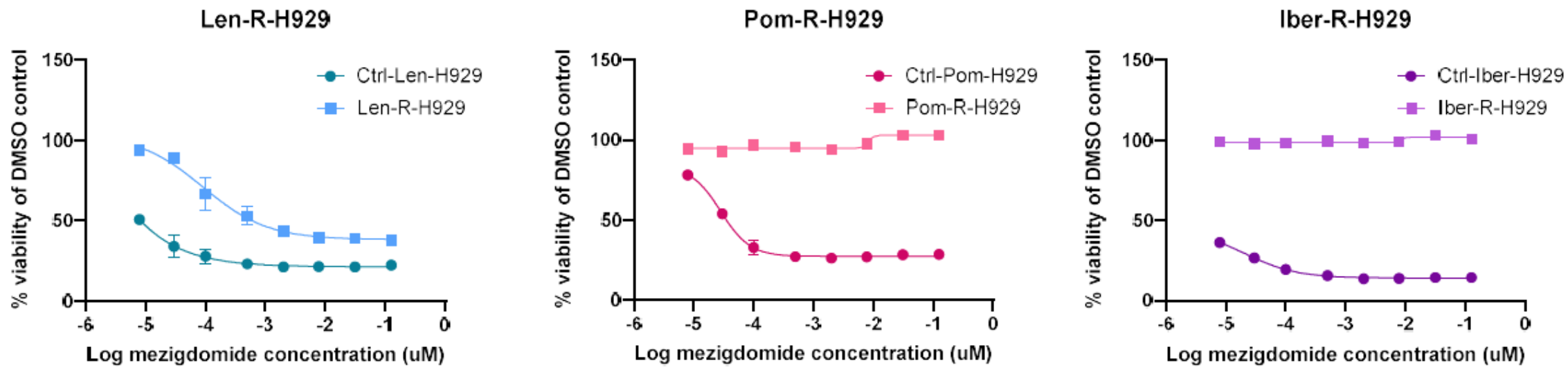
Len-R-H929
Pom-R-H929
Iber-R-H929
Ctrl-Len-H929
Len-R-H929
Ctrl-Pom-H929
Pom-R-H929
Ctrl-Iber-H929
Iber-R-H929
% viability of DMSO control
Log mezigdomide concentration (uM)

Supplementary Figure 2: Viability assays demonstrating response of resistant line Iber-R-MM1s to other anti-myeloma agents

In a 5 day cell CellTiter-Blue viability assay bortezomib, doxorubicin, panobinostat, KPT-330/selinexor and MLN9708/ixazomib reduced viability equally in the Ctrl-Iber-MM1s and Iber-R-MM1s cell lines at low concentrations. Mean and SEM shown, n=3.

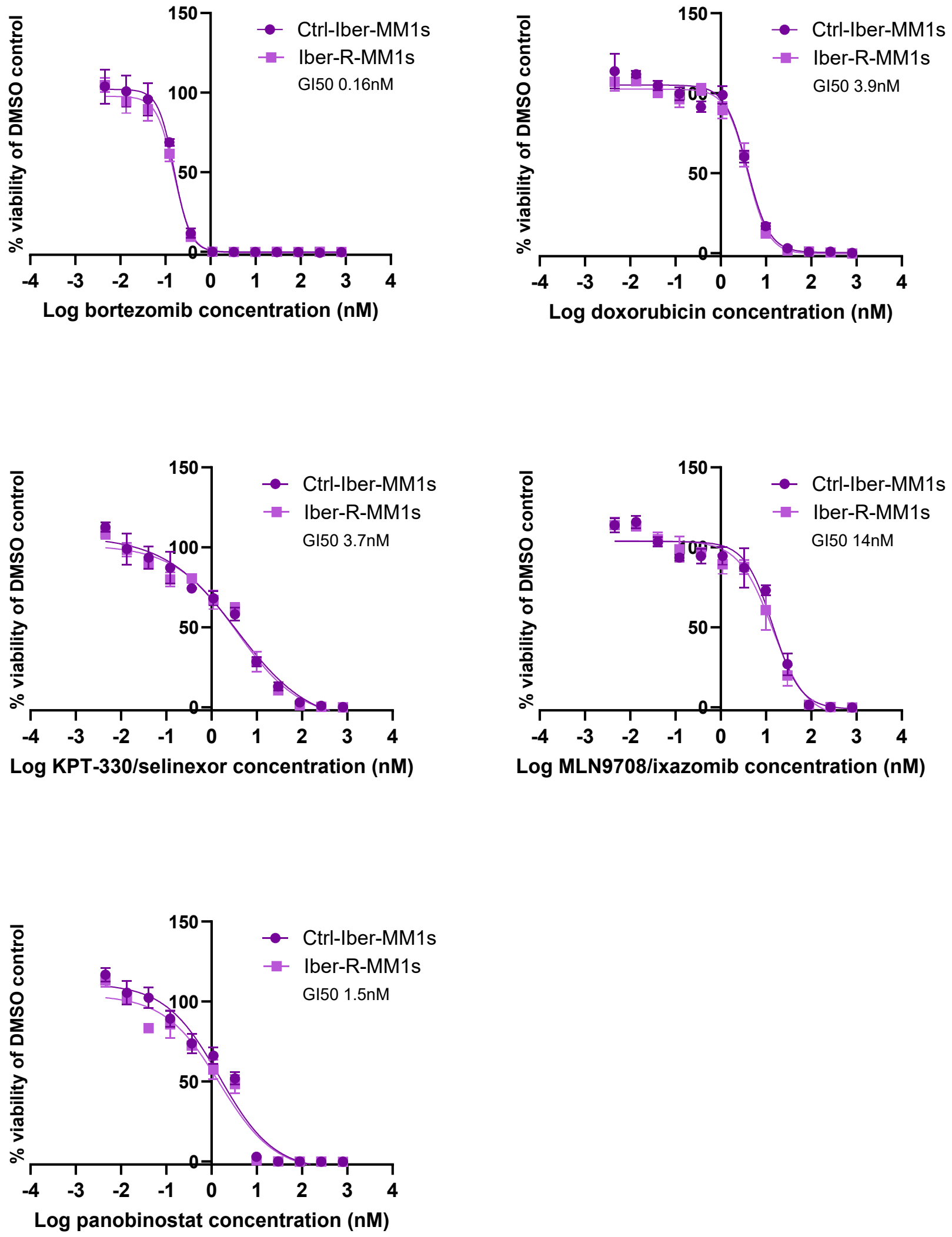

Supplementary Figure 3: Time course of resistance acquisition in A) MM1s and B) H929

Plots show results of cell viability assays (CellTiter-Blue) in A) MM1s and B) H929 after 5 days incubation with IMiD/CELMoD at the indicated week after commencing exposure to Len/Pom/Iber. Results for each cell line are normalised to DMSO treatment control. Mean and SEM shown, n=3.

A)

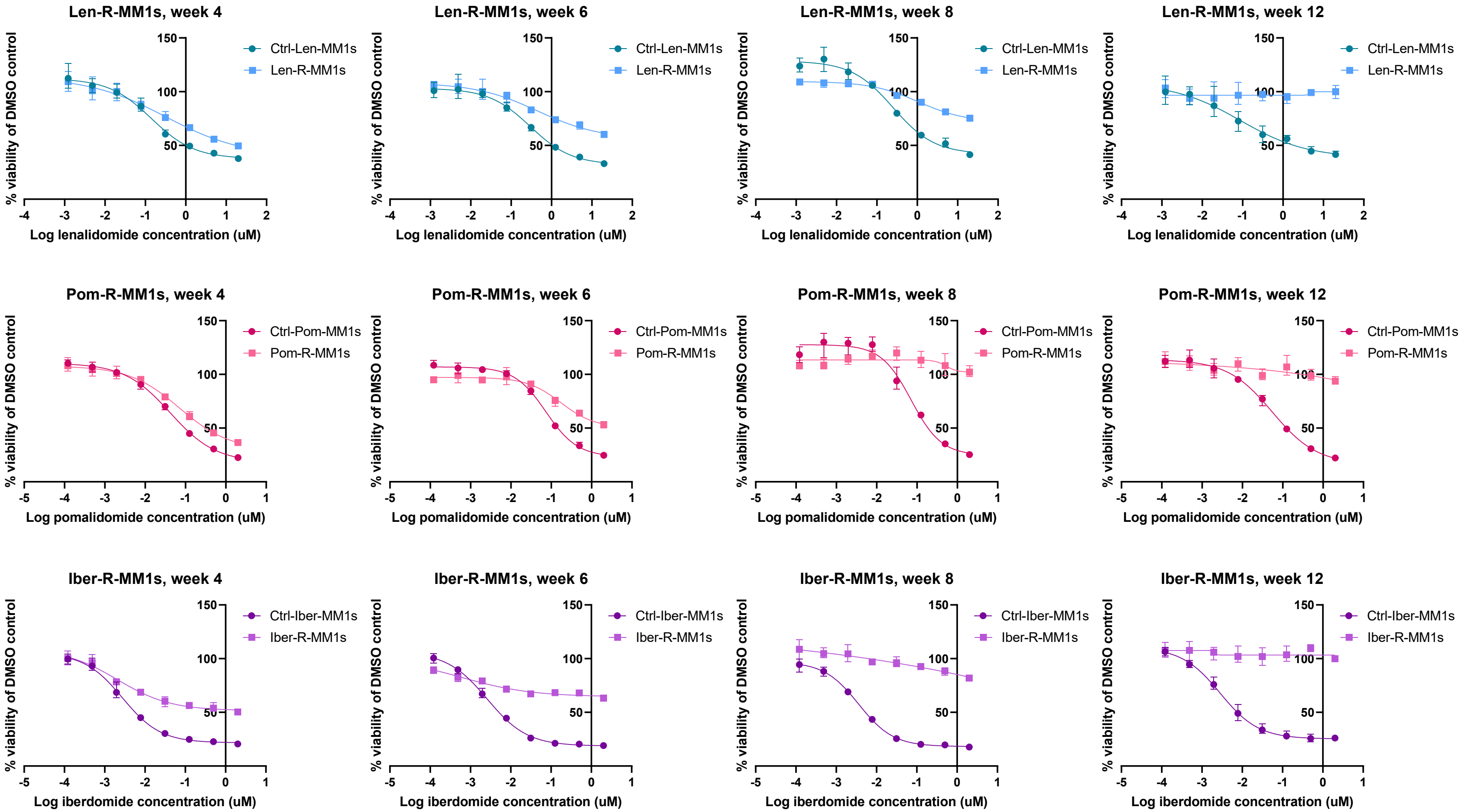

B)

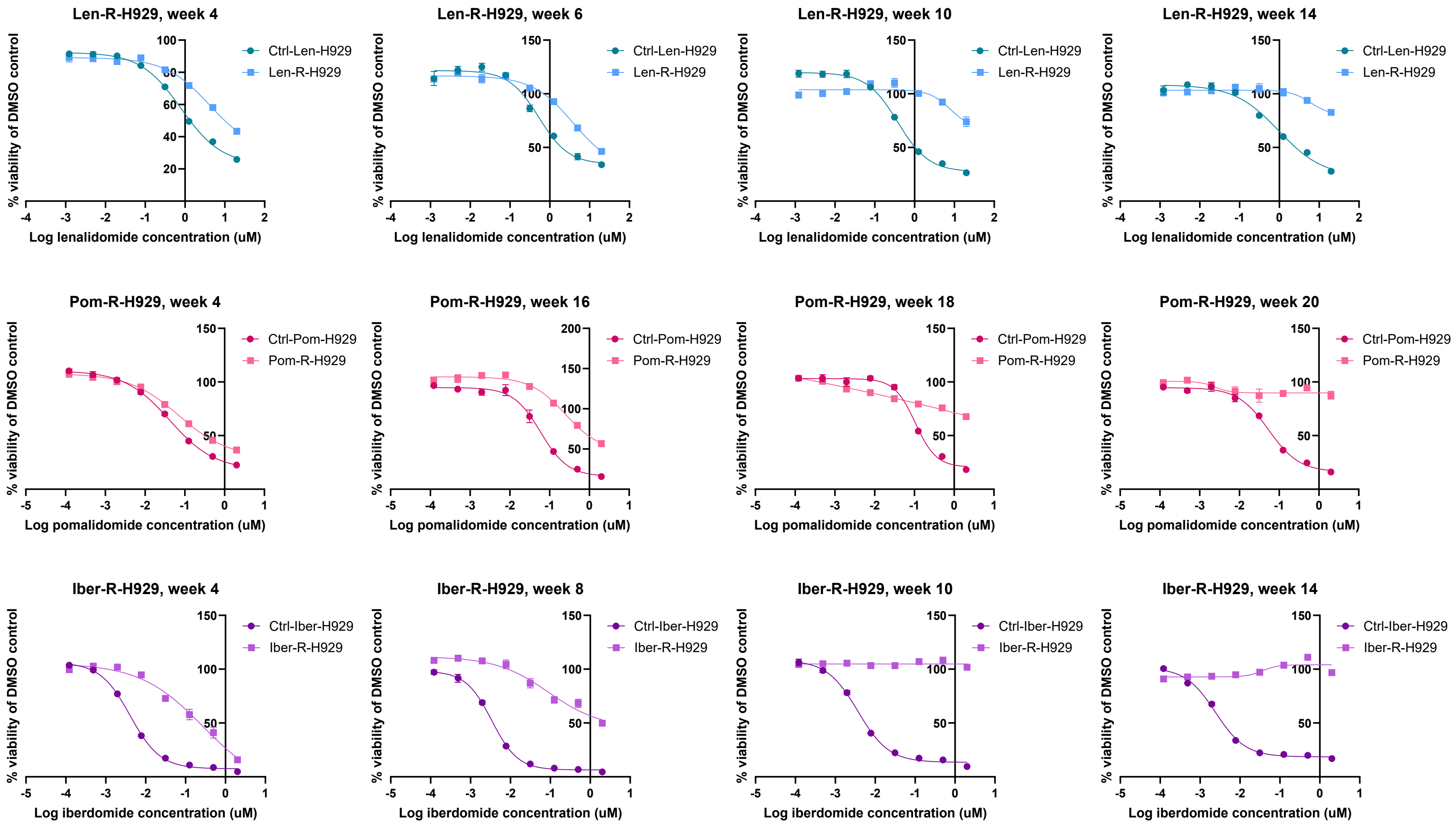
Len-R-H929, week 4
Len-R-H929, week 6
Len-R-H929, week 10
Len-R-H929, week 14
Ctrl-Len-H929
Len-R-H929
% viability of DMSO control
Log lenalidomide concentration (uM)
Pom-R-H929, week 4
Pom-R-H929, week 16
Pom-R-H929, week 18
Pom-R-H929, week 20
Ctrl-Pom-H929
Pom-R-H929
Log pomalidomide concentration (uM)
Iber-R-H929, week 4
Iber-R-H929, week 8
Iber-R-H929, week 10
Iber-R-H929, week 14
Ctrl-Iber-H929
Iber-R-H929
Log iberdomide concentration (uM)

Supplementary Figure 4: CRBN proteins levels

CRBN was reduced at the protein level in the resistant cell lines compared to control cell lines, as shown by A) immunoblot analysis and B) quantitative proteomics. A) Quantification of western blots (n=2) was performed using ImageStudio software (version 5.2). Graphs show mean and SEM and individual points represent independent biological repeats. Pairwise comparisons between groups were performed using unpaired t-tests, * = p<0.05, ** = p<0.01, *** = p<0.001. B) Proteomics data (n=3) was analysed by a limma moderated t-test in Phantasus (v1.19.3). Ctrl-MM1s = Ctrl-Iber-MM1s and Ctrl-H929 = Ctrl-Iber-H929). Graphs show mean and SEM and individual points represent independent biological repeats. ** adj p<0.01, *** adj p<0.001.

A)

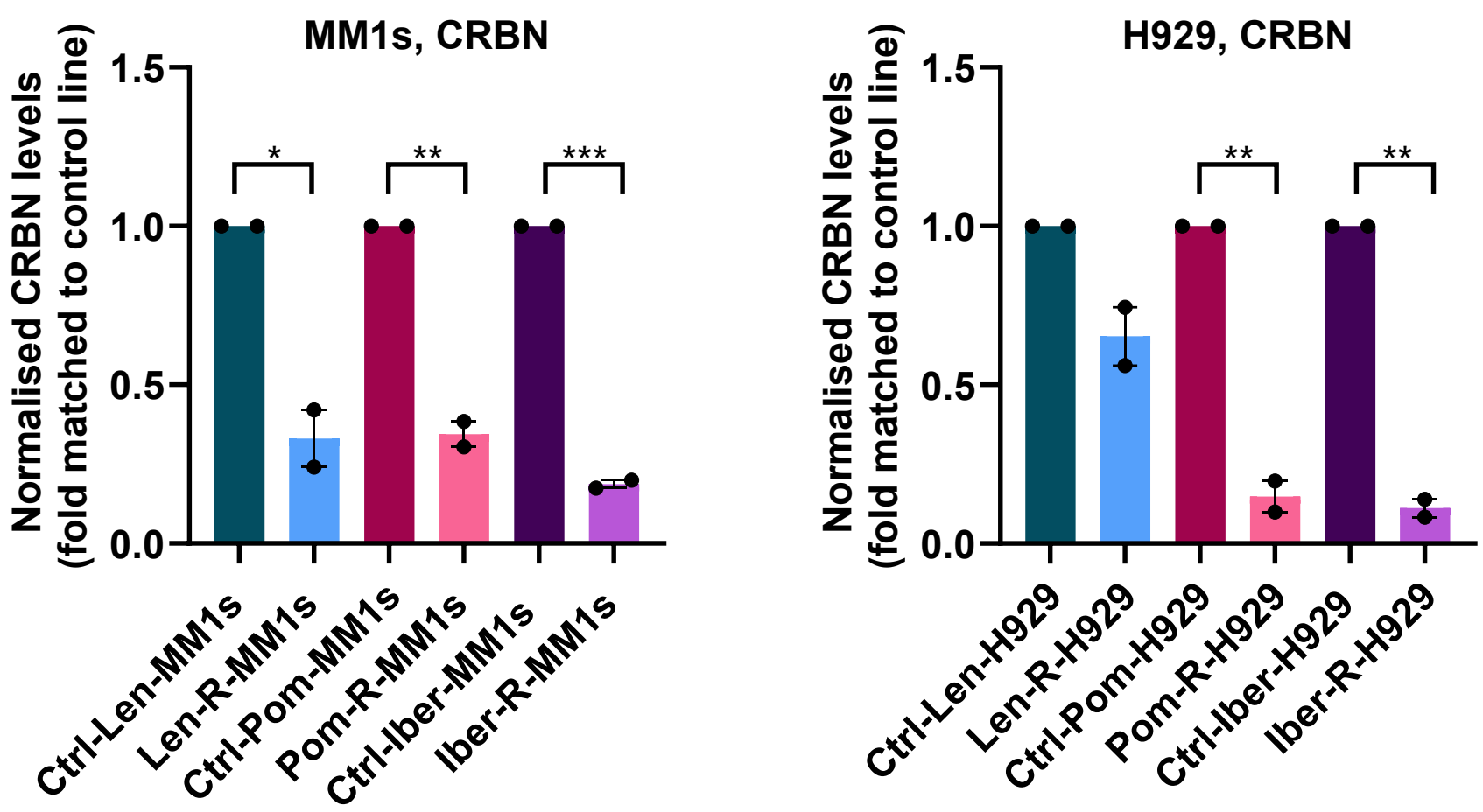

B)

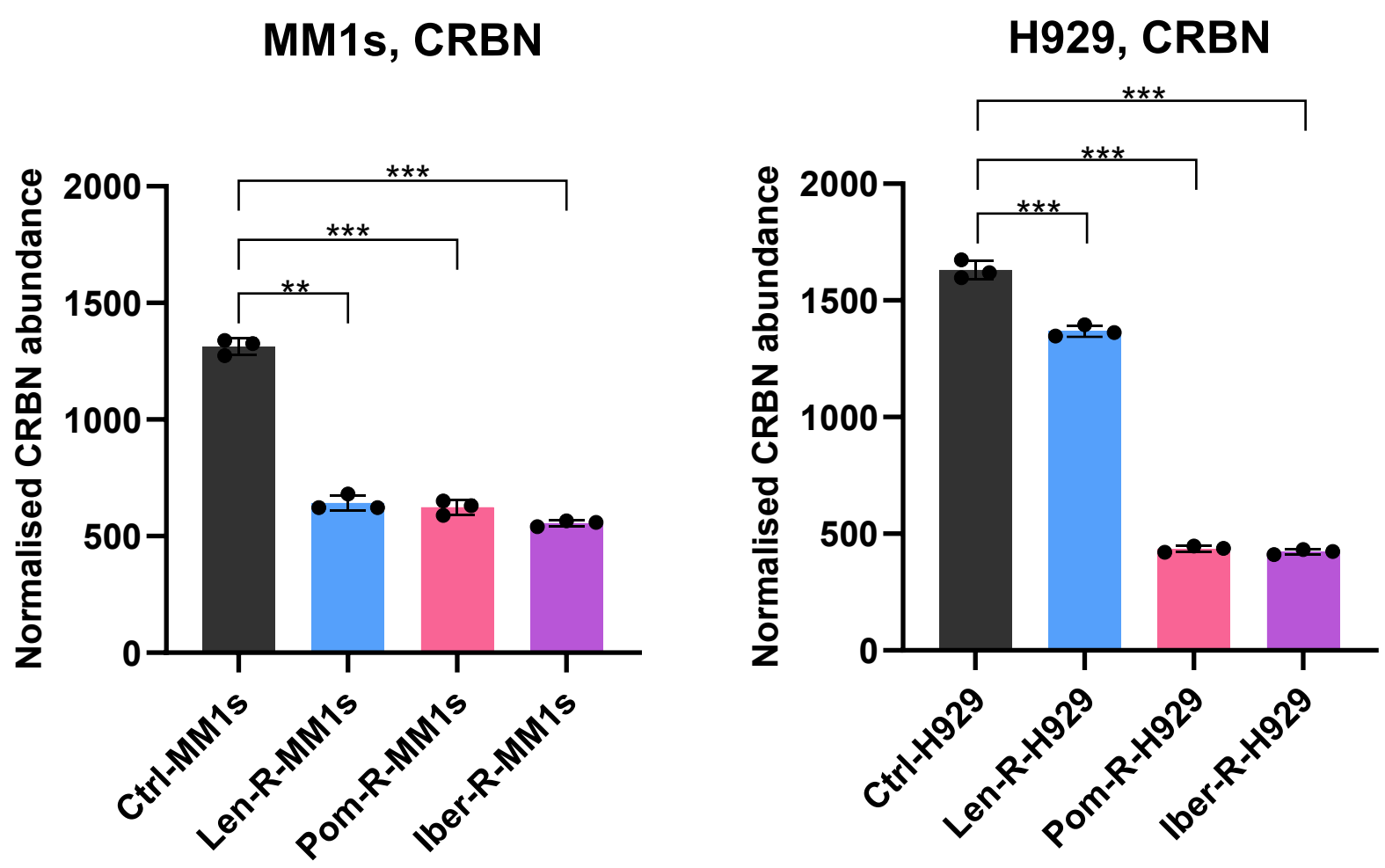

Supplementary Figure 5: Western blot quantitation demonstrating loss of CRBN function in resistant cell lines

Ikaors and Aiolos were degraded in the control lines with IMiD/CELMoD treatment, but this effect was abrogated in the resistant lines. Quantification of western blots was performed using ImageStudio software (version 5.2) and is shown for (A) MM1s cell lines (B) and H929 cell lines. Graphs show mean and SEM and individual points represent independent biological repeats (n=3). Pairwise comparisons between groups were performed using unpaired t-tests, ** = $p<0.01$, *** = $p<0.001$.

A)

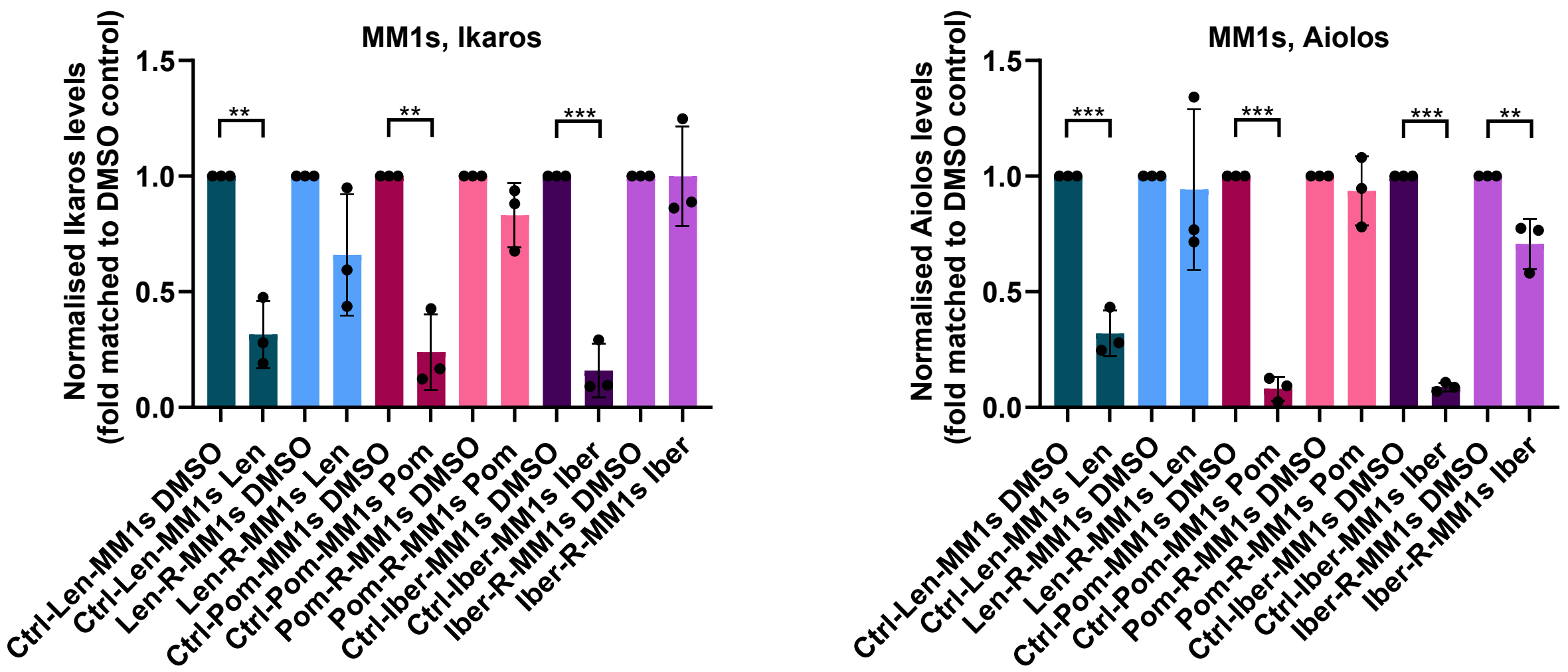

B)

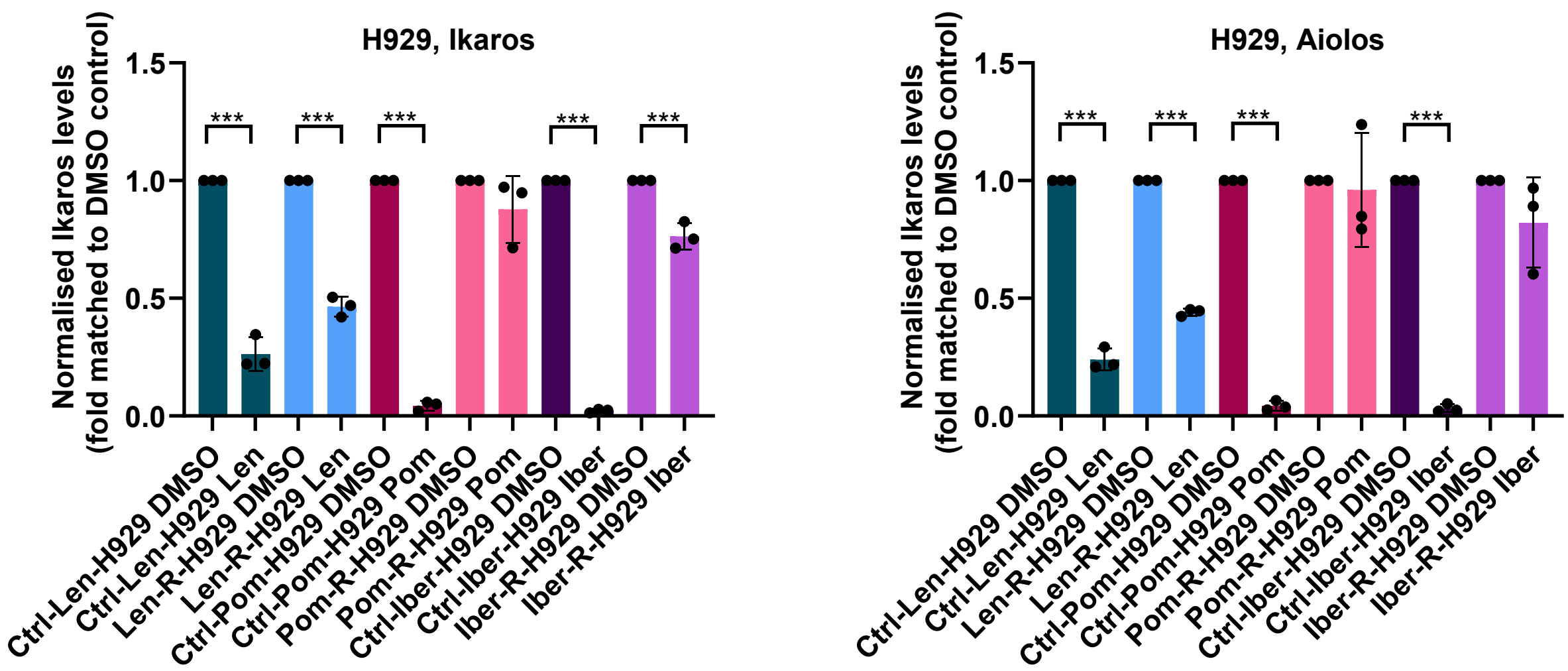

Supplementary Figure 6: Copy number loss at the *CRBN* locus in the resistant cell lines

Copy number loss was observed in Pom-R-H929, and to a lesser degree in Len-R-H929. Plots were generated by CNVKit; the grey circles represent individual bins (segments of the sequence), and the orange line indicates copy number change. The yellow vertical line shows the position of *CRBN* on chromosome 3 [9].

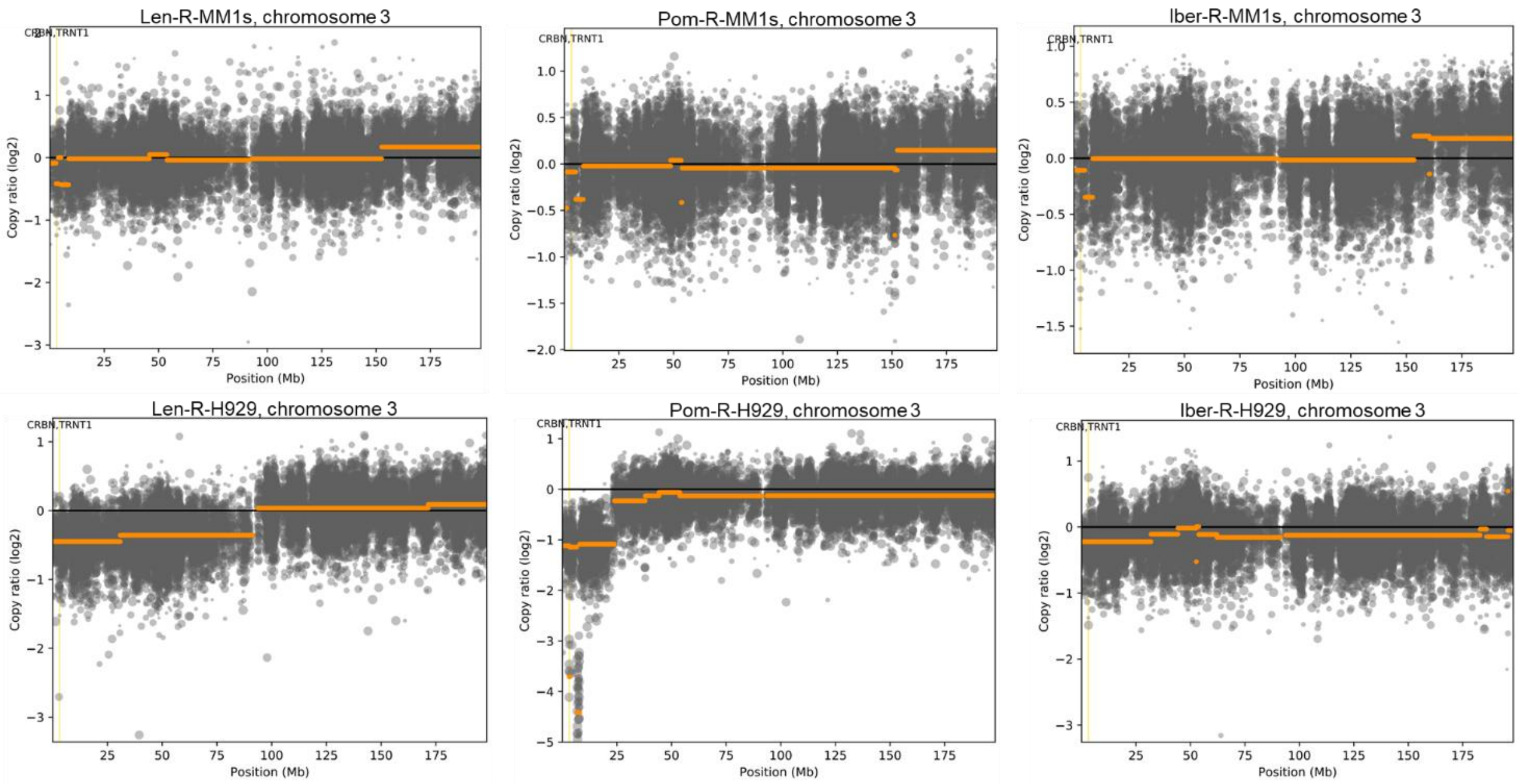

Supplementary Figure 7: Over-representation analysis of proteins with reduced expression in the IMiD/CELMoD resistant cell lines

Over-representation analysis was performed on statistically significantly altered (adj p<0.05) proteins with the most reduced expression compared to control (n=100) in each IMiD/CELMoD resistant lines. The control for the MM1s lines was Ctrl-Iber-MM1s and the control for the H929 lines was Ctrl-Iber-H929. Data for Iber-R-MM1s is shown in the main article, Figure 2C.

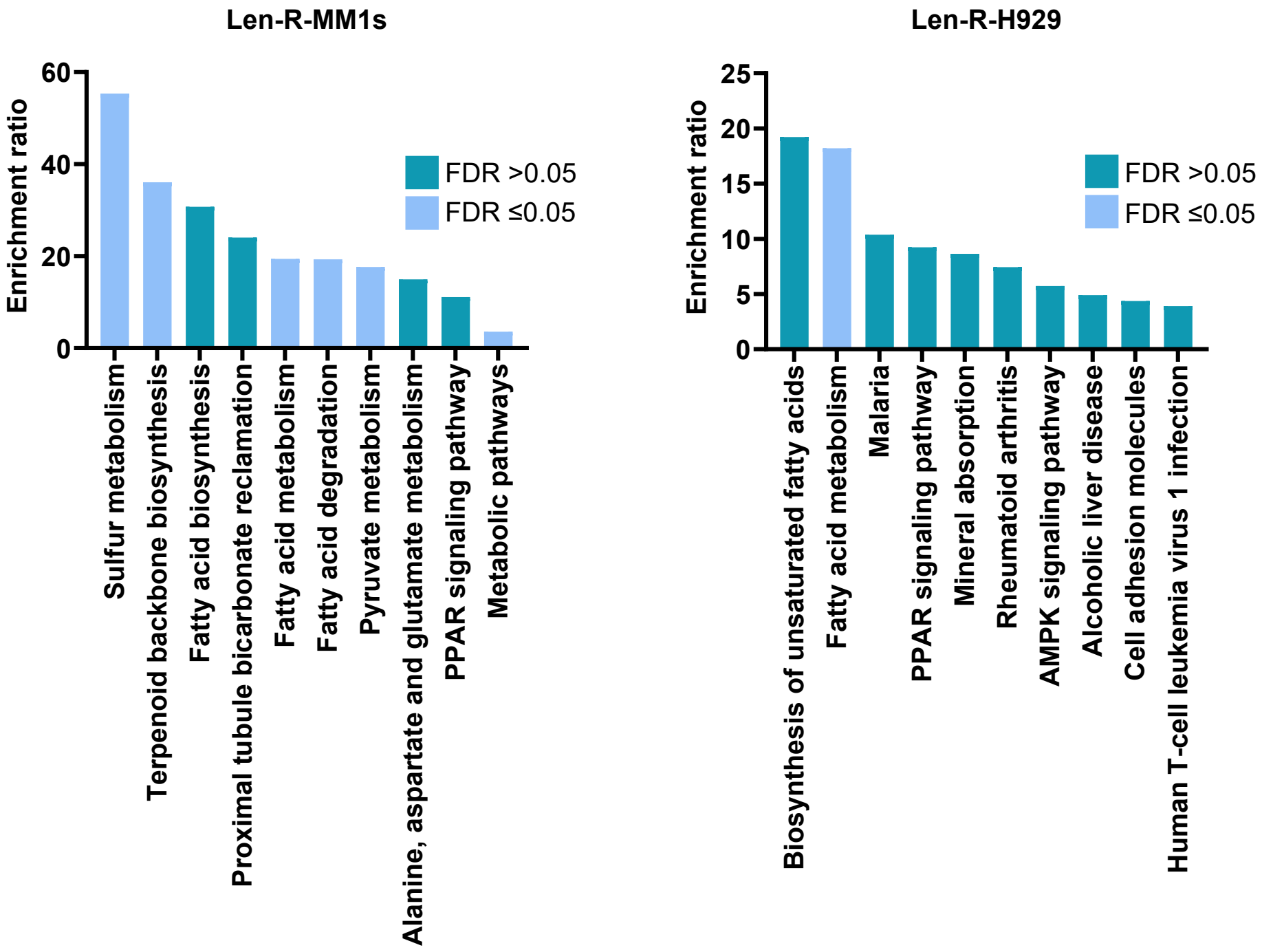

SUPPLEMENTARY MATERIAL

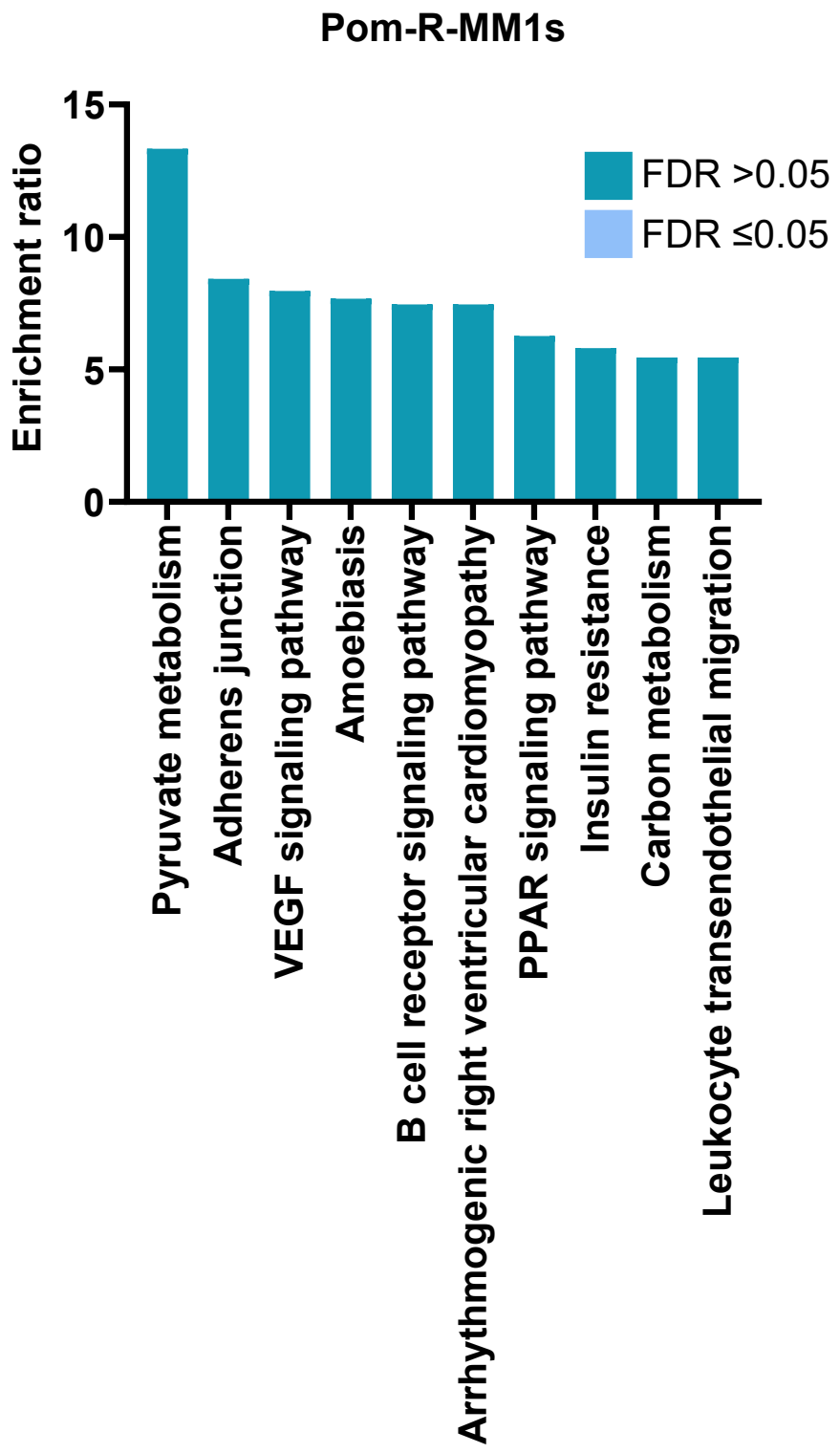

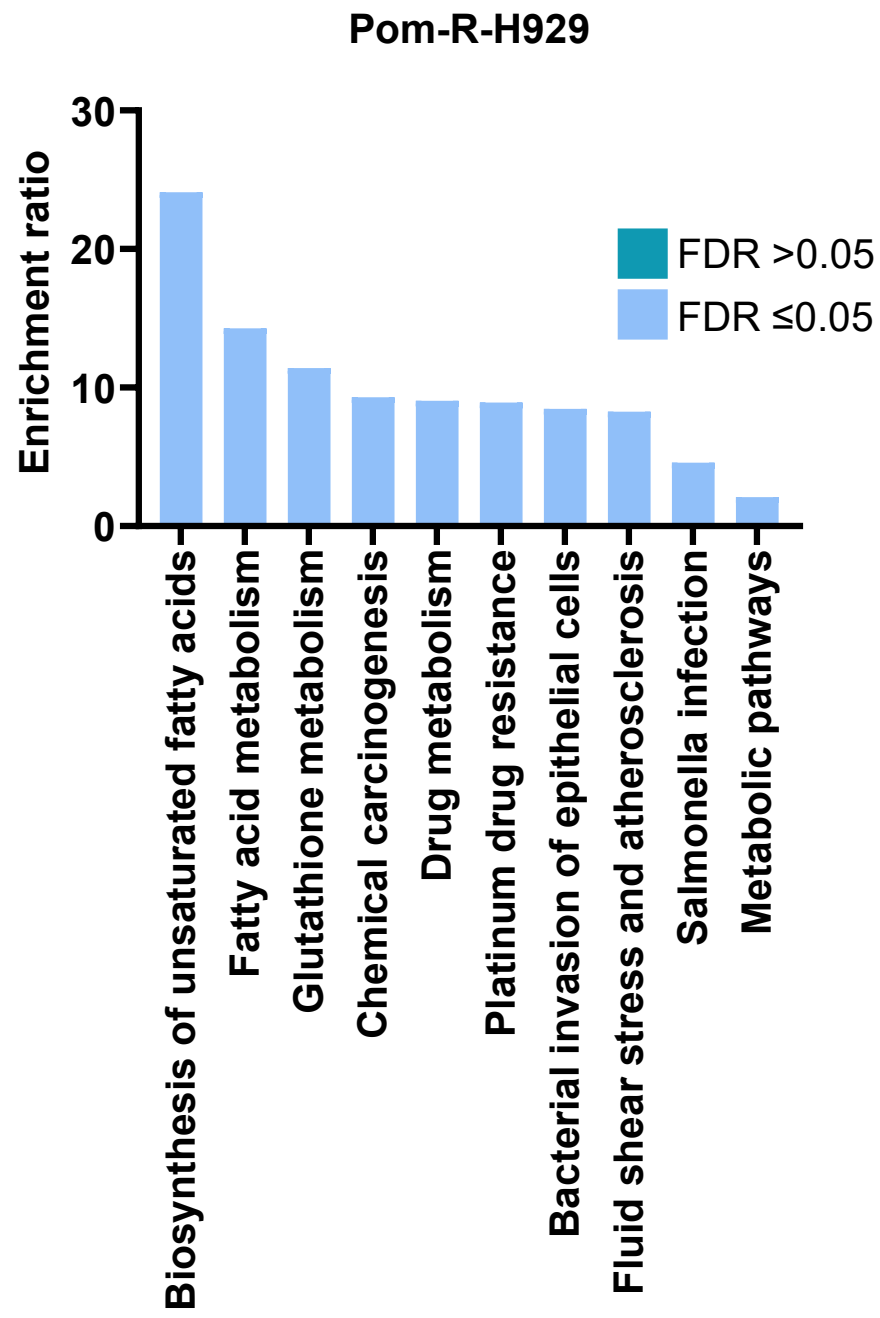

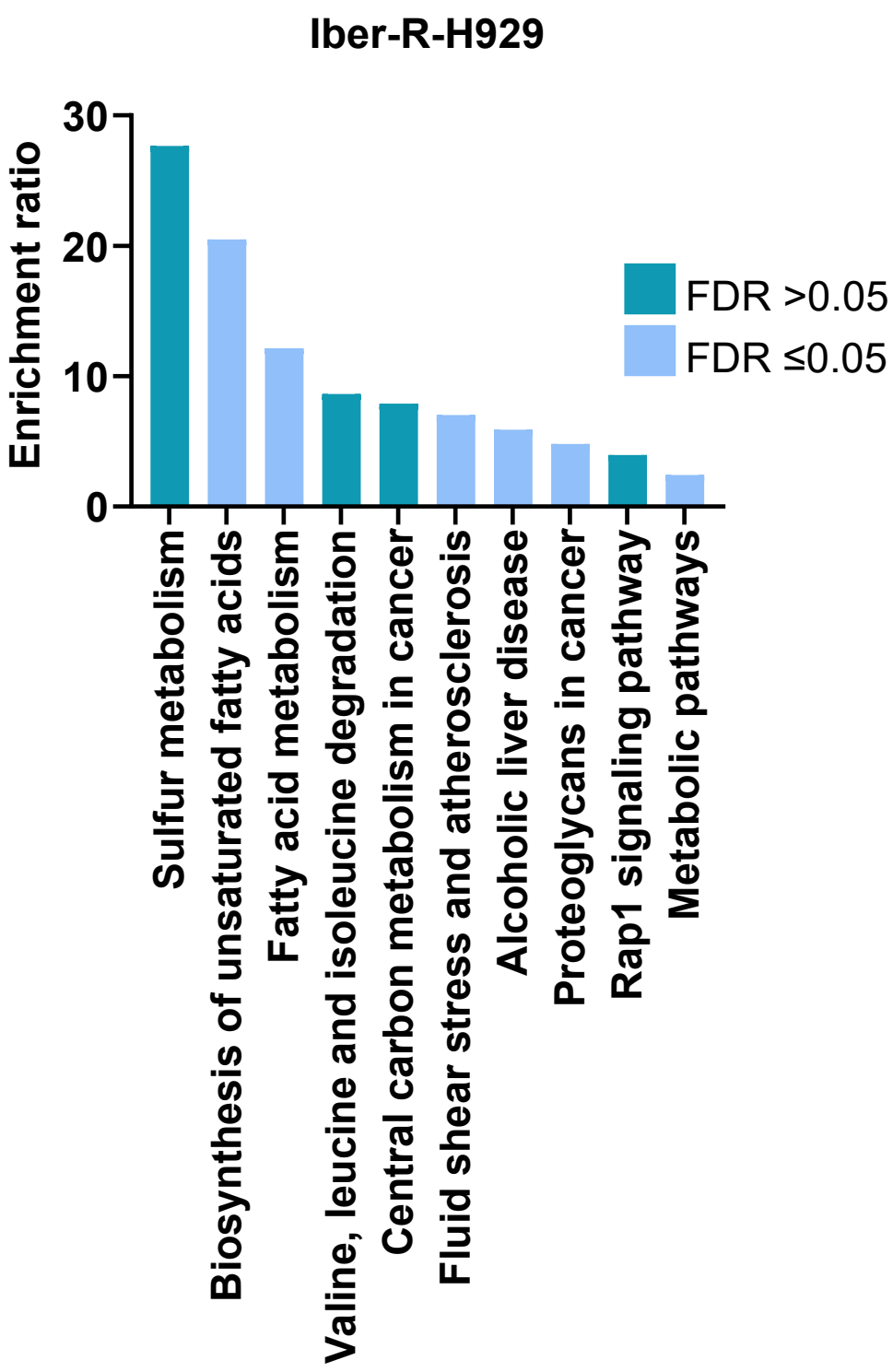

Supplementary Figure 8: Over-representation analysis of proteins with reduced expression in CRBN-KO-MM1s

Over-representation analysis was performed on statistically significantly altered (adj $p<0.05$) proteins with the most reduced expression in CRBN-KO-MM1s compared to parental MM1s (n=100).

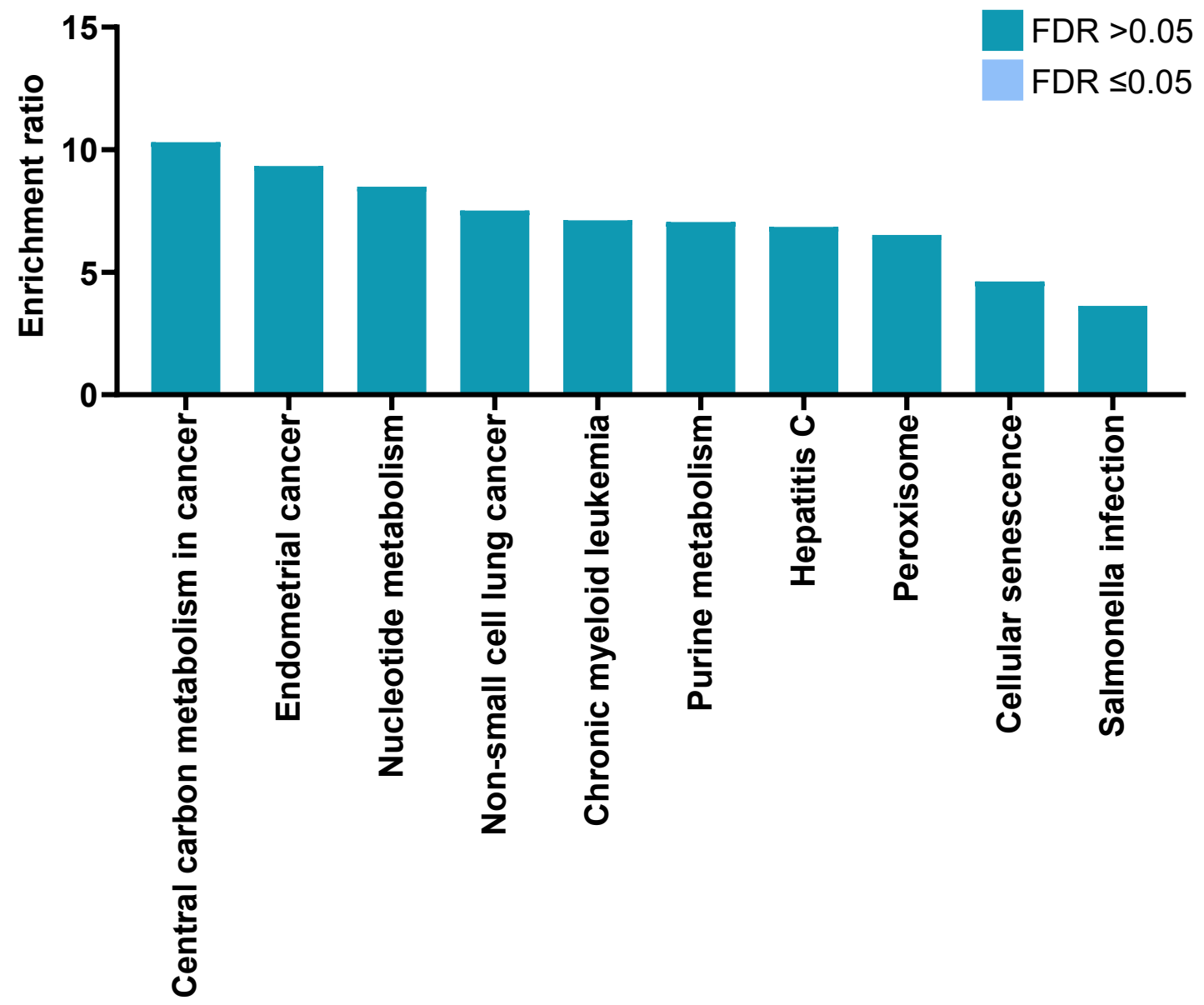

Supplementary Figure 9: Viability assays demonstrating response of the resistant cell lines to lovastatin treatment

A) Cell viability assays (CellTiter-Blue) with indicated cell lines incubated with lovastatin for 5 days. Mean and SEM shown, n=3. Data for Iber-R-MM1s and Iber-R-H929 are shown in the main paper (Figure 5). B) Cell viability assays (CellTitre-Blue) measured at Day 0 and 5 to compare cell proliferation. Mean and SEM shown, n=3. Pairwise comparisons between groups were performed using unpaired t-tests, * = $p<0.05$, ** = $p<0.01$ and *** = $p<0.001$. C) Annexin/PI flow cytometry with indicated cell lines incubated with lovastatin (1.25, 5 and 20μM) for 5 days. Mean and SEM shown, n=3. Pairwise comparisons between the proportion of cells in Q2 and Q3 in different groups were performed using unpaired t-tests, ** = $p<0.01$ and *** = $p<0.001$. D) Cell viability assays (CellTiter-Blue) exploring the combination of iberdomide and lovastatin. MM1s cell lines were exposed to 5μM of lovastatin and 8 concentrations of iberdomide (top concentration 2uM, 4-fold serial dilutions). H929 cell lines were exposed to 1.25μM of lovastatin and 8 concentrations of iberdomide (top concentration 2uM, 4-fold serial dilutions). The effect of single agent lovastatin cannot be inferred from these graphs. Mean and SEM shown, n=3.

A)

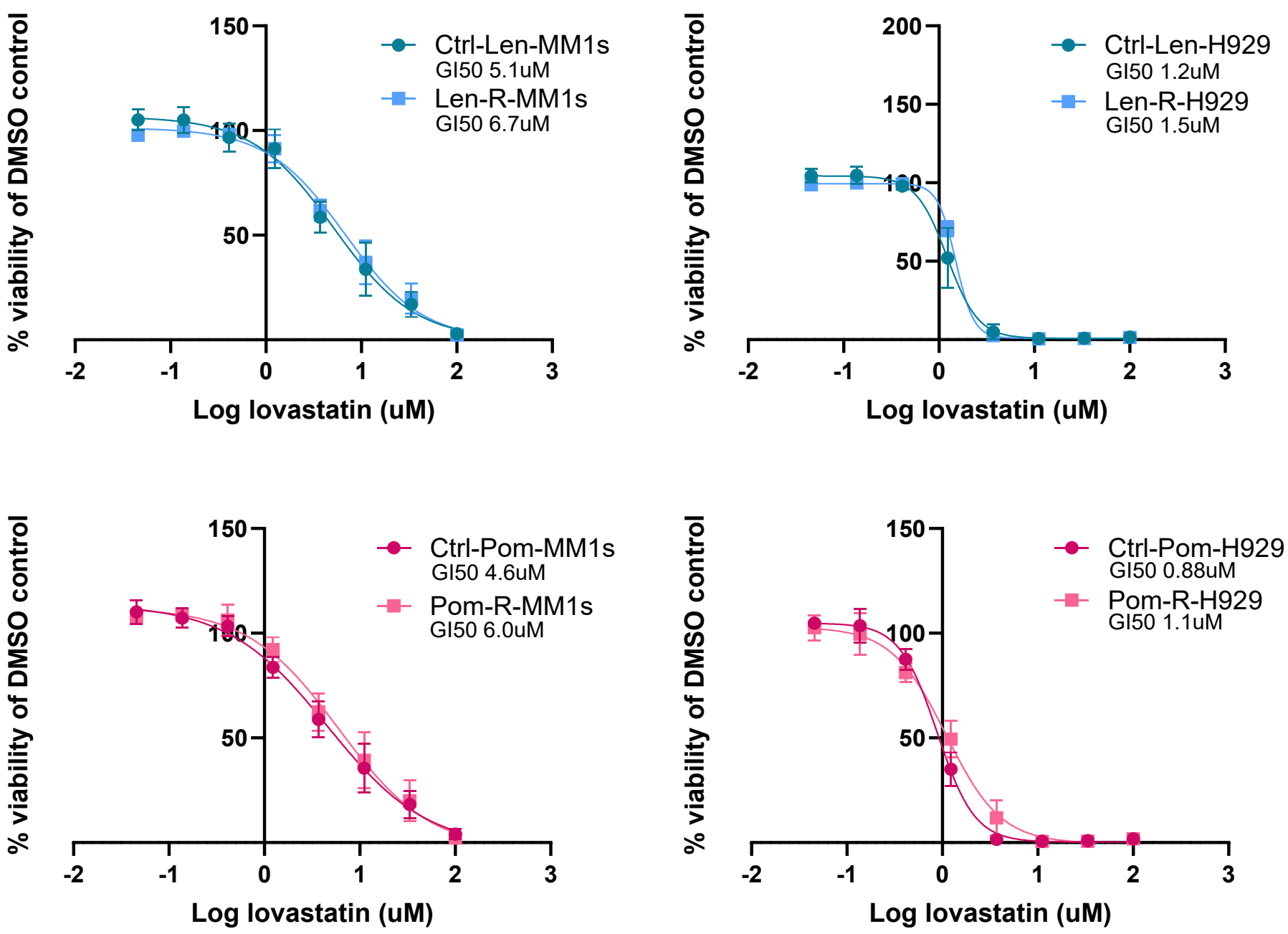

B)

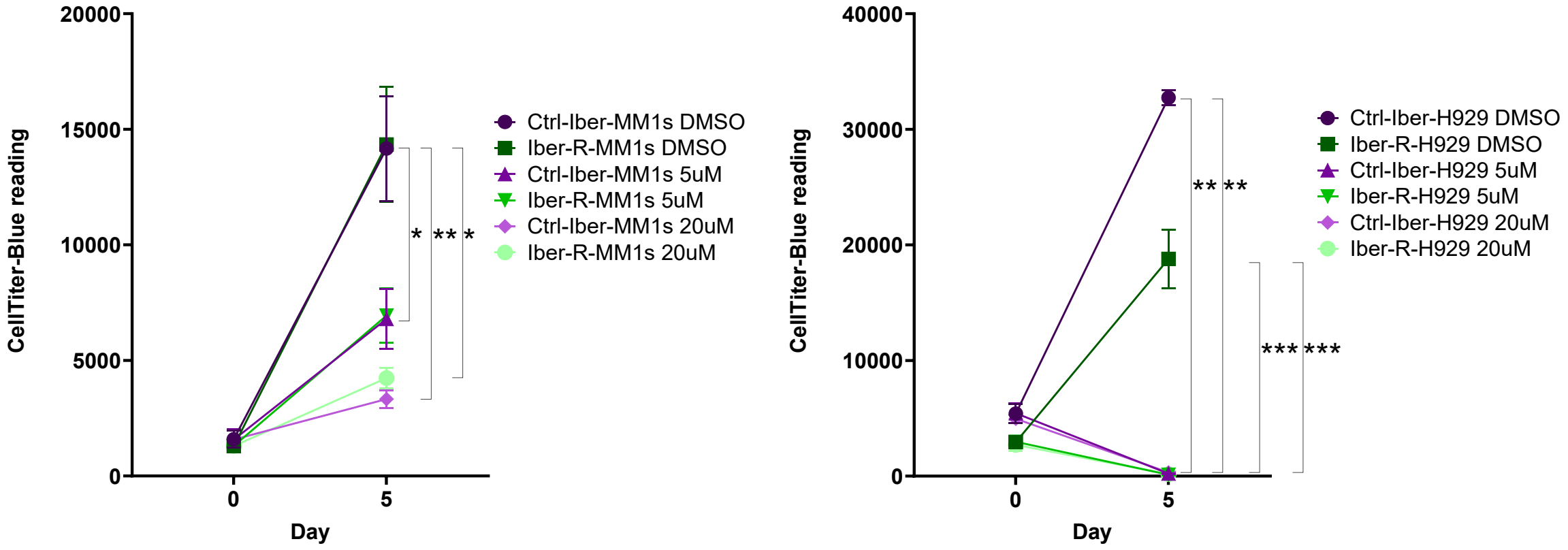
CellTiter-Blue reading
Day
Ctrl-Iber-MM1s DMSO
Iber-R-MM1s DMSO
Ctrl-Iber-MM1s 5uM
Iber-R-MM1s 5uM
Ctrl-Iber-MM1s 20uM
Iber-R-MM1s 20uM
Ctrl-Iber-H929 DMSO
Iber-R-H929 DMSO
Ctrl-Iber-H929 5uM
Iber-R-H929 5uM
Ctrl-Iber-H929 20uM
Iber-R-H929 20uM

C)

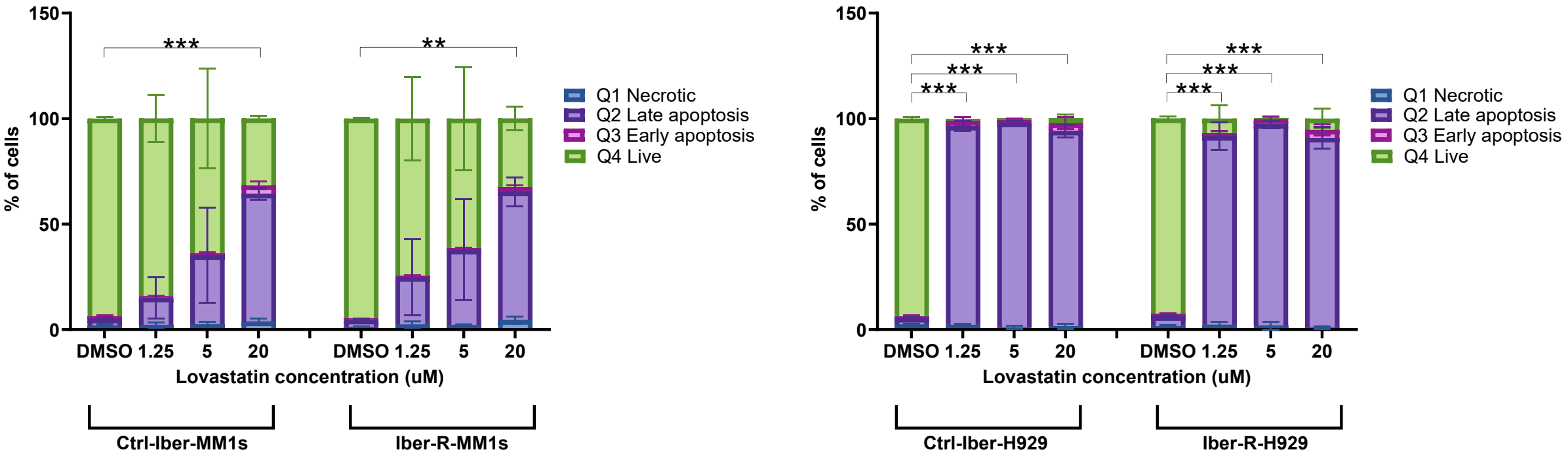
% of cells
Q1 Necrotic
Q2 Late apoptosis
Q3 Early apoptosis
Q4 Live
DMSO 1.25 5 20 DMSO 1.25 5 20
Lovastatin concentration (uM)
Ctrl-Iber-MM1s
Iber-R-MM1s
Ctrl-Iber-H929
Iber-R-H929

D)

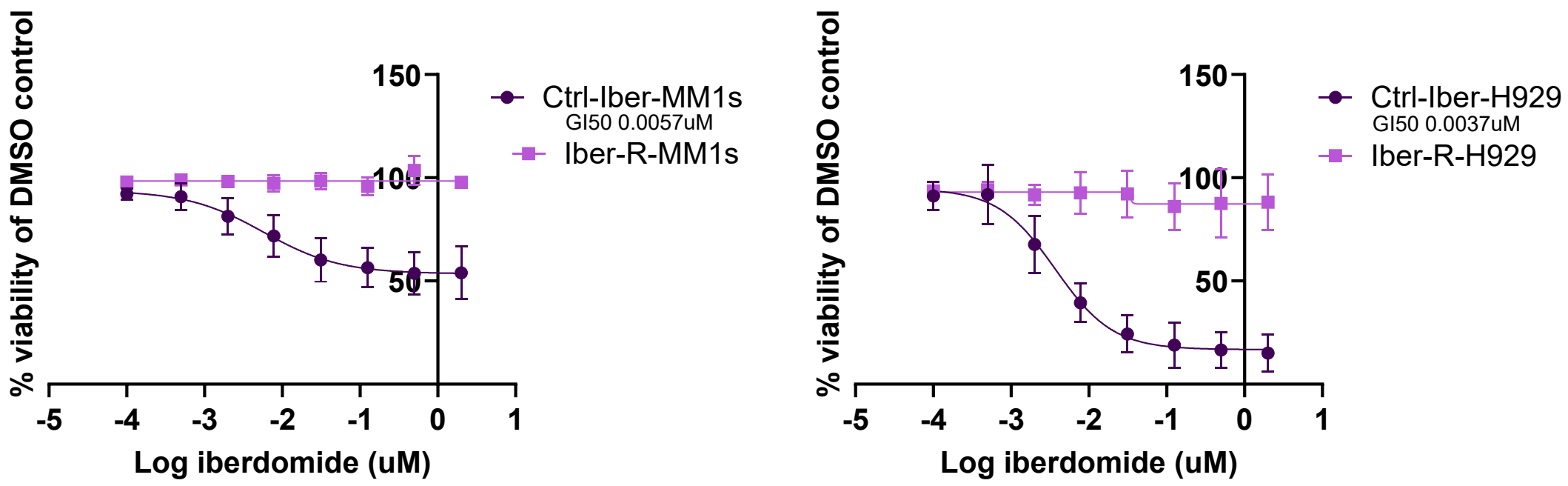
% viability of DMSO control
Log iberdomide (uM)
Ctrl-Iber-MM1s
GI50 0.0057uM
Iber-R-MM1s
Ctrl-Iber-H929
GI50 0.0037uM
Iber-R-H929

Supplementary Figure 10: Viability assays demonstrating response of the resistant cell lines to SSI-4 treatment

A) Cell viability assays (CellTiter-Blue) with indicated cell lines incubated with SSI-4 for 5 days. Mean and SEM shown, n=3. Data for Iber-R-MM1s and Iber-R-H929 are shown in the main paper (Figure 5). B) Cell viability assays (CellTitre-Blue) measured at Day 0 and 5 to compare cell proliferation. Mean and SEM shown, n=3. C) Annexin/PI flow cytometry with indicated cell lines incubated with SSI-4 (1.25, 5 and 20μM) for 5 days. Mean and SEM shown, n=3.

A)

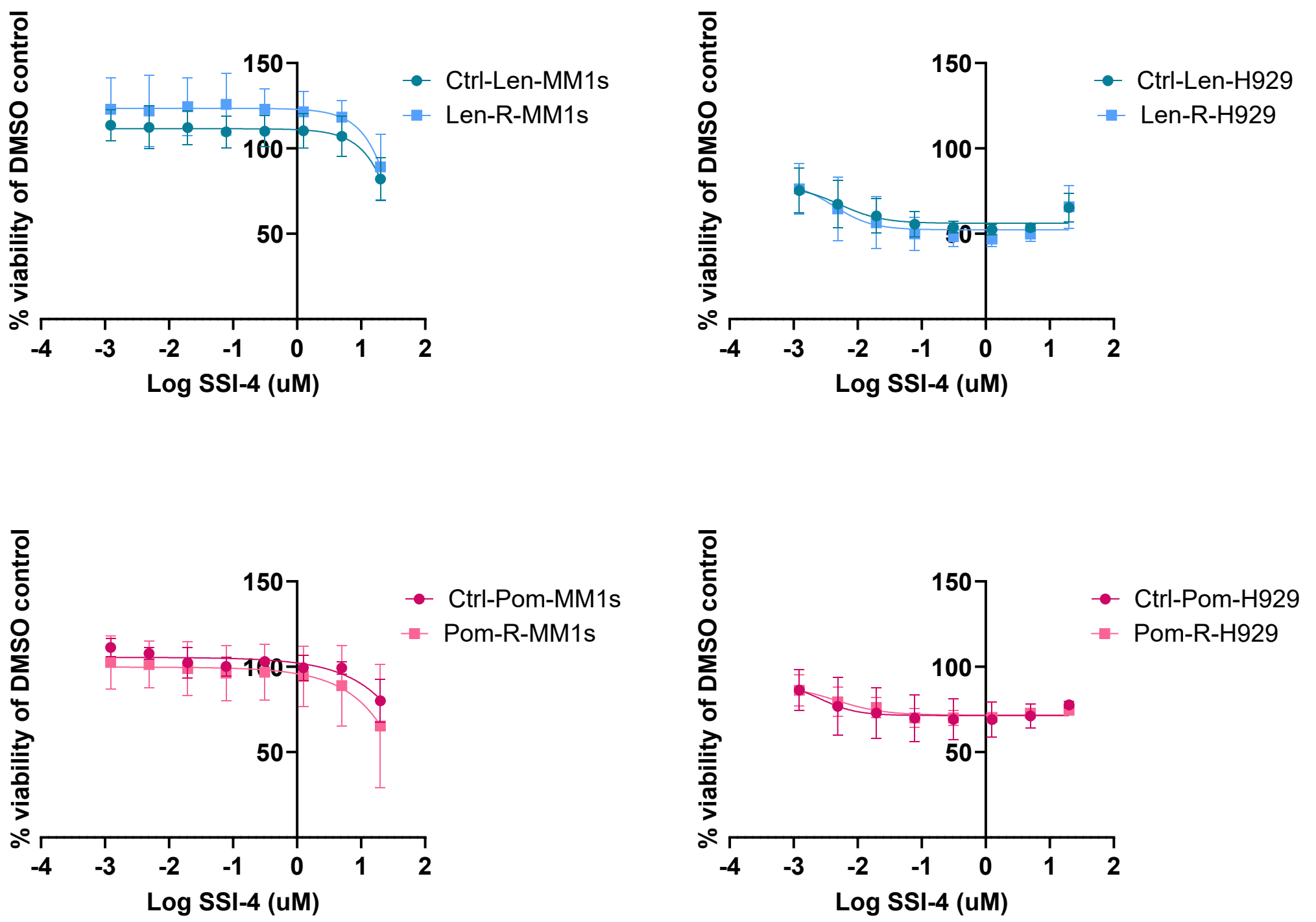

B)

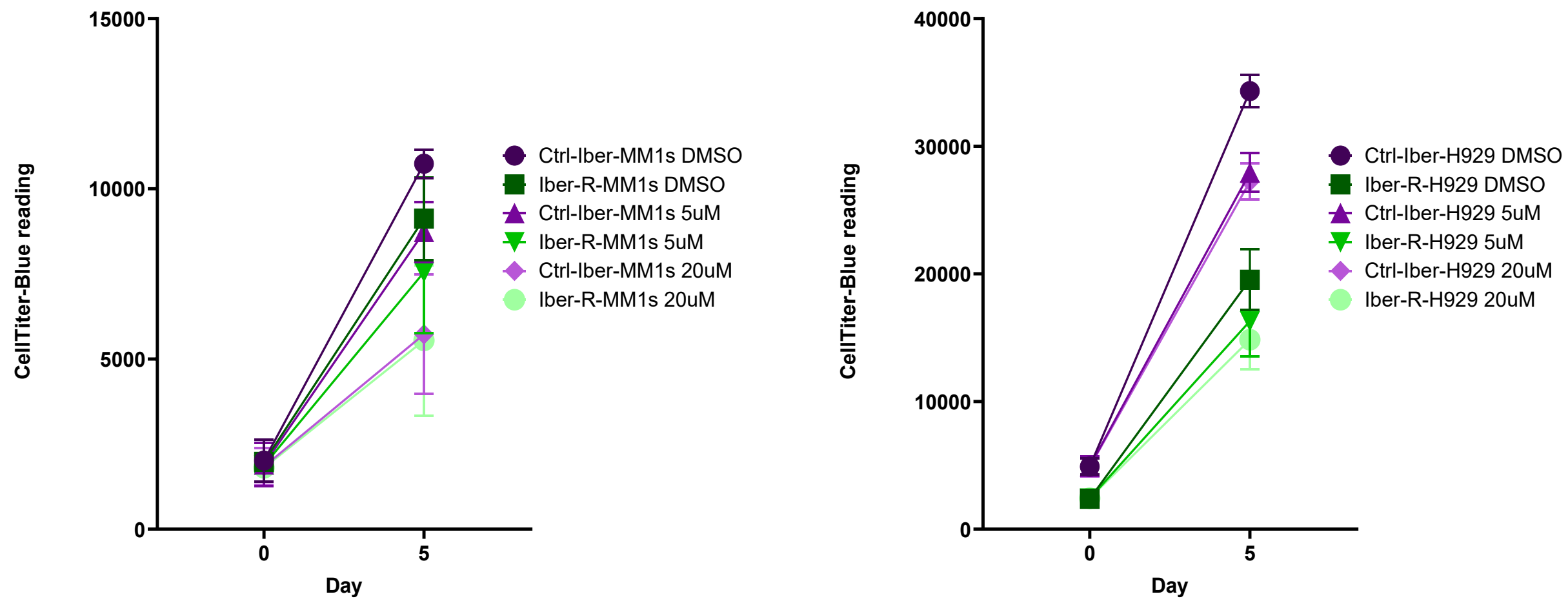

C)

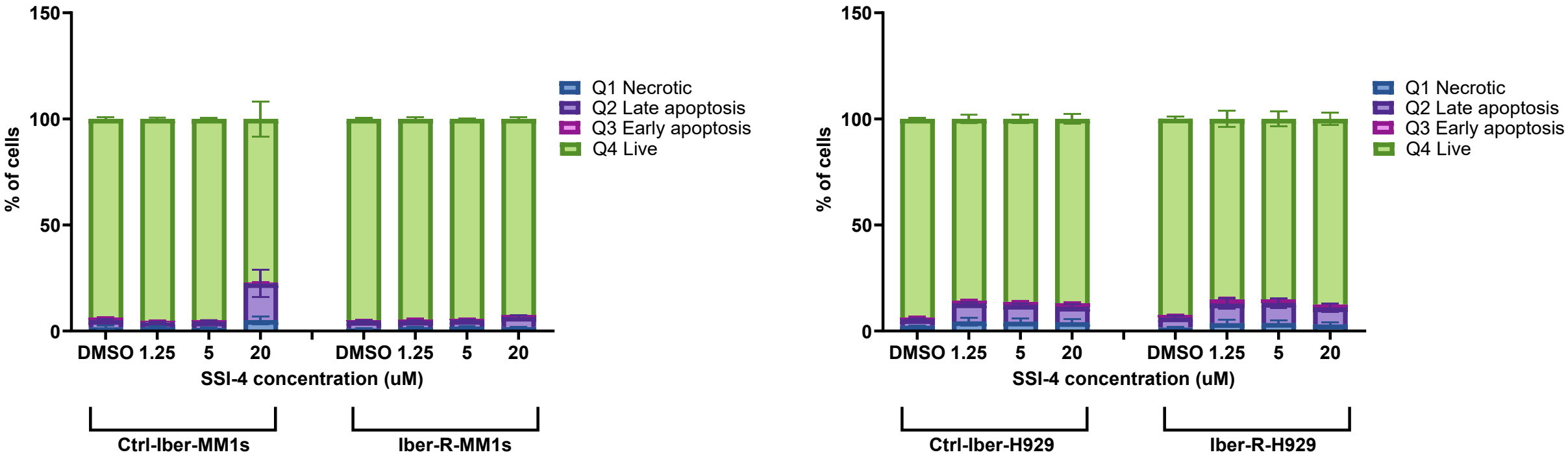
150
100
50
0
% of cells
DMSO 1.25 5 20 DMSO 1.25 5 20
SSI-4 concentration (uM)
Ctrl-Iber-MM1s
Iber-R-MM1s
Q1 Necrotic
Q2 Late apoptosis
Q3 Early apoptosis
Q4 Live
150
100
50
0
% of cells
DMSO 1.25 5 20 DMSO 1.25 5 20
SSI-4 concentration (uM)
Ctrl-Iber-H929
Iber-R-H929
Q1 Necrotic
Q2 Late apoptosis
Q3 Early apoptosis
Q4 Live

Supplementary Figure 11: Viability assays demonstrating response of the resistant cell lines to PF-429242 treatment

A) Cell viability assays (CellTiter-Blue) with indicated cell lines incubated with PF-429242 for 5 days. Mean and SEM shown, n=4. Data for Iber-R-MM1s and Iber-R-H929 are shown in the main paper (Figure 5). B) Cell viability assays (CellTiter-Blue) exploring the combination of iberdomide and PF-429242. MM1s cell lines were exposed to 5µM of PF-429242 and 8 concentrations of iberdomide (top concentration 2uM, 4-fold serial dilutions). H929 cell lines were exposed to 1.25µM of PF-429242 and 8 concentrations of iberdomide (top concentration 2uM, 4-fold serial dilutions). The effect of single agent PF-429242 cannot be inferred from these graphs. Mean and SEM shown, n=3.

A)

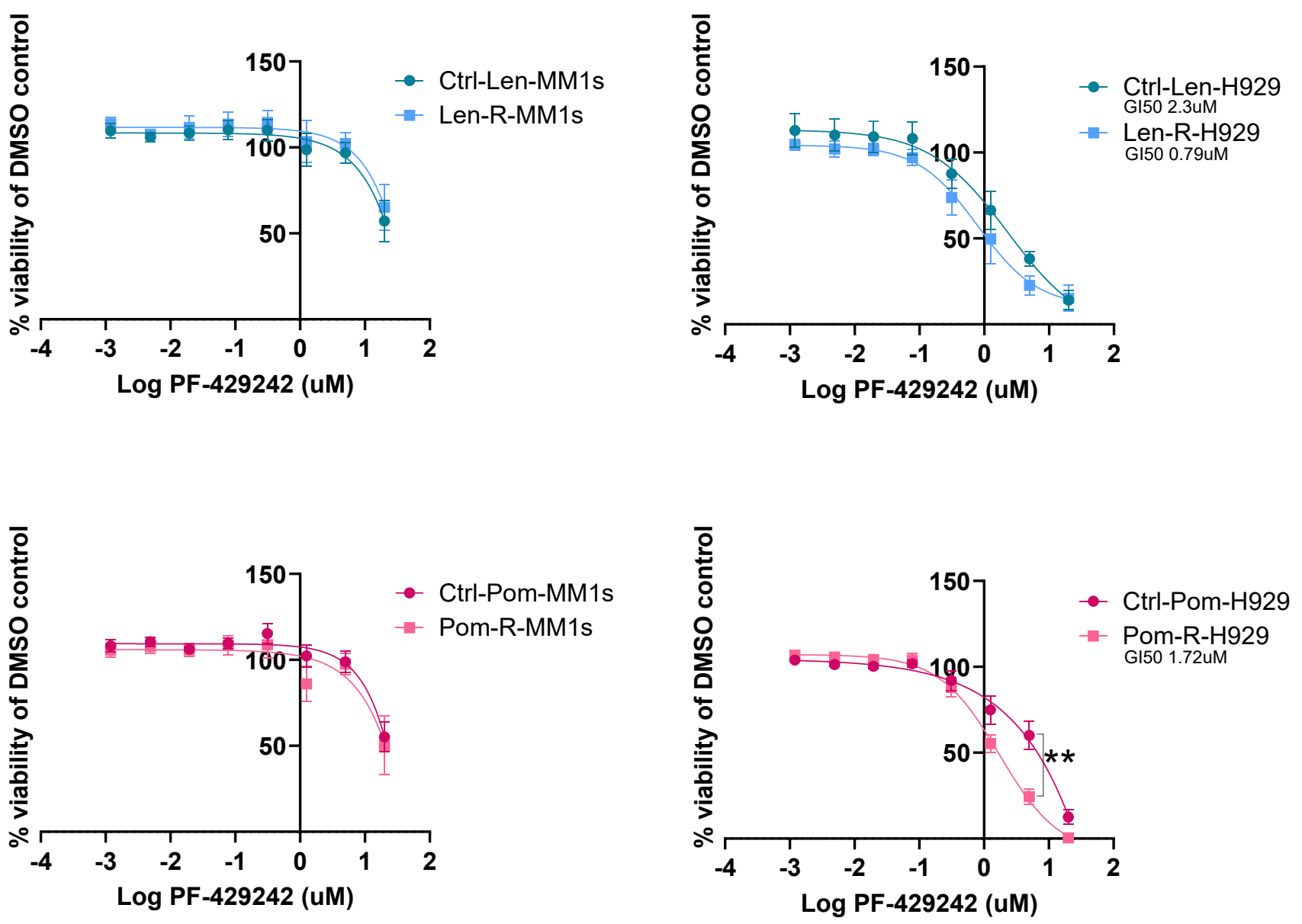

B)

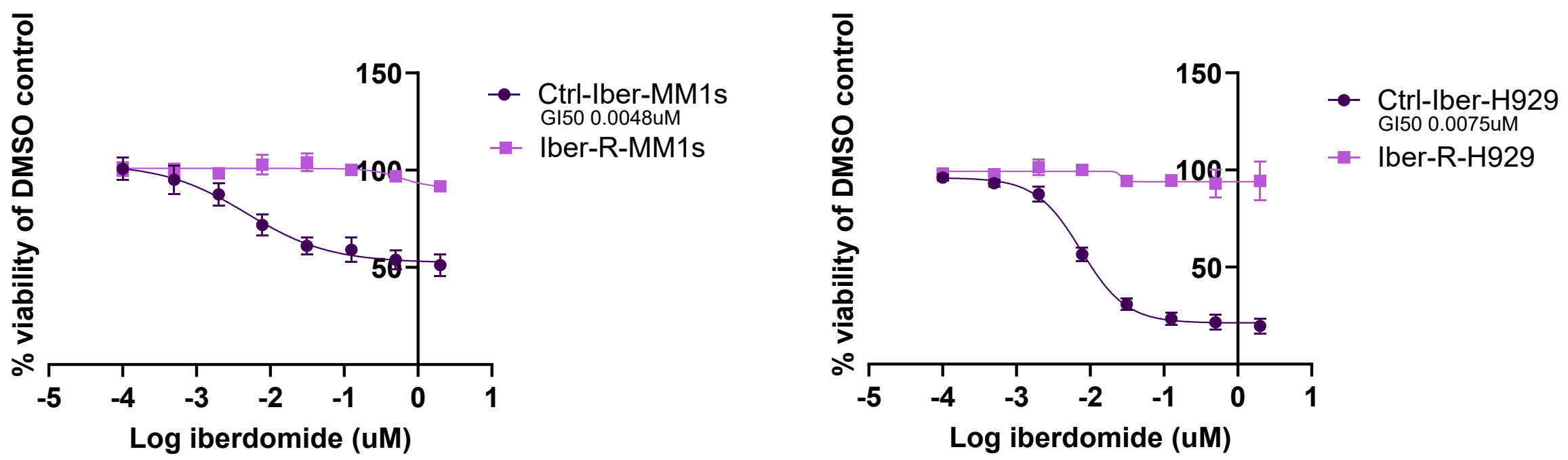

References

1. Sialana, F.J., et al., *SimPLIT: Simplified Sample Preparation for Large-Scale Isobaric Tagging Proteomics.* J Proteome Res, 2022. **21**(8): p. 1842-1856.
2. Zenkova, D., et al., *Phantasus: visual and interactive gene expression analysis*. 2018.
3. Stein and S, *An integrated method for spectrum extraction and compound identification from gas chromatography/mass spectrometry data*. 1999, J Am Soc Mass Spectrom. p. 770-781.
4. Behrends, V., G.D. Tredwell, and J.G. Bundy, *A software complement to AMDIS for processing GC-MS metabolomic data.* Anal Biochem, 2011. **415**(2): p. 206-8.
5. Tredwell, G.D. and H.C. Keun, *convISA: A simple, convoluted method for isotopomer spectral analysis of fatty acids and cholesterol.* Metab Eng, 2015. **32**: p. 125-132.
6. McLaren, W., et al., *The Ensembl Variant Effect Predictor.* Genome Biol, 2016. **17**(1): p. 122.
7. Stelzer, G., et al., *The GeneCards Suite: From Gene Data Mining to Disease Genome Sequence Analyses.* Curr Protoc Bioinformatics, 2016. **54**: p. 1.30.1-1.30.33.
8. Elizarraras, J.M., et al., *WebGestalt 2024: faster gene set analysis and new support for metabolomics and multi-omics.* Nucleic Acids Res, 2024. **52**(W1): p. W415-W421.
9. Talevich, E., et al., *CNVkit: Genome-Wide Copy Number Detection and Visualization from Targeted DNA Sequencing.* PLoS Comput Biol, 2016. **12**(4): p. e1004873.
